# Supplementary material for: Efficient photocatalytic production of hydrogen peroxide using dispersible and photoactive porous polymers
Source: Nat Commun. 2023 Oct 28;14:6891. doi: 10.1038/s41467-023-42720-6 (PMC10613291; doi:10.1038/s41467-023-42720-6)
Supplement: Supplementary file 1 — Supplementary Information [file 41467_2023_42720_MOESM1_ESM.doc]

Supplementary information

**Efficient photocatalytic production of hydrogen peroxide using dispersible and photoactive porous polymers**

Shengdong Wang^1,2^, Zhipeng Xie^3^, Da Zhu^4^, Shuai Fu^5^, Yishi Wu^6^, Hongling Yu^3^, Chuangye Lu^2^, Panke Zhou^3^, Mischa Bonn^5^, Hai I. Wang^5,7^, Qing Liao^6^, Hong Xu^4^, Xiong Chen^3^ & Cheng Gu^1^

*Corresponding to chenxiong987@fzu.edu.cn; gucheng@scu.edu.cn

**This PDF file includes:**

Table of Contents

Supplementary Materials and methods

Supplementary Figures S1 to S50

Supplementary Tables S1 to S28

Supplementary References S1 to S51

Table of Contents

**1. Supplementary Materials and methods........................................................................P3−P7**

Section 1: Syntheses of monomer, analog, and CTPs.........................................................................P3−P4

Section 2: Instruments and characterizations......................................................................................P4−P5

Section 3: Photocatalytic production of H_2_O_2_............................................................................................P5

Section 4: Theoretical calculation........................................................................................................P5−P6

Section 5: Exciton and charge-carrier dynamics.................................................................................P6−P7

**2. Supplementary Figures S1 to S50................................................................................P8−P57**

Section 1: ^1^H and ^13^C NMR and mass spectra of the monomer........................................................P8−P14

Section 2: Structural analyses of the CTPs......................................................................................P15−P24

Section 3: Solution processing of the CTPs.....................................................................................P25−P26

Section 4: Basic photophysical properties of the CTPs...................................................................P27−P35

Section 5: Photocatalytic production of H_2_O_2_..................................................................................P36−P48

Section 6: Theoretical studies...........................................................................................................P49−P55

Section 7: Exciton dissociation of the CTPs....................................................................................P56−P57

**3. Supplementary Tables S1 to S28..............................................................................P58−P102**

Section 1: Comparison of H_2_O_2_ generation performances of reported photocatalysts.............................P58

Section 2: Structural analyses of the CTPs.......................................................................................P59−P61

Section 3: Theoretical calculations...................................................................................................P62−P98

Section 4: Comparison of the E_b_ values of organic/polymer semiconductors................................P99−P101

Section 5: Transient kinetics of the CTPs...............................................................................................P102

**4. Supplementary References S1 to S51.....................................................................P103−P105**

**Supplementary Materials and Methods**

**Section 1: Syntheses of monomer, analog, and CTPs**

Materials

Dithiooxamide (Energy Chemical), 4-formylbenzonitrile (Energy Chemical), benzaldehyde (Energy Chemical), hydrogen peroxide (30% w/w, Guangzhou Chemical Reagent Factory), trifluoromethanesulfonic acid (99.9%, Energy Chemical), *n*-hexane (99.5%, Energy Chemical), tetrahydrofuran (99.5%, Energy Chemical), dichloromethane (99.9%, Energy Chemical), acetonitrile (99.9%, Energy Chemical), methanol (99.9%, Energy Chemical), *N*,*N*-dimethylformamide (99.5%, Energy Chemical), *N*,*N*-dimethylacetamide (99.9%, Energy Chemical), 1-methyl-2-pyrrolidinone (99.5%, Aldrich), anhydrous dimethyl sulfoxide (99.8%, J&K), 1,3-dimethyl-2-imidazolidinone (99.5%, Aldrich), acetic acid (AR, Shanghai Richjoint Chemical Reagents Co., Ltd.), hydrogen chloride (AR, Chron Chemicals), 2,2,6,6-tetramethylpiperidine (98%, Bede), 1,4-benzoquinone (97%, Tci), copper sulfate pentahydrate (99.8%, Bede), TiO_2_ (99%, Aladdin), and CF_3_COOD (Energy Chemical) for NMR were purchased and used without further purification.

O_2_ (99.9999%) and CO_2_ (99.9999%) were purchased from MULAI Company (China).

Syntheses of monomer TT–BN and analog TT–Bz

**Synthesis of 4,4'-(thiazolo[5,4-*d*]thiazole-2,5-diyl)dibenzonitrile (TT–BN).** Dithiooxamide (720 mg, 6.0 mmol) and 4-formylbenzonitrile (1810 mg, 13.8 mmol) were mixed in DMF (20 mL) in a 100-mL Shrek tube under nitrogen atmosphere in a glove box. The tube was sealed with a rubber stopper and heated at 150 °C for 12 h. After cooling, the precipitates formed were collected by filtration and washed by Soxhlet extraction in ethanol for another 12 h. Afterward, the solid was dried by vacuum evaporation for 24 h to afford the monomer TT–BN (1081 mg) as a light-yellow powder in a 53% isolated yield. ^1^H NMR (500 MHz, CF_3_COOD): δ (ppm) = 8.46 (4H, d, *J* = 8.2 Hz, Ph C_2_-*H*), 8.25 (4H, d, *J* = 8.2 Hz, Ph C_3_-*H*). ^13^C-NMR (125 MHz, CF_3_COOD): δ (ppm) = 134.79, 130.12, 127.18, 117.78, 115.52, 113.27, 111.02. MALDI-TOF MS: calcd. *m*/*z* = 344.41, found *m*/*z* = 344.02.

**Synthesis of 2,5-diphenylthiazolo[5,4-*d*]thiazole (TT–Bz).** Dithiooxamide (361 mg, 3.0 mmol) and benzaldehyde (0.608 mL, 6.0 mmol) were mixed in DMF (10 mL) in a 100-mL Shrek tube under the nitrogen atmosphere in a glove box. The tube was sealed with a rubber stopper and heated at 150 °C for 24 h. After cooling, the product was collected by filtration, washed with diethyl ether, and recrystallized from dichloromethane to obtain the analog TT–Bz (590 mg) as a pale-yellow powder in a 67% isolated yield. ^1^H NMR (500 MHz, CF_3_COOD): δ (ppm) = 8.22 (4H, d, *J* = 7.4 Hz, Ph C_3_-*H*), 7.98 (2H, t, *J* = 7.5 Hz, Ph C_1_-*H*), 7.88 (4H, t, *J* = 7.9 Hz, Ph C_2_-*H*). ^13^C-NMR (125 MHz, CF_3_COOD): δ (ppm) = 133.65, 127.67, 117.81, 115.57, 113.31, 111.06. MALDI-TOF MS: calcd. *m*/*z* = 294.03, found *m*/*z* = 294.39.

Syntheses of CTPs and preparation of their films

**Synthesis of TT–CTP.** Under the nitrogen atmosphere, TfOH (1 mL) was added dropwise to a 10-ml Shrek tube containing 100 mg of monomer TT–BN. The system was stirred at -10 °C for 1.5 h. Then, the solution was further stirred for 12 h at 60 ºC. Upon cooling, the reaction was quenched by a 2-M NaOH aqueous solution. After centrifugation three times with 10 mL NMP, the precipitates were filtered and washed with 2 M NaOH and deionized water. The collected solid was Soxhlet extracted with dichloromethane (12 h), tetrahydrofuran (24 h), and ethanol (12 h). The powder was dried under vacuum at 80 ºC to afford TT–CTP as a yellow powder (91 mg, yield = 91%).

**Synthesis of TTH–CTP.** To a solution of 3 mL concentrated HCl, 3 mL H_2_O was added to 100 mg of CTP. The suspension was stirred at 30 °C for 4 h. Upon cooling, the mixture was diluted with deionized water (50 mL), and the dark precipitate was collected by filtration, rinsed with water and methanol, and dried under high vacuum to give TTH–CTP.

**Preparation of CTP films.** 10 mg TTH–CTP and 3 mL formic acid solution was mixed and sonicated for 30 min to obtain a CTP solution with a concentration of 3.3 mg mL^−1^, which was then spin-coated on a 1 × 1 cm^2^ quartz substrate to form a thin film of TTH–CTP with uniform thickness. After annealing at 60°, the TTH–CTP film was immersed in a 20% ammonia water solution for deprotonation and dried in a vacuum oven at 80 °C for 10 h to afford a TT–CTP film with uniform thickness.

**Section 2: Instruments and characterizations**

General instrumental analysis

^1^H NMR spectra were recorded on a Bruker AVANCE HD III 500M NMR spectrometer, where the chemical shifts (δ in ppm) were determined with respect to tetramethylsilane (TMS) as an internal reference. The mass measurements for solid-state samples were carried out on a rapifleXTM MALDI-TOF/TOF mass spectrometer from Bruker Daltonik GmbH using 7,7,8,8-tetracyanoquinodimethane (TCNQ) as the matrix. Fourier-transform infrared (FT-IR) spectra were recorded on an IFS 66V/S Fourier transform infrared spectrophotometer. Solid-state ^13^C cross polarization magic angle spinning nuclear magnetic resonance spectra (^13^C CPMAS NMR) were recorded on a JEOL JNM-ECA600 MHz, 3.2 mm rotor, MAS of 20 kHz, recycle delay of 1 sec. Powder X-ray diffraction (PXRD) measurements were performed on a Rigaku SmartLab X-ray diffractometer using Cu−Kα radiation (*λ* = 1.54178 Å) in the 2*θ* range of 5−40° with a scanning rate of 5° min^−1^. Field-emission scanning electron microscopy (FE-SEM) was performed on a Zeiss Merlin operating at an accelerating voltage of 5.0 kV. High-resolution transmission electron microscopy (HR-TEM) images were obtained on a TEM JEOL 2100F with an acceleration voltage of 300 kV. UV-vis spectra were recorded on a Shimadzu UV-3600 spectrometer. Photoluminescence spectra were recorded on a Jobin-Yvon Spex Fluorolog-3 spectrofluorometer. Temperature-dependent photoluminescence spectra were recorded on a HORIBA Fluorolog-3 module fluorescence spectrometer. The photoluminescence decay profiles were measured by using time-correlated single photon counting (TCSPC) mode on Picosecond Lifetime Fluorescence Spectrometer (QUANTAURUS-TAU C11367-11). Thermogravimetric analysis (TGA) was performed on a Rigaku Thermo plus EVO2 under a nitrogen atmosphere with a temperature ramp of 5 °C min^−1^. CO_2_-sorption measurements were performed on BELSORP-max (BEL Japan, Inc.) automated volumetric sorption analyzers. The desired temperature of 195 K for CO_2_ sorption was controlled by a mixture of dry ice and isopropanol. Film thickness was recorded on a Veeco Dektak 150 atomic profiler. EPR spectra were measured on a Bruker E500 electron paramagnetic resonance spectrometer.

Photoelectrochemical measurements

Working photoelectrode was constructed by the CTP powders. Specifically, the CTP powder (5 mg) and Nafion (100 μL) were blended into DMF (900 μL) as stock solution. Subsequently, the as-prepared solution (10 μL) was drop-coated on a clean FTO glass, and the CTP-based photoelectrode could be obtained by drying the DMF in ambient conditions. Transient photocurrent response, Mott-Schottky plot, and electrochemical impedance spectroscopy (EIS) were conducted by a three-electrode system, using the CTP-based electrode as the working electrode, Pt foil (1.0 × 1.0 cm^2^) as the counter electrode, and Ag/AgCl as the reference electrode. Especially, the flat band potential could be obtained by plotting the tangent line of Mott-Schottky spectra. The intersection point of the tangent line and the a-axis can be applied as the conduction band.

**Section 3: Photocatalytic production of H_2_O_2_**

10 mg of CTP was added to a mixed solvent containing 18 mL deionized water and 2 mL benzyl alcohol in a glass bottle (50 mL), and the bottle was sealed with a two-way valve that connected to an O_2_ balloon. The photocatalyst was dispersed by ultrasonication for 5 mins and remained stirring at 900 rpm during the reaction period. The bottle was kept at 25 ºC by circulating water and was irradiated by a 300 W Xenon lamp.

The quantity of H_2_O_2_ was determined by the colorimetric method. Specifically, 1 mL Ti(SO_4_)_2_ solution was added into 5.0 mL of reaction solution containing produced H_2_O_2_. Then the mixed solution was transferred into a vessel and determined by UV-vis spectrophotometer.

Ti(SO_4_)_2_ solution was prepared as an indicator to interact with H_2_O_2_. In detail, 1g TiO_2_ was blended in 100 mL H_2_SO_4_ solution (98%) and thermally treated at 155 °C for 20 h under stirring. The Ti(SO_4_)_2_ reaction solution can be collected by filtering off the undissolved TiO_2_ powder. The Ti^4+^ abundant solution will become a yellow complex when it interacts with H_2_O_2_ as shown in the following equation: Ti(SO_4_)_2_ + H_2_O_2_ → H_2_[Ti_2_(SO_4_)_2_].

The generated benzaldehyde was determined by gas chromatography.

Taking into account the acceleration of H_2_O_2_ decomposition under irradiation, the stability of the produced H_2_O_2_ was assessed by measuring the degradation behavior of H_2_O_2_ generated during the reaction of the prepared samples. The mixed solution of H_2_O_2_ (1 mM, 20 mL) and photocatalyst (1 mg mL^-1^) was sonicated for 2 minutes, followed by purging the system with argon. Subsequently, the light source was turned on, and the residual H_2_O_2_ concentration was measured every 15 minutes to assess its stability.

**Section 4: Theoretical calculation**

Theoretical calculation

All the DFT calculations of model TT–CTP, TTH–CTP (protonated on TT moiety), and TTH–CTP (protonated on triazine moieties) were performed with Gaussian 16 (C 01) package suite^S1^. The structure of all molecules was optimized by using PBE0^S2,S3^ functional, the 6-31G (d)^S4,S5^ basis set was employed for all atoms and an implicit solvent model was used to reflect the solvation environment, and implemented using SMD^S6^ solvation model in Gaussian 16 package, water was used to represent the solvents in the SMD models. The frequency calculations were also conducted at the same level of theory to ensure all the optimized structures were at a local minimum on the potential energy surface (PES) and then the generated wave functional files were used to calculate the highest occupied molecular orbital (HOMO) and lowest unoccupied molecular orbital (LUMO) energies with the help of Multiwfn 3.8 program^S7^. All figures were plot in Visual Molecule Dynamic 1.9.3 (VMD)^S8^.

**Simulation of UV-vis spectra.** We adopted the TDDFT method, at the PBE0/6-31G (d) level, to calculate the excitation energies and oscillator strength of the excited states of TT–CTP, TT–CTP–O_2_, TTH–CTP, and TTH–CTP–O_2_. It can be seen from TDDFT calculations that all structures exhibit two/three detectable absorption bands and the strongest absorption originated from π⭢π* in the same spatial area. The electronic absorption spectrum of TT–CTP, TT–CTP–O_2_, TTH–CTP, and TTH–CTP–O_2_ and their respective contribution curves corresponding to excitations from singlet ground state (S_0_) to various excited states are plotted, only the excitations with oscillator strength larger than 0.18 are considered. Percentage contributions of different excitations to the maximum absorption peak are also labeled selectively.

**Electrostatic Potential (ESP) Analysis.** ESP was analyzed with the help of Multiwfn 3.8 based on the generated wave functional files above and the results were plotted by VMD package^S9^.

**Adsorption energy analysis.** All calculations were conducted under the Gaussian 16 (C 01) package suite and the adsorption energy of CTP and CTP-H were calculated based on the equation: *E*_ads_ = *E*_total_ – *E*_sub_ – *E*_O₂_, where the *E*_sub_ and *E*_O₂_ represent the optimal structures of fragmented CTPs and its corresponding energies, respectively. *E*_total_ is the total energy of the optimal substrates absorbed by O_2_. All pictures were implemented by CYLview^S10^.

**Section 5: Exciton and charge-carrier dynamics**

Exciton binding energy

The photoluminescence intensity verse temperature plot could be fitted using

$I(T)=\frac{I_{0}}{I+Ae^{(-E_{B}/k_{B}T)}}$ (1)

in which *I*_0_ is the photoluminescence intensity at 0 K, *k_B_* is the Boltzmann constant, and *E_B_* is exciton binding energy.

Terahertz measurements

Time-resolved terahertz spectroscopy (TRTS) was employed to investigate the charge transport properties of CTPs. The TRTS setup is driven by a commercial Ti:sapphire laser amplifier system, which delivers a 1.55-eV pulsed laser with a duration of ~50 fs and a repetition rate of 1 kHz. The 1.55-eV pulsed laser was frequency-doubled through a BiB_3_O_6_ crystal to generate a 3.1-eV pulsed laser for photoexcitation. THz generation and detection were achieved by optical rectification and free-space electro-optic sampling, respectively. The time- and frequency-resolved photoconductivity measurements were performed in a nitrogen environment at room temperature.

The frequency-resolved complex photoconductivity was obtained by Fourier transforming the time-dependent THz field into the frequency domain and applying the thin-film approximation via the following equation:

$\Delta\sigma(\omega)=-\frac{n+1}{Z_{0}\cdot l}\cdot\frac{\Delta E(\omega)}{E}$ (2)

where *l* is the excitation length.

Transient absorption spectroscopy measurements

A femtosecond laser system (Pharos, Light Conversion) delivered laser pulses at 1030 nm (180 fs, 6 kHz), which were then divided into two components by using a 9:1 beam splitter. The major component was sent to an optical parametric amplifier (Orpheus, Light Conversion) to generate the pump pulses (500 nm, 6 kHz). The minor component was further attenuated and focused into a 3-mm sapphire plate to generate the probe pulses. Both the pump and probe pulses were guided into a Harppia spectrometer and time-resolved spectral data were recorded. A short-pass filter was inserted into the probe beam to cut off the fundamental light of 1030 nm. The time delay between the pump and probe beams was regulated through a computer-controlled motorized translation stage in the probe beam. The temporal resolution between the pump and the probe pulses was determined to be ~200 fs (FWHM). The transmitted light was detected by a CMOS linear image sensor. The excitation pulsed energy was ~ 60 nJ pulse^−1^ as measured at the sample site. The stability of the samples was spectrophotometrically checked before and after each experiment. Analysis of the kinetic traces derived from time-resolved spectra was performed using nonlinear least-square fitting to a general sum-of-exponentials function after deconvolution of the instrument response function (IRF). All the spectroscopic measurements were carried out at room temperature.

**Supplementary Figures**

**Section 1: ^1^H and ^13^C NMR and mass spectra of the monomer**

**
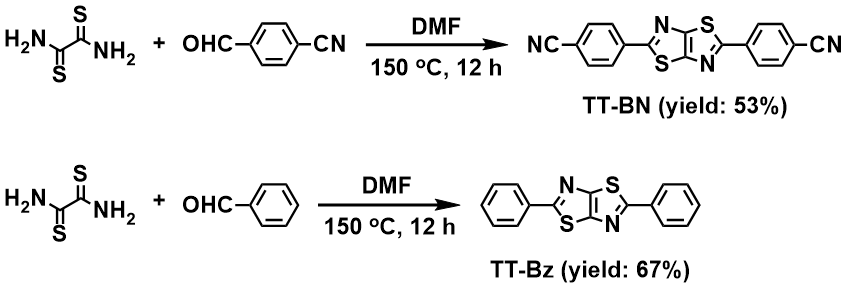
**

**Supplementary Figure S1.** Synthetic routes of TT–BN and TT–Bz.

**
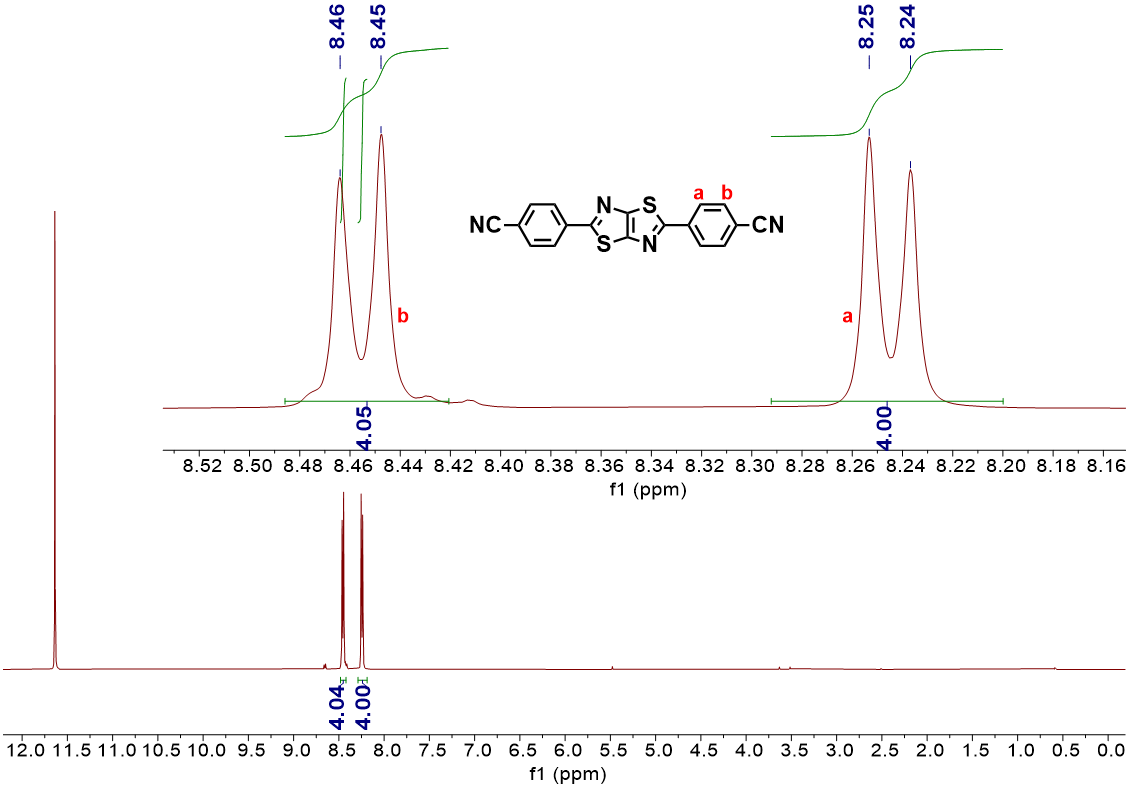
**

**Supplementary Figure S2.** ^1^H NMR spectra of TT–BN in CF_3_COOD.

**
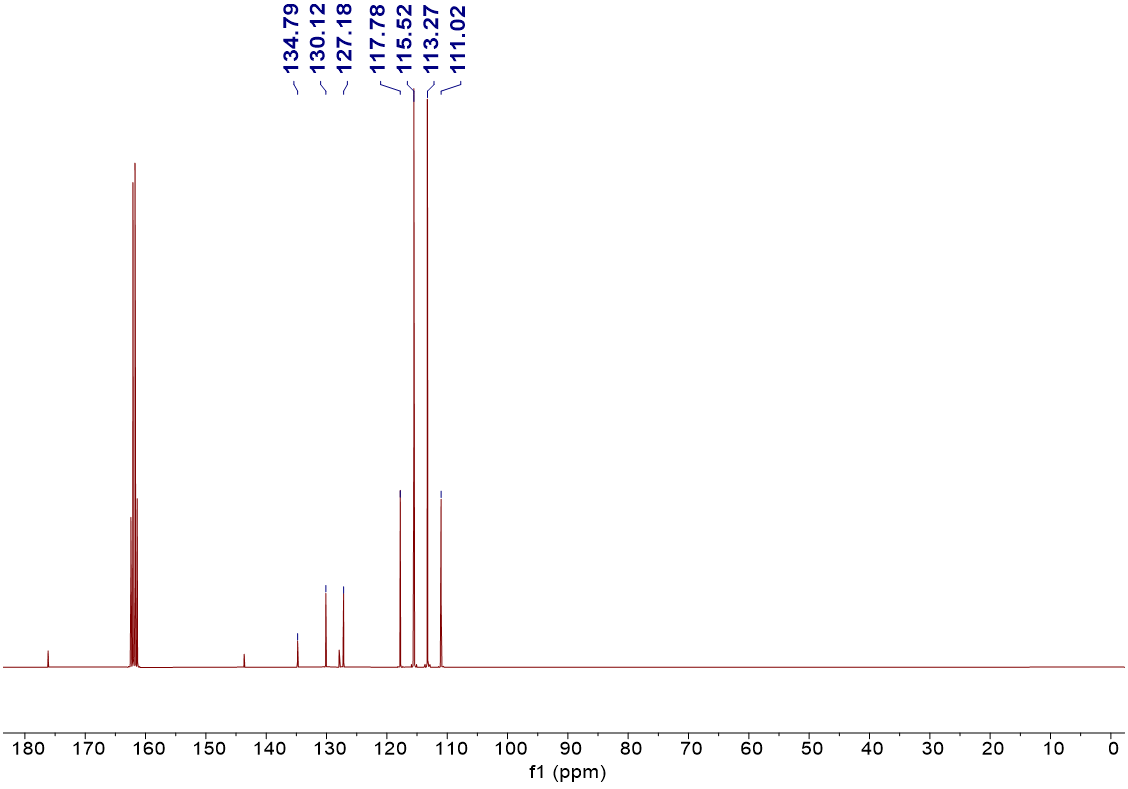
**

**Supplementary Figure S3.** ^13^C NMR spectra of TT–BN in CF_3_COOD.

**
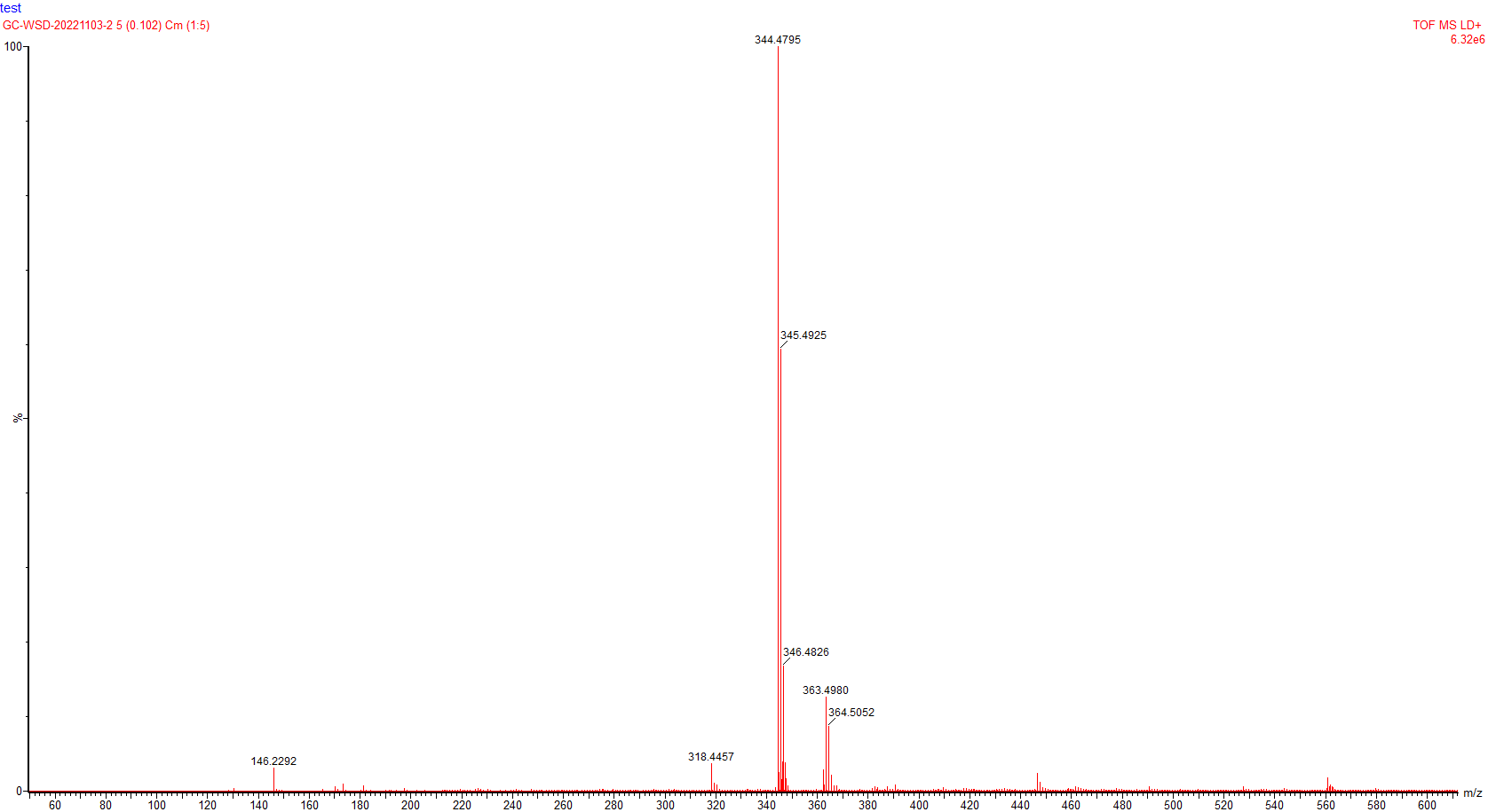
**

**Supplementary Figure S4.** ESI mass spectra of TT–BN.

**
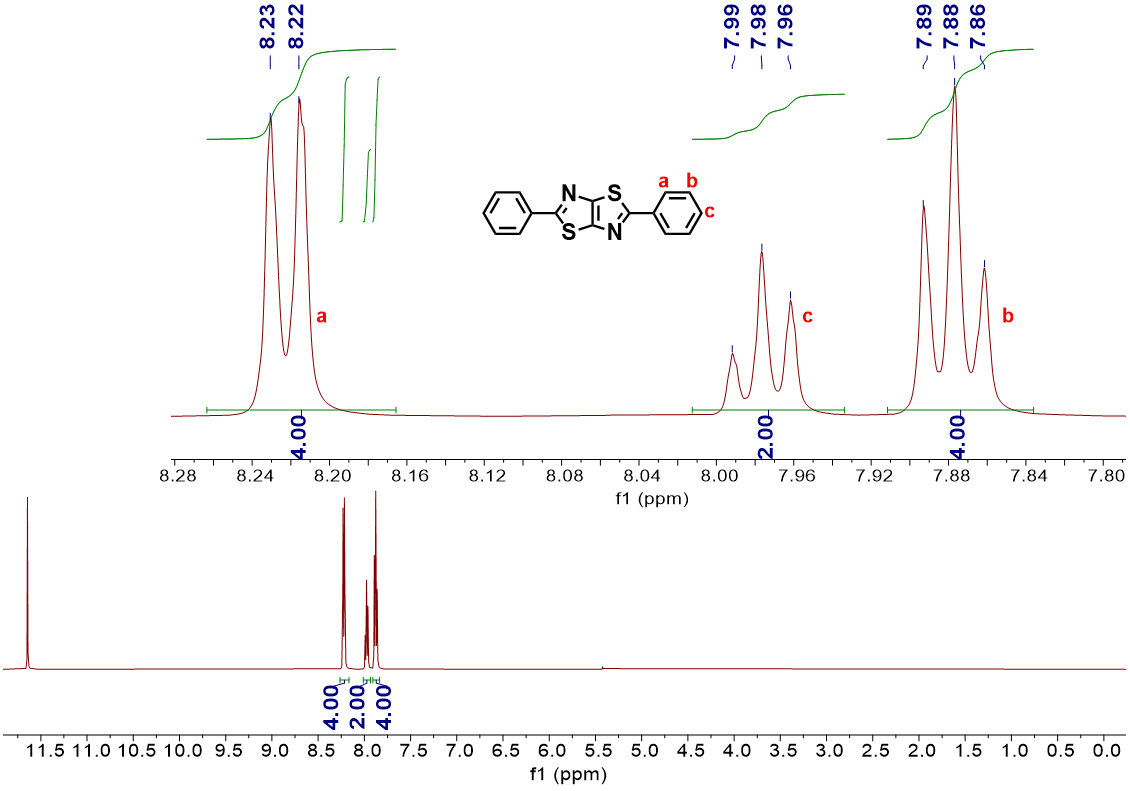
**

**Supplementary Figure S5.** ^1^H NMR spectra of TT–Bz in CF_3_COOD.

**
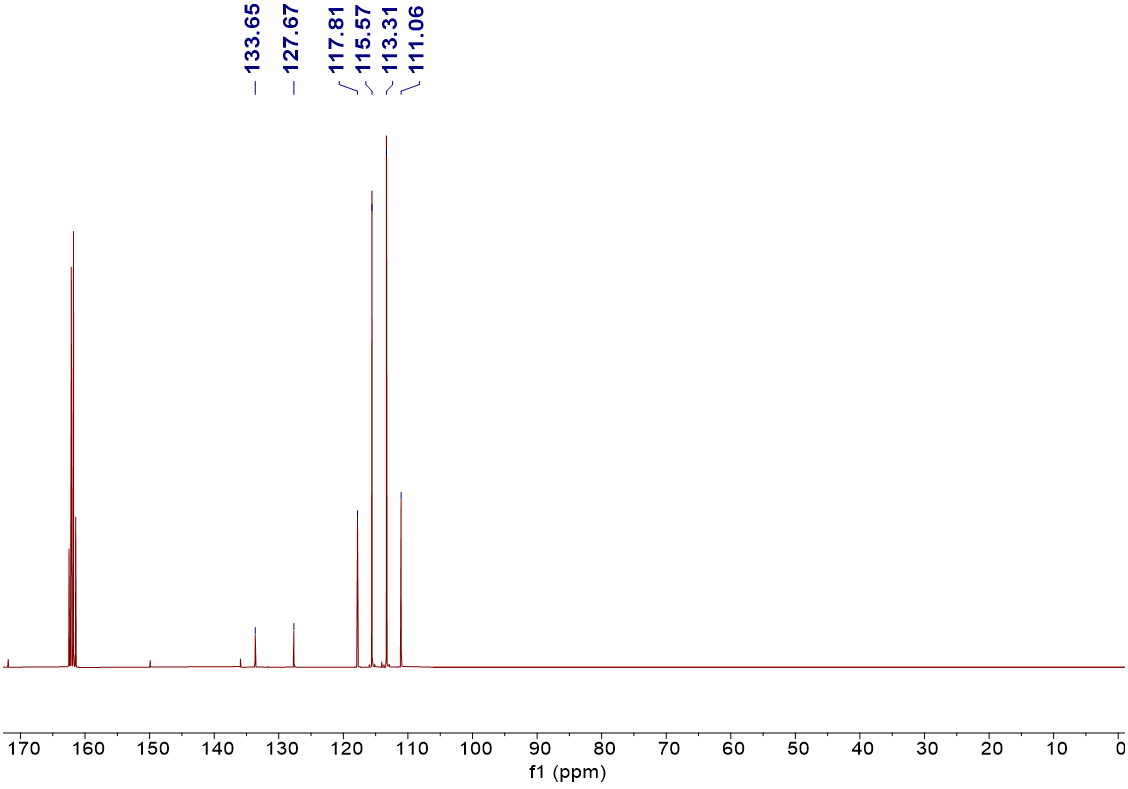
**

**Supplementary Figure S6.** ^13^C NMR spectra of TT–Bz in CF_3_COOD.

**
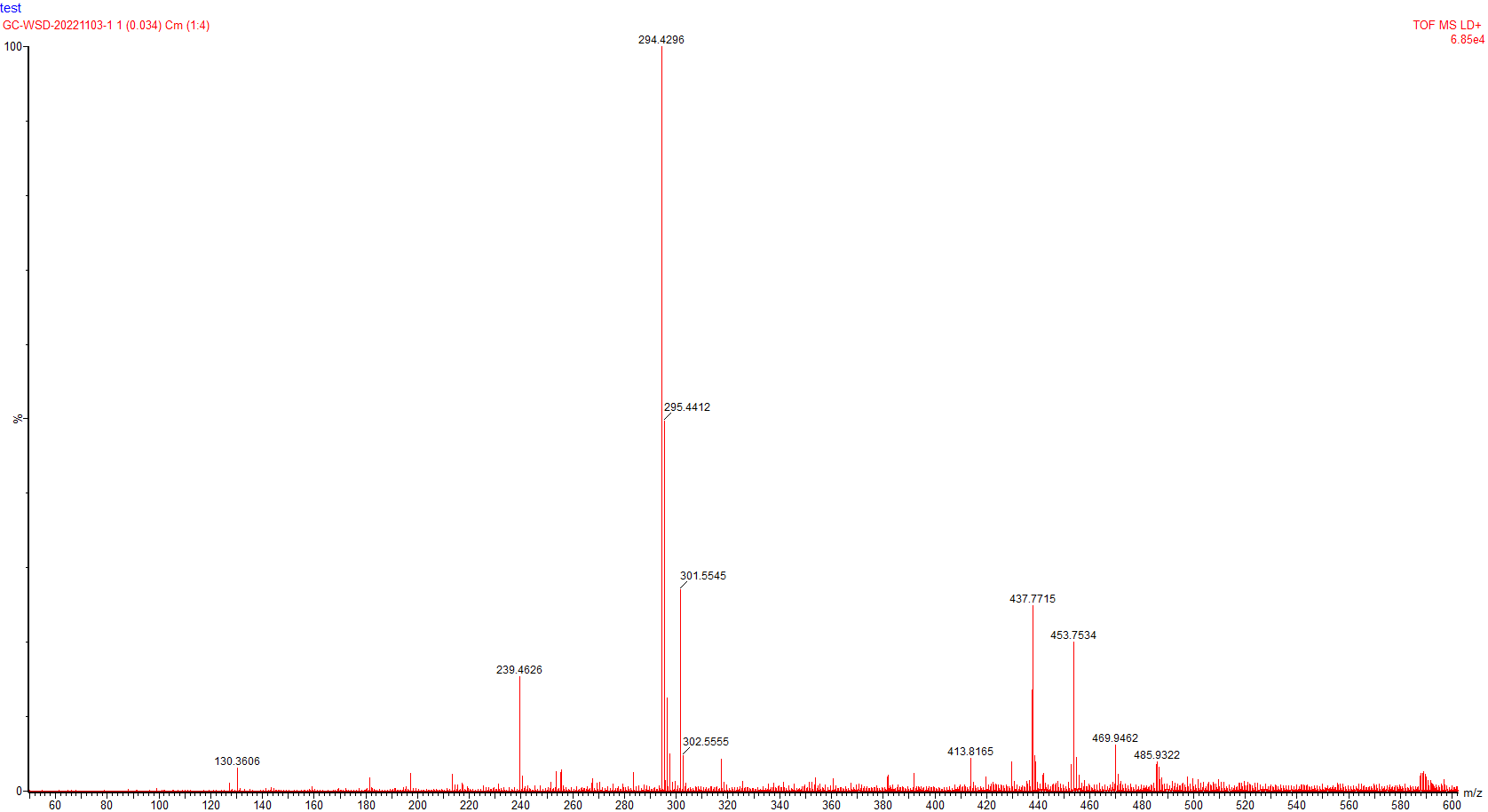
**

**Supplementary Figure S7.** ESI mass spectra of TT–Bz.

**Section 2: Structural analyses of the CTPs**

**
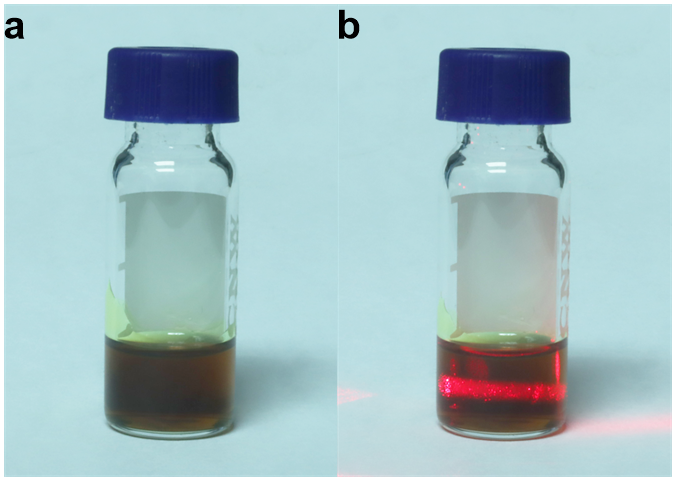
**

**Supplementary Figure S8.** Photo of TTH–CTP dispersed in triflic acid in the synthetic process. (a) Under natural light, (b) under laser irradiation showing the Tyndall effect.


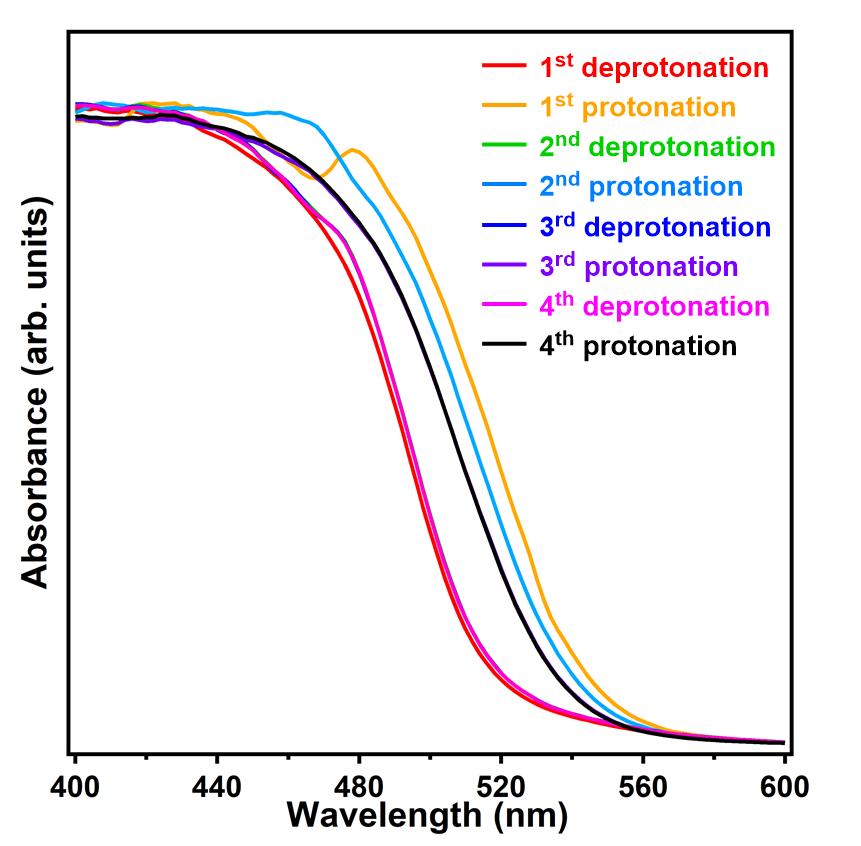


**Supplementary Figure S9.** UV-vis spectra of TT–CTP upon continuous addition of acid and base.


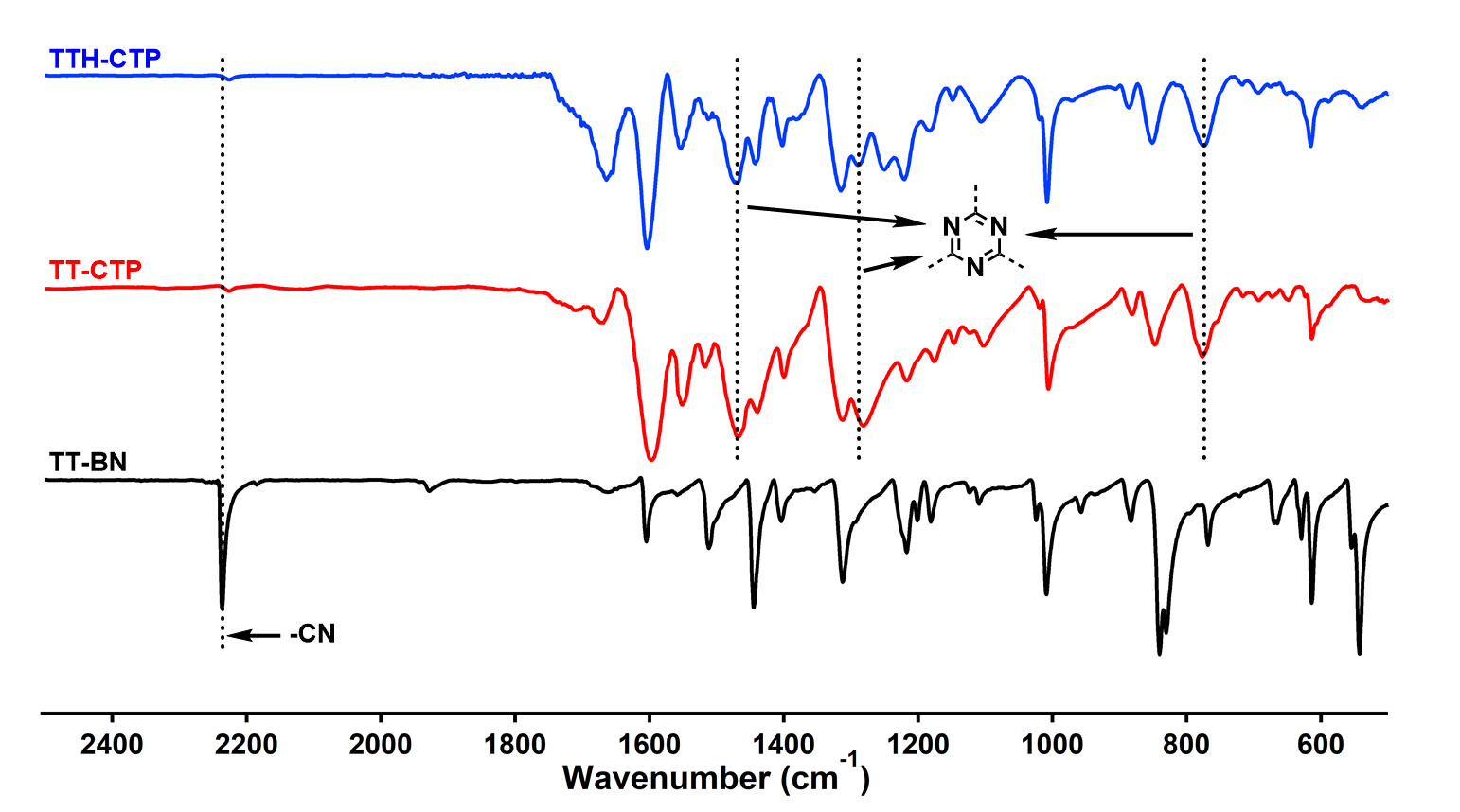


**Supplementary Figure S10.** Infrared spectra of the monomer and CTPs. The infrared spectra of TT–CTP and TTH–CTP showed three vibrational bands around 1466, 1289, and 776 cm^–1^, indicating the formation of the triazine ring; the cyano vibrational band at 2238 cm^–1^ in TT–BN monomer disappeared in TT–CTP and TTH–CTP, demonstrating the completeness of the reaction.

**
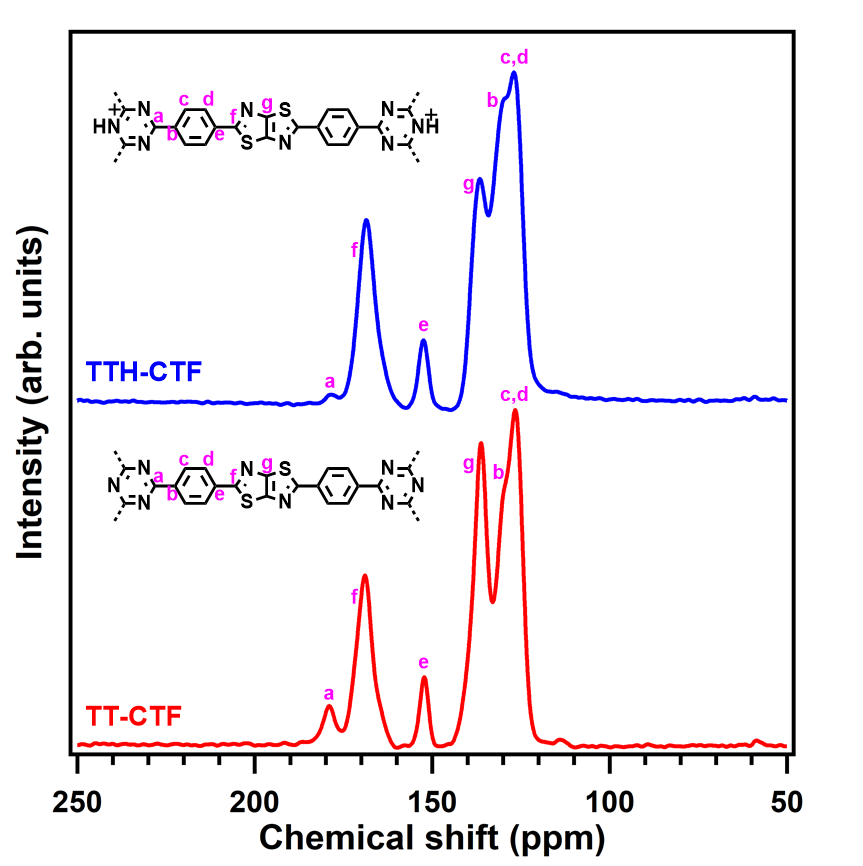
**

**Supplementary Figure S11.** Solid-state ^13^C NMR of TT–CTP and TTH–CTP.


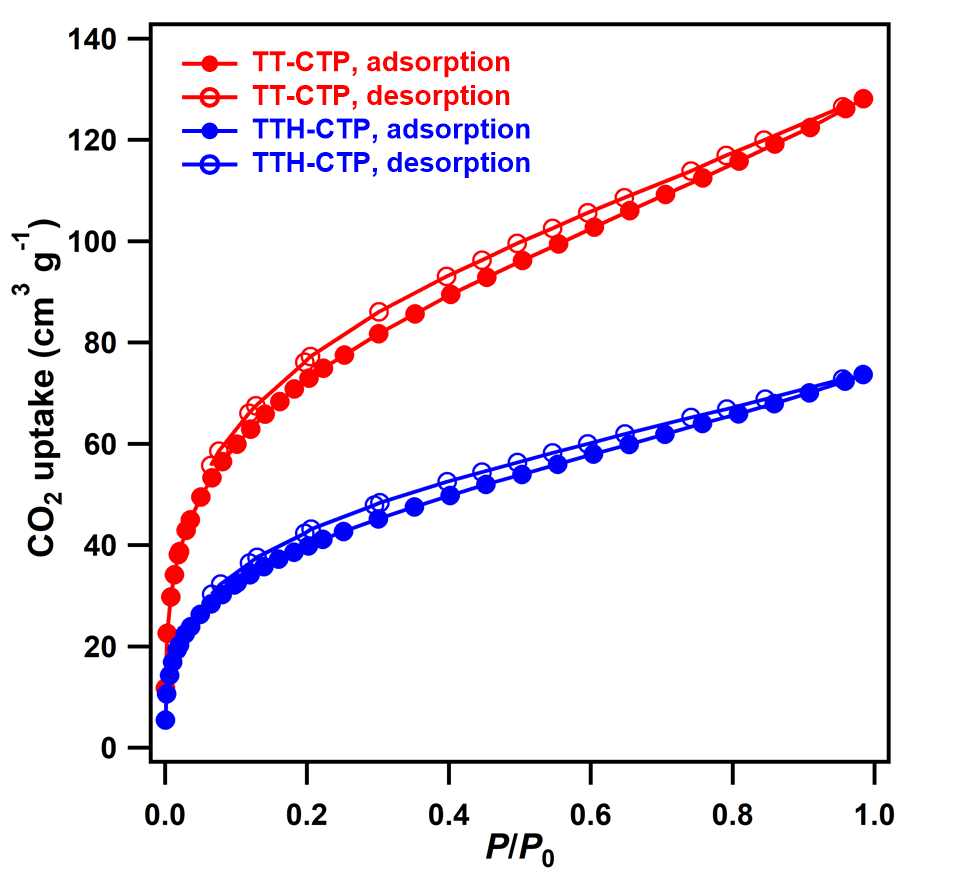


**Supplementary Figure S12.** CO_2_-sorption isotherms of TT–CTP and TTH–CTP at 195 K. Because of the small pores with high polarity, the two CTPs barely adsorbed nitrogen. The Brunauer-Emmett-Teller (BET) surface areas calculated from CO_2_-sorption isotherms were 387 and 268 m^2^ g^–1^ for TT–CTP and TTH–CTP, respectively.

**
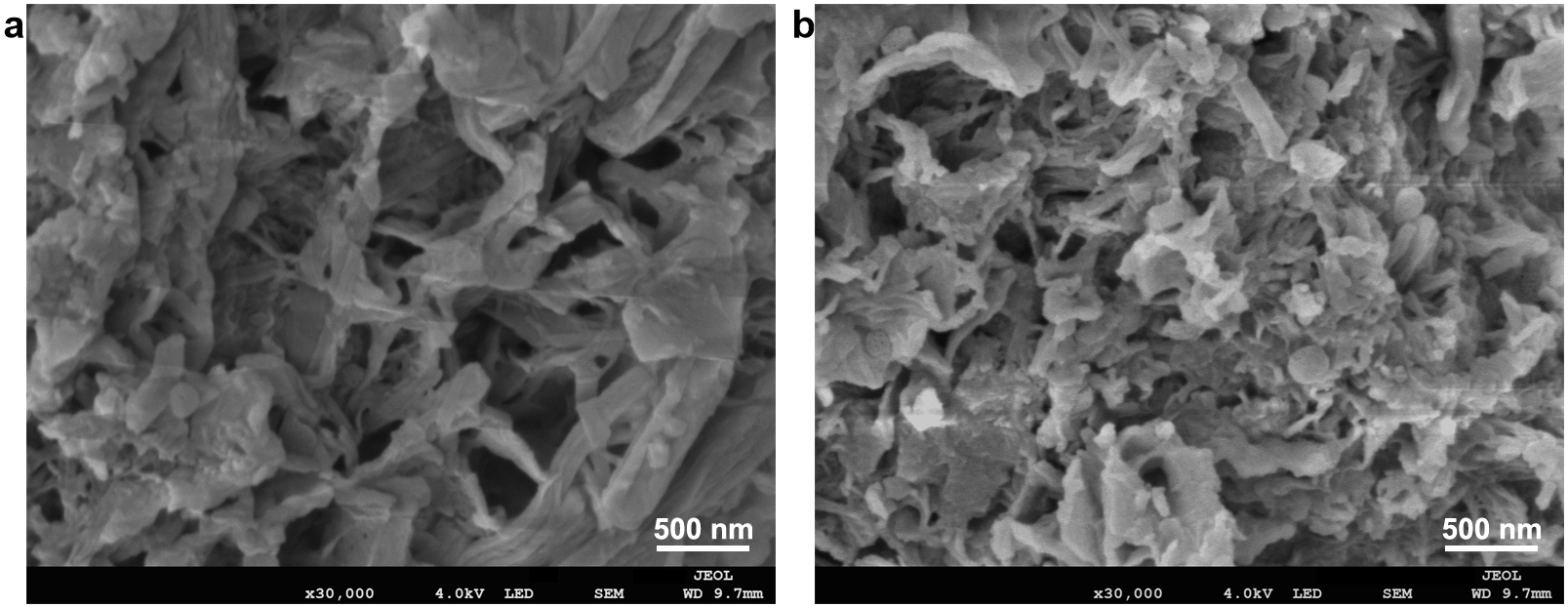
**

**Supplementary Figure S13.** SEM images of (a) TT–CTP and (b) TTH–CTP. The scale bars represent 500 nm.

**
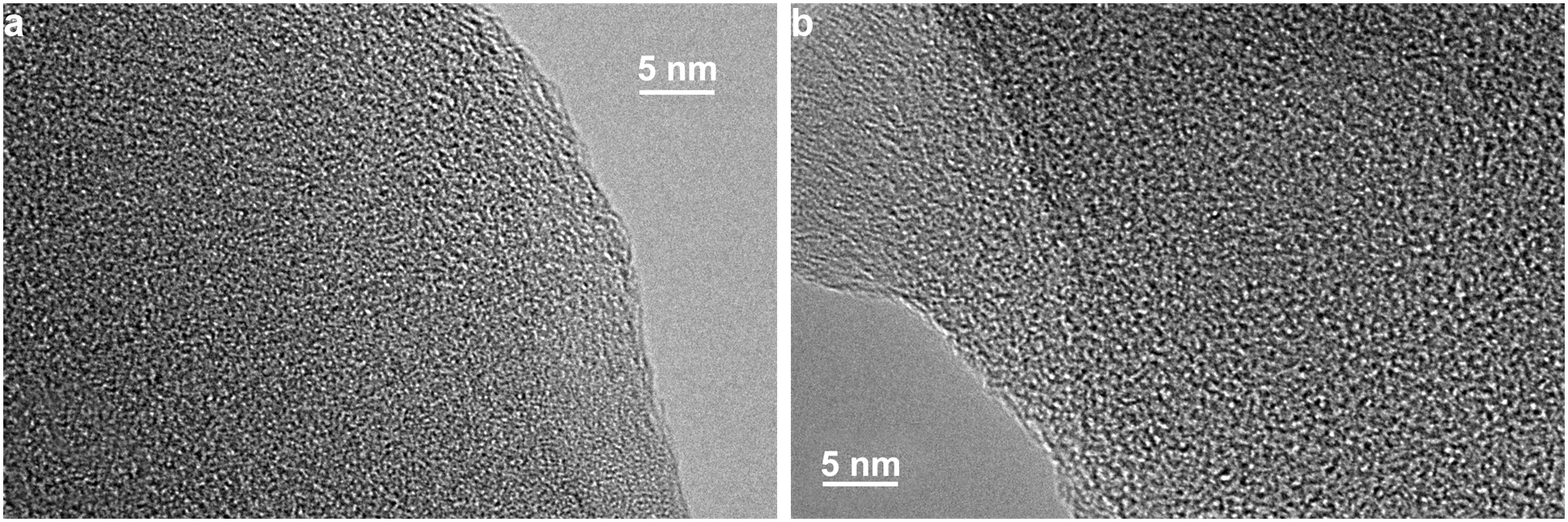
**

**Supplementary Figure S14.** TEM images of (a) TT–CTP and (b) TTH–CTP. The scale bars represent 5 nm.

**
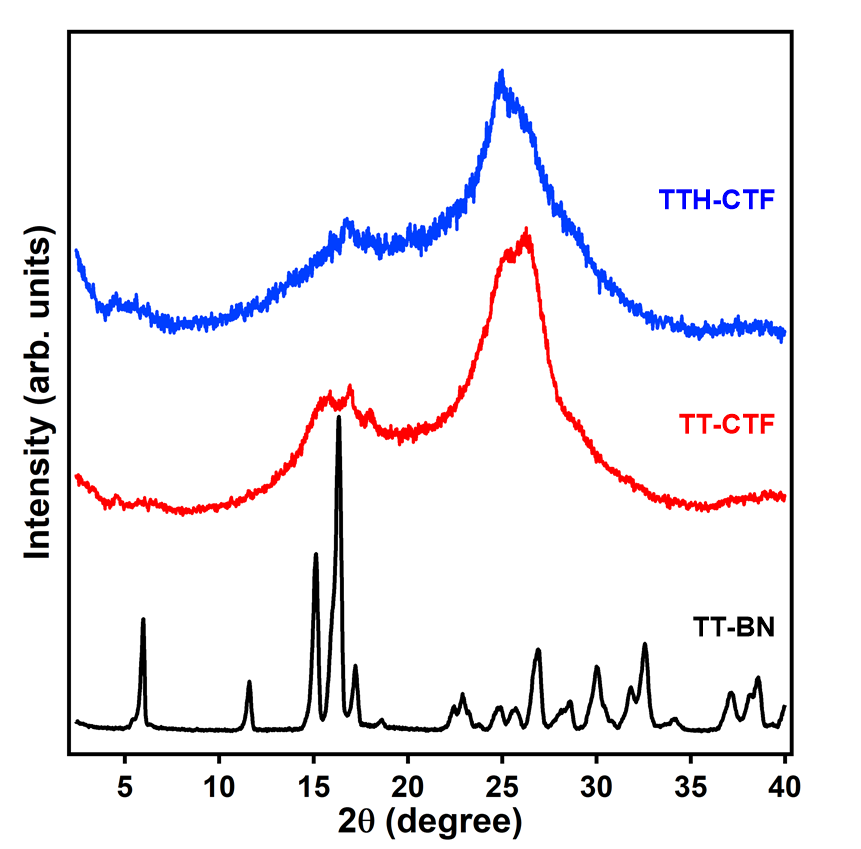
**

**Supplementary Figure S15.** PXRD patterns of TT–BN monomer, TT–CTP, and TTH–CTP.

**
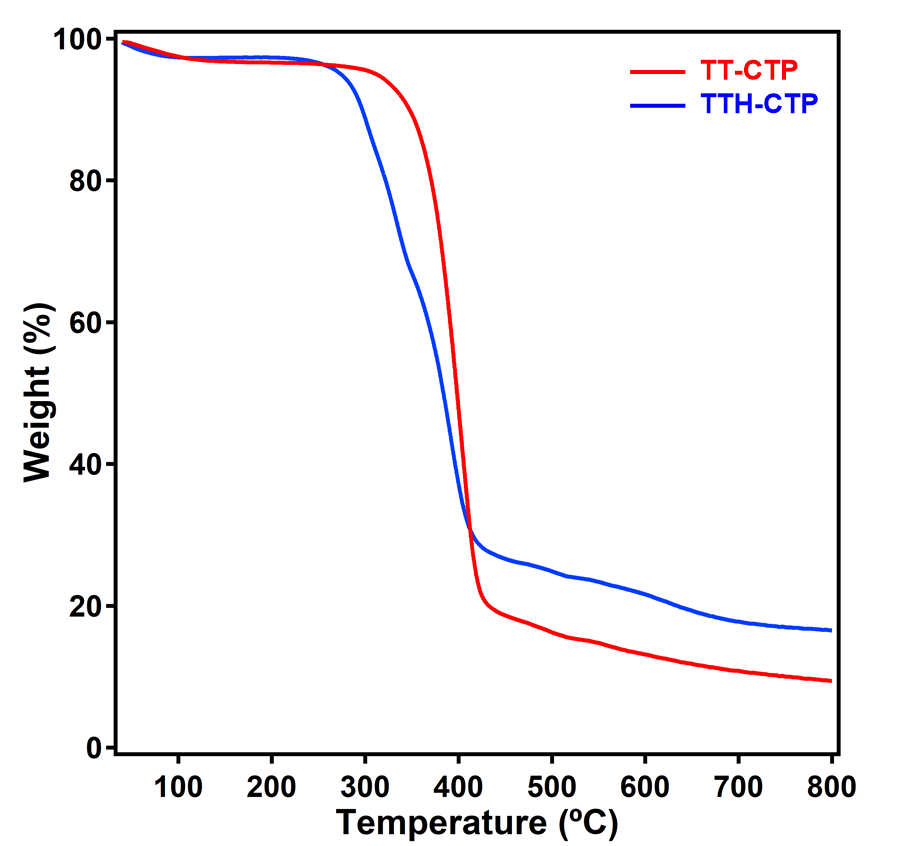
**

**Supplementary Figure S16.** TG curves of TT–CTP and TTH–CTP. TT–CTP and TTH–CTP were thermally stable until 340 and 278 °C, respectively.

**
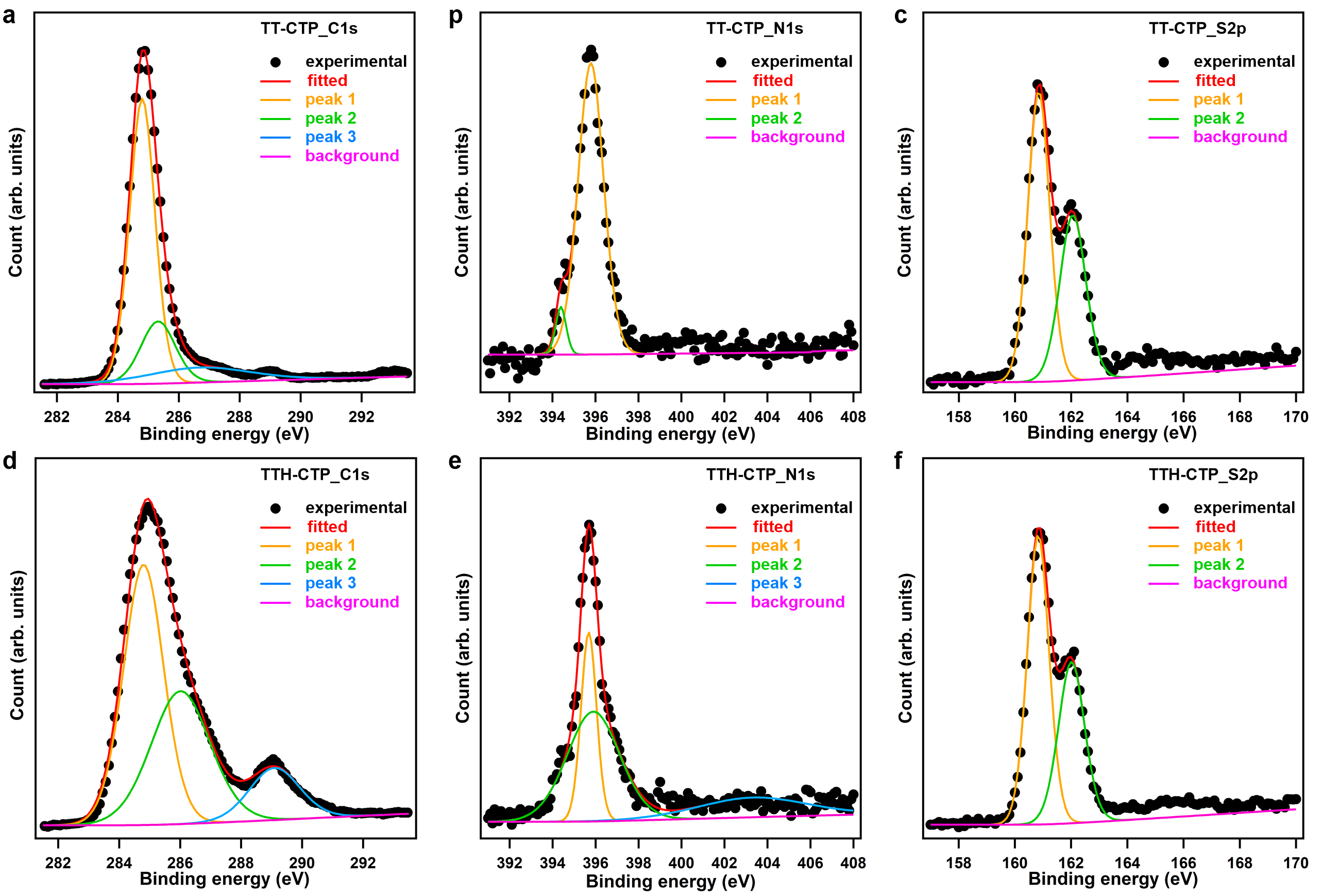
**

**Supplementary Figure S17.** XPS analysis of TT–CTP and TTH–CTP. (a) TT–CTP, C1s; (b) TT–CTP, N1s; (c) TT–CTP, S2p; (d) TTH–CTP, C1s; (e) TTH–CTP, N1s; (f) TTH–CTP, S2p.

In the N1s spectra of TT–CTP, the peak 2 attributing to the unreacted cyano group was only 6.2% of the total N atoms, demonstrating the successful formation of the triazine rings and the high polymerization degree. The N peaks attributing to the thiazole and triazine merged into one peak at 396 eV. On the other hand, in TTH–CTP, peaks 1 and 2 were attributed to the thiazole and triazine groups, respectively, whereas peak 3 was attributed to the protonated triazine group. The ratio of peaks 1, 2, and 3 was 1.4:2.2:1, which means that the pronated N atom was 31.3% of the total N atoms in one triazine ring. Therefore, the protonation degree was 93.8% (one triazine ring contains nearly one positive charge). Additionally, the S2p spectra remained almost unchanged before and after protonation, which further indicated that the protonation occurred on the triazine rather than thiazole.

**Section 3: Solution processing of the CTPs**

**
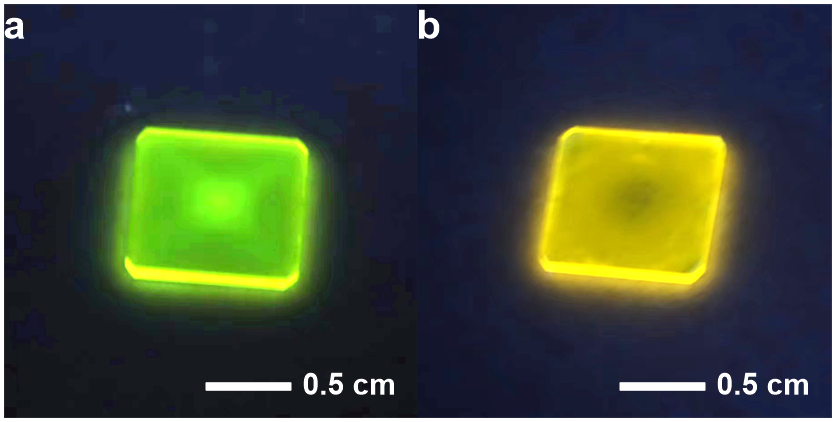
**

**Supplementary Figure S18.** Photos of spin-coated thin films of (a) TT–CTP and (b) TTH–CTP under UV light.


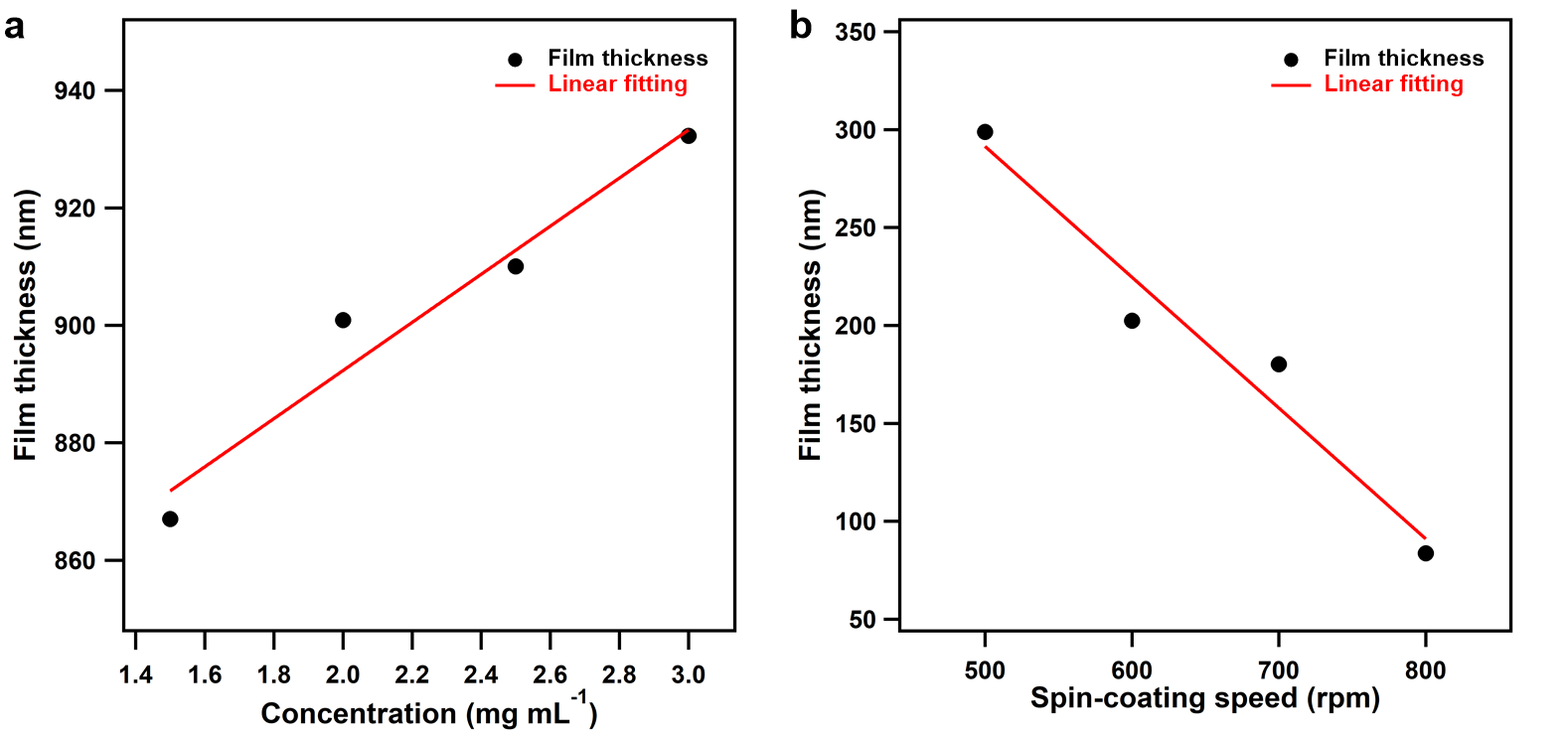


**Supplementary Figure S19.** Dependency of film thickness with concentration and spin-coating speed. (a) Correlation of the film thickness with the concentration of TTH–CTP (500 rpm). (b) Correlation of the film thickness with the spin-coating speed (1 mg mL^−1^).

**Section 4: Basic photophysical properties of the CTPs**

**
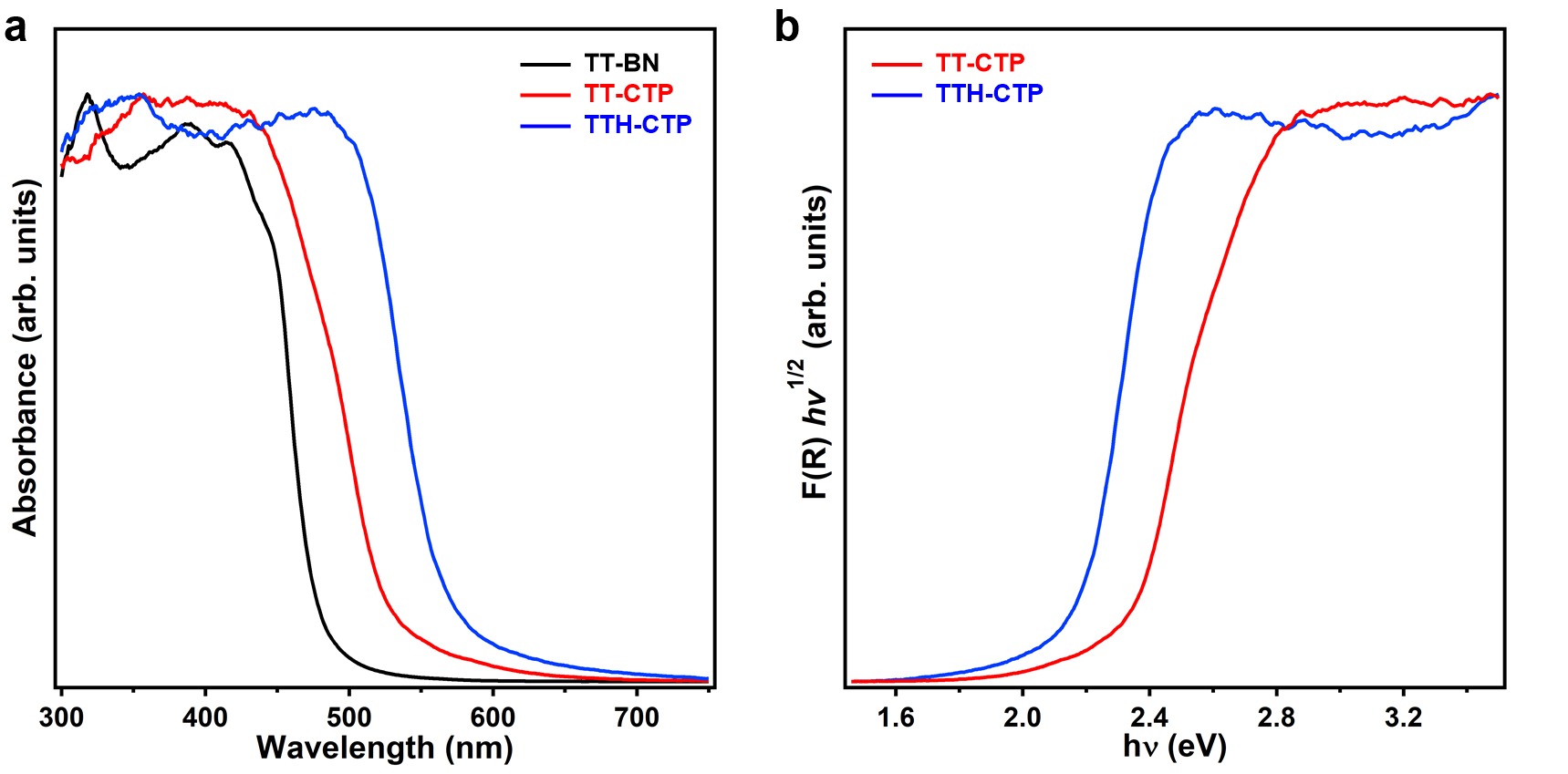
**

**Supplementary Figure S20.** Absorption property of the monomer and CTPs. (a) UV-vis spectra of TT–BN, TT–CTP, and TTH–CTP. (b) Tauc plot of TT–CTP and TTH–CTP.

**
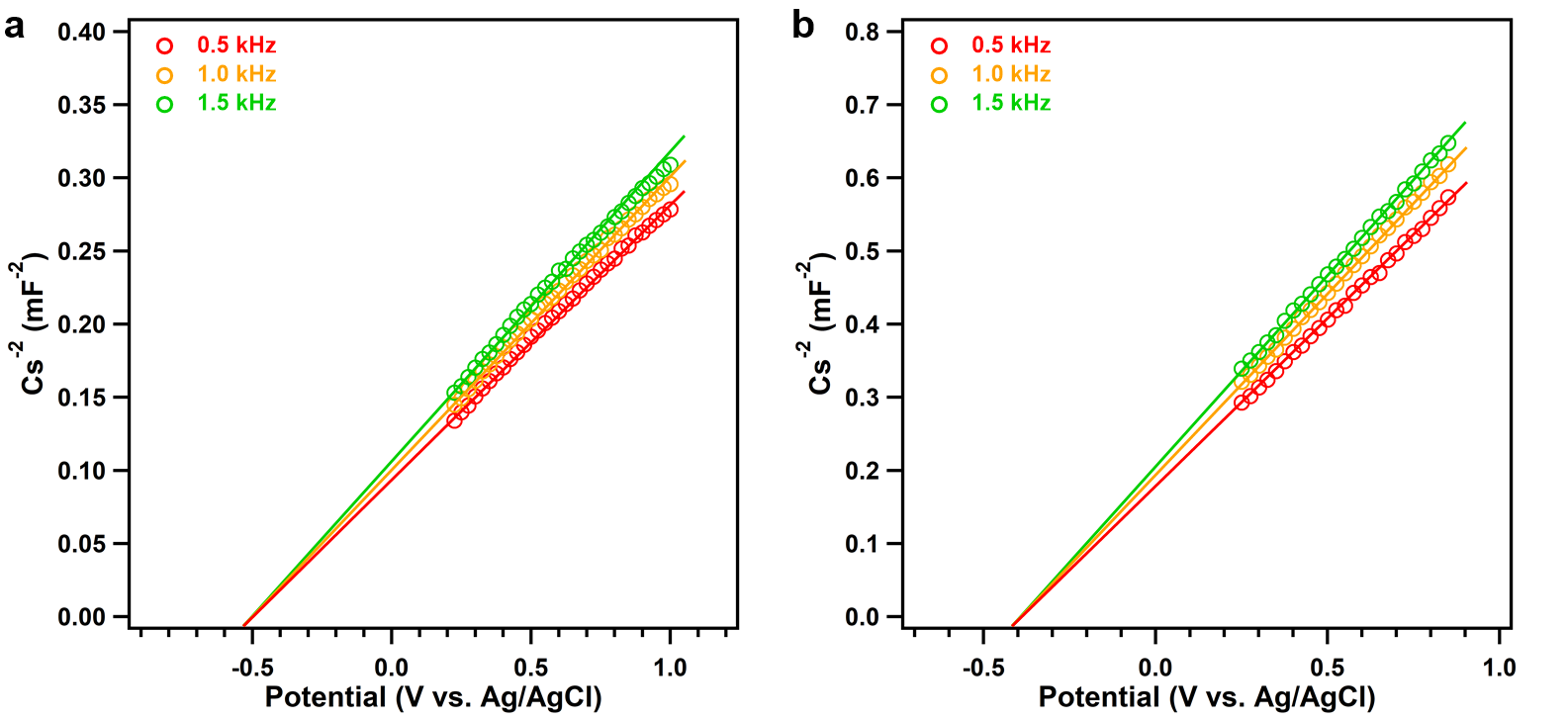
**

**Supplementary Figure S21.** Mott-Schottky plots of (a) TT–CTP and (b) TTH–CTP.

**
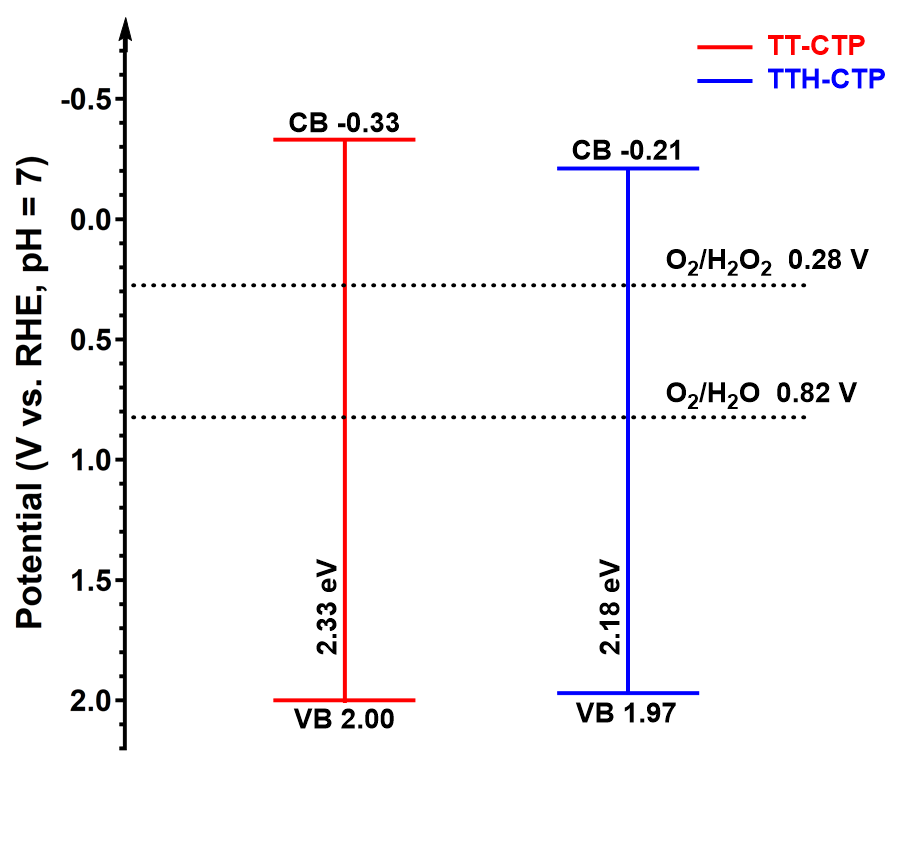
**

**Supplementary Figure S22.** Band structures of TT–CTP and TTH–CTP.

**
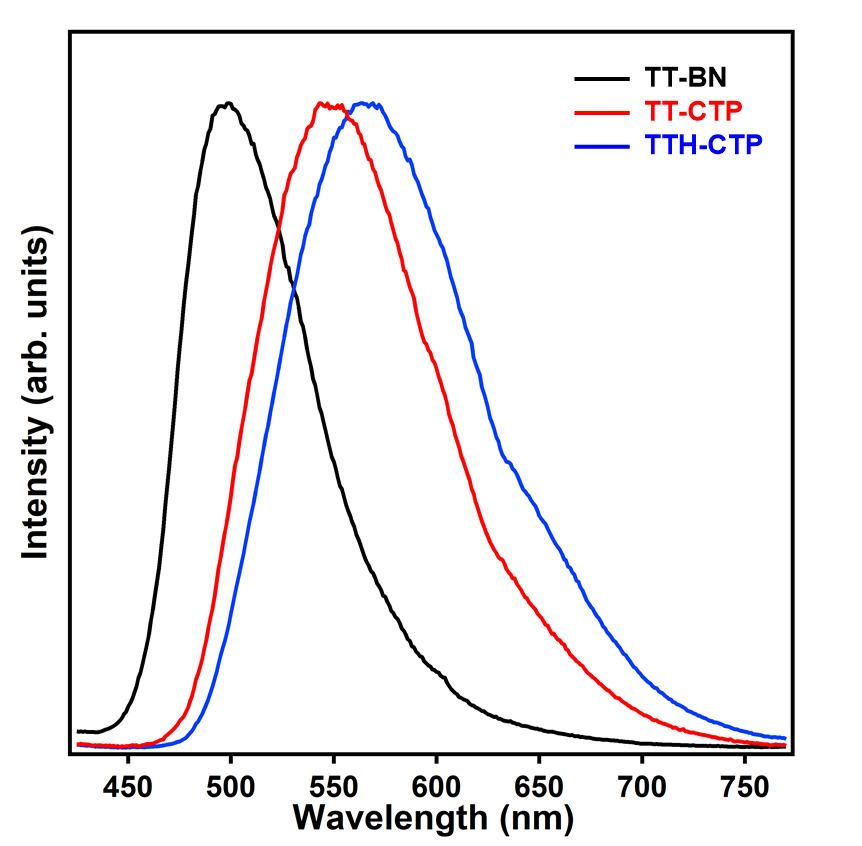
**

**Supplementary Figure S23.** Photoluminescence spectra of TT–BN, TT–CTP, and TTH–CTP in the air.

**
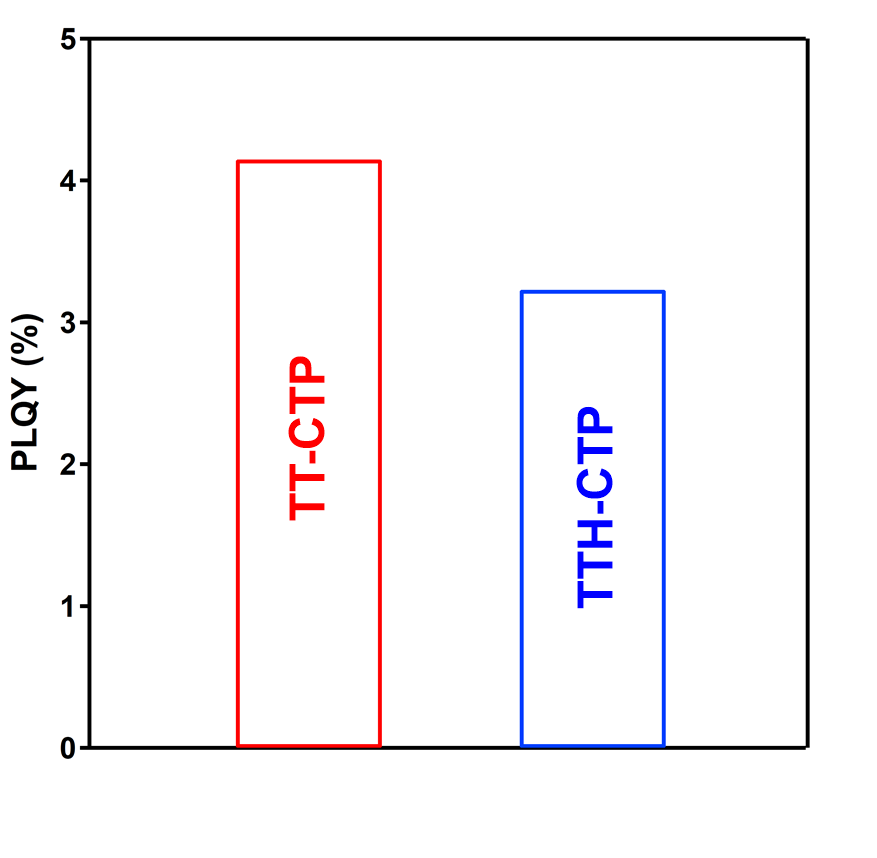
**

**Supplementary Figure S24.** Photoluminescence quantum yields of TT–CTP and TTH–CTP in the air.


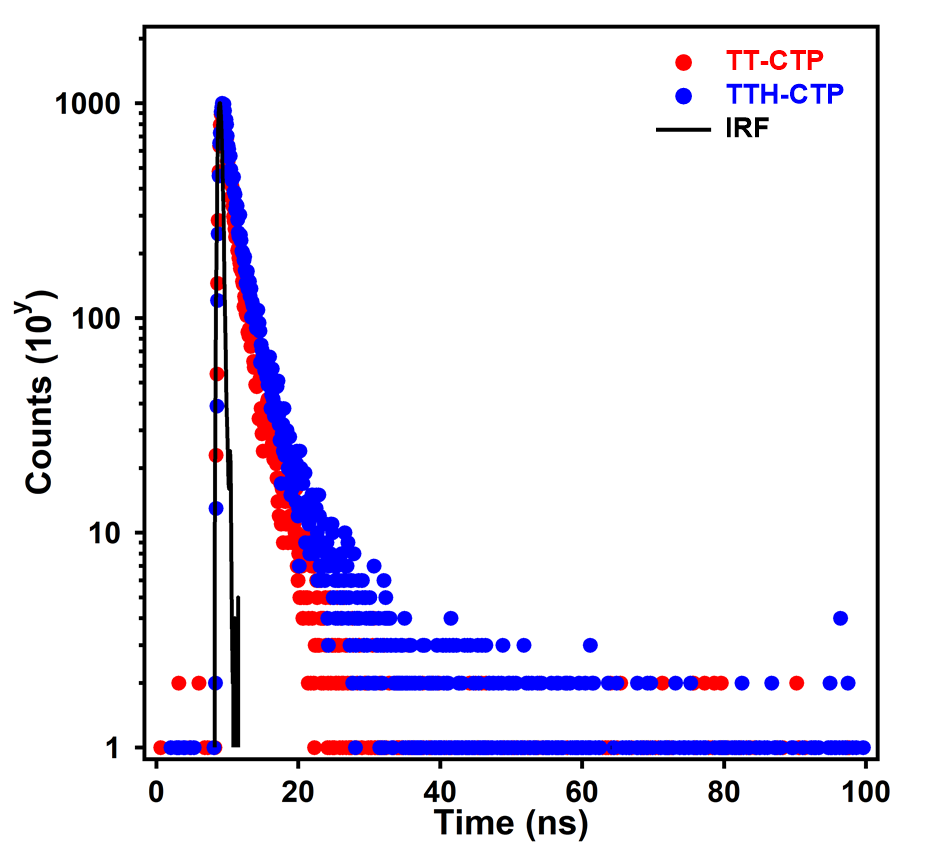


**Supplementary Figure S25.** Photoluminescence decay profiles of TT–CTP and TTH–CTP in the air.


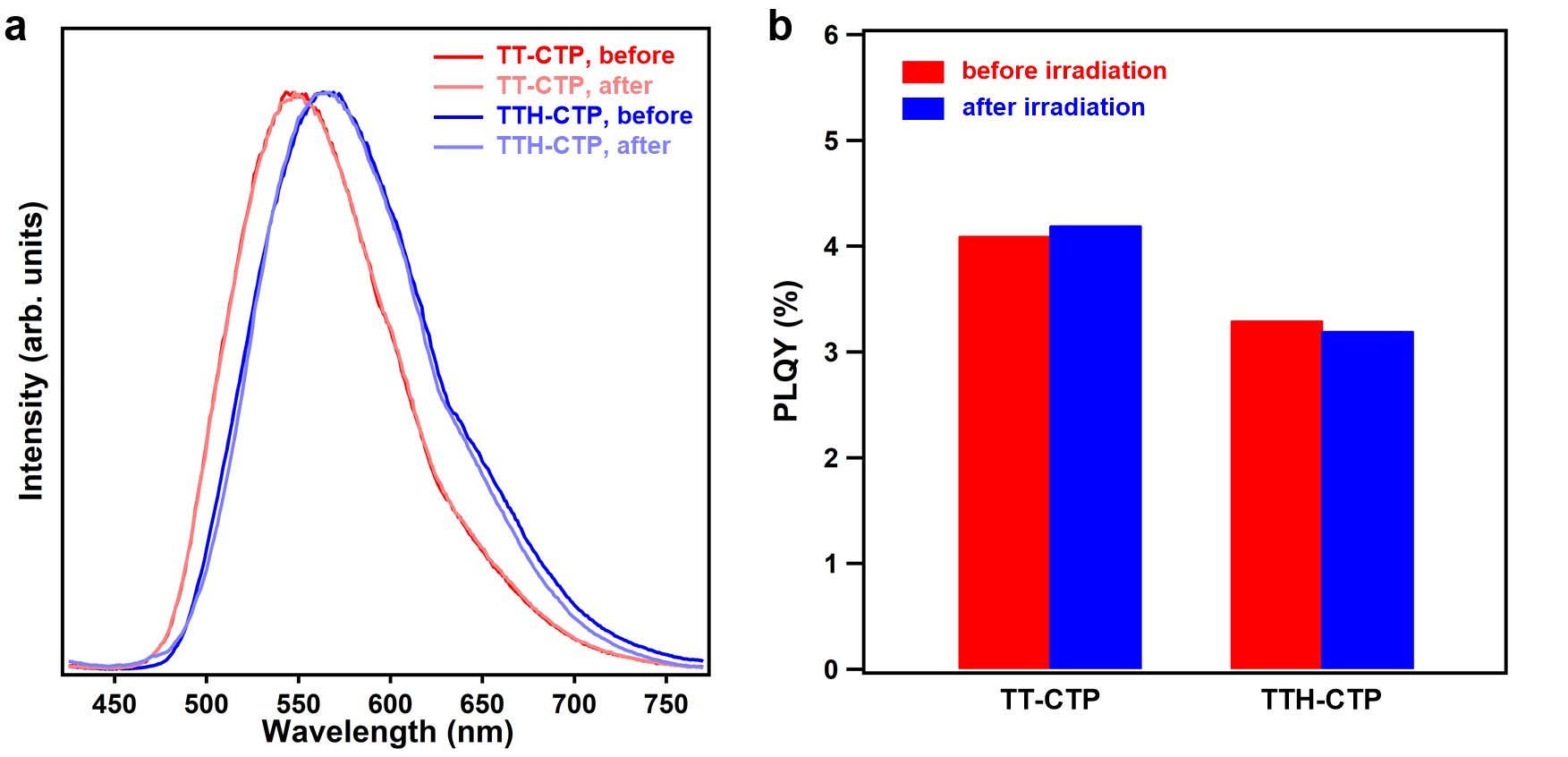


**Supplementary Figure S26.** Photostability of the CTPs. (a) Photoluminescence spectra and (b) PLQY of TT–CTP and TTH–CTP before and after irradiation with a white-light Xe lamp (300 W) for 3 h in the air.


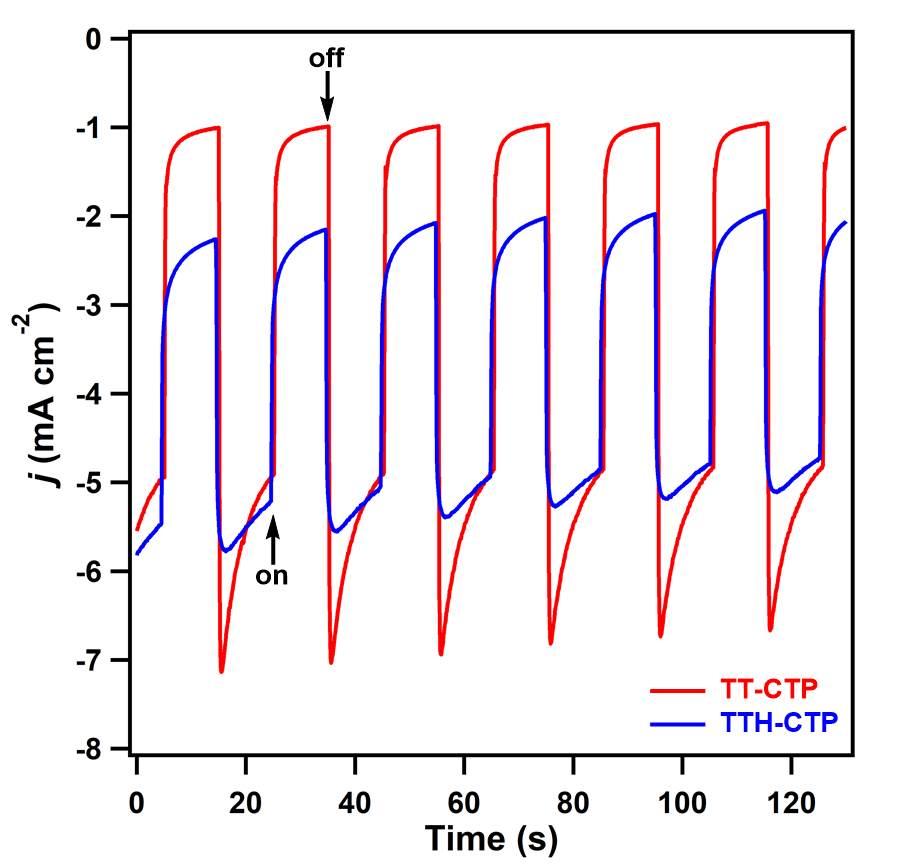


**Supplementary Figure S27.** Transient photocurrent response of TT–CTP and TTH–CTP.


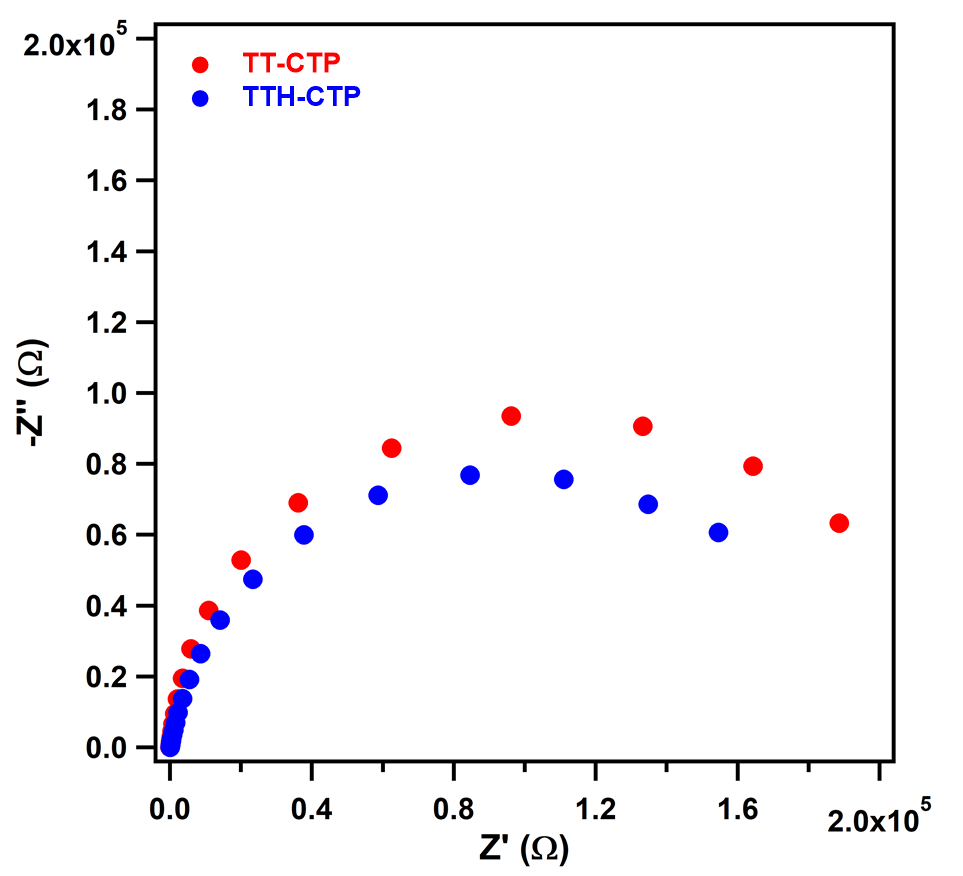


**Supplementary Figure S28.** Electrochemical impedance spectra of TT–CTP and TTH–CTP.

**Section 5: Photocatalytic production of H_2_O_2_**

**
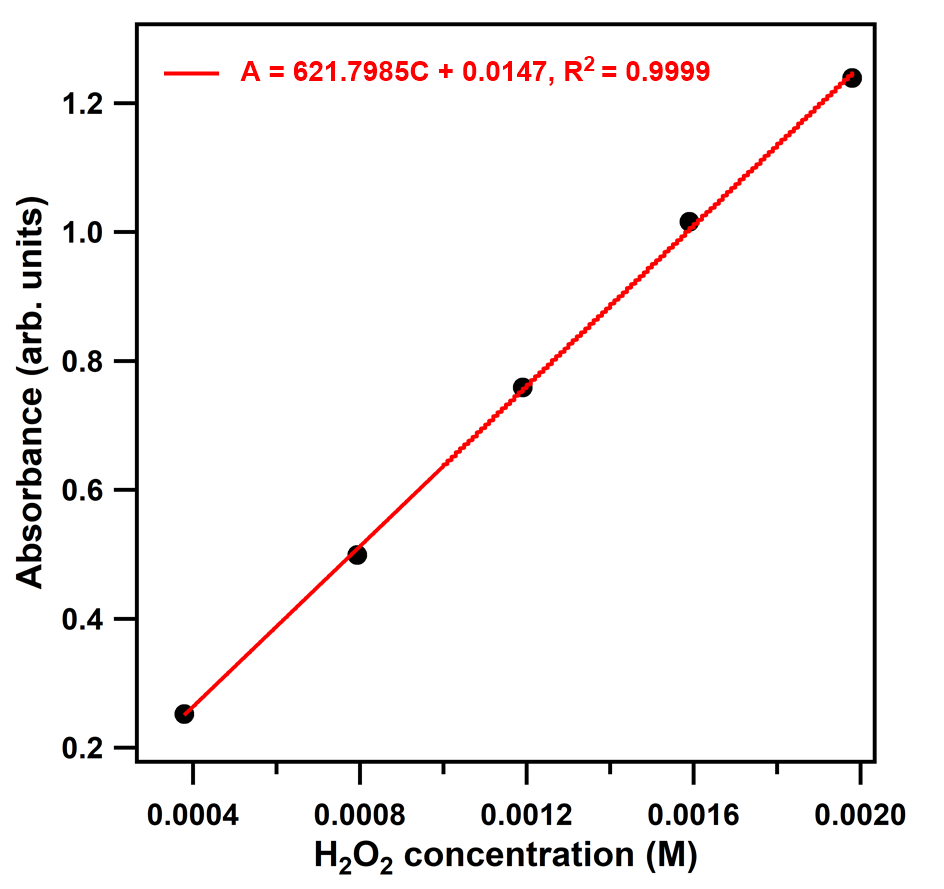
**

**Supplementary Figure S29.** Working curve of the titanium sulfate spectrophotometry. Based on the stable orange-yellow complex (410 nm maximum absorption wavelength) formed by H_2_O_2_ with Ti^4+^ ion, the content of H_2_O_2_ was analyzed by titanium sulfate spectrophotometry. After the reaction, the reaction liquid is collected by filtration. The reaction liquid (5 mL) to be measured was added to 2 mL of the prepared titanium sulfate Ti(SO_4_)_2_ solution and then transferred to a 25 mL volumetric bottle for constant volume. The absorbance of part of the solution was measured by UV spectrophotometer.

**
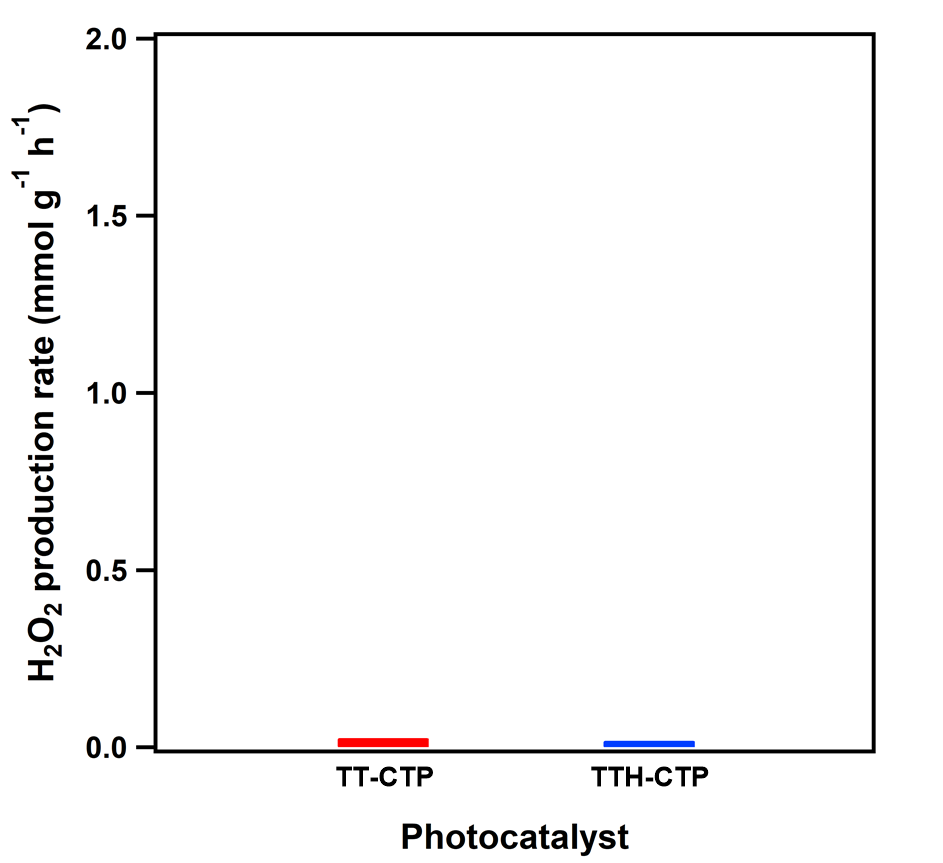
**

**Supplementary Figure S30.** H_2_O_2_ generation rates of photocatalytic half reaction without TT–CTP or TTH–CTP as the photocatalyst.


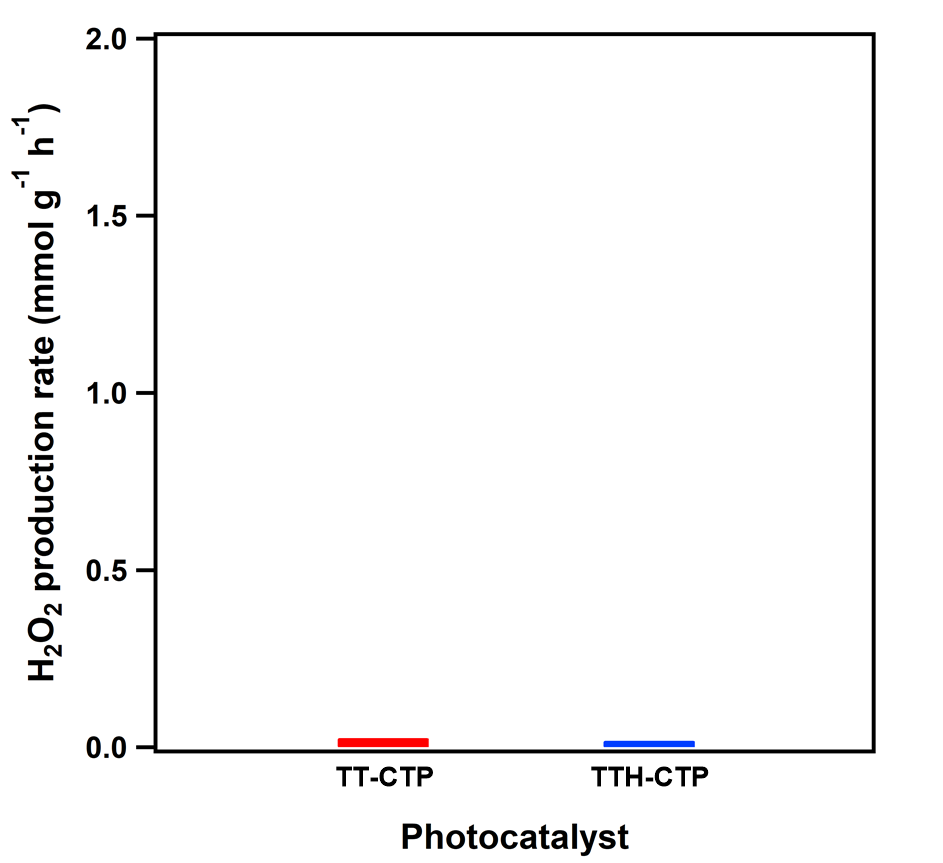


**Supplementary Figure S31.** H_2_O_2_ generation rates of photocatalytic half reaction with TT–CTP or TTH–CTP as the photocatalyst without light irradiation.

**
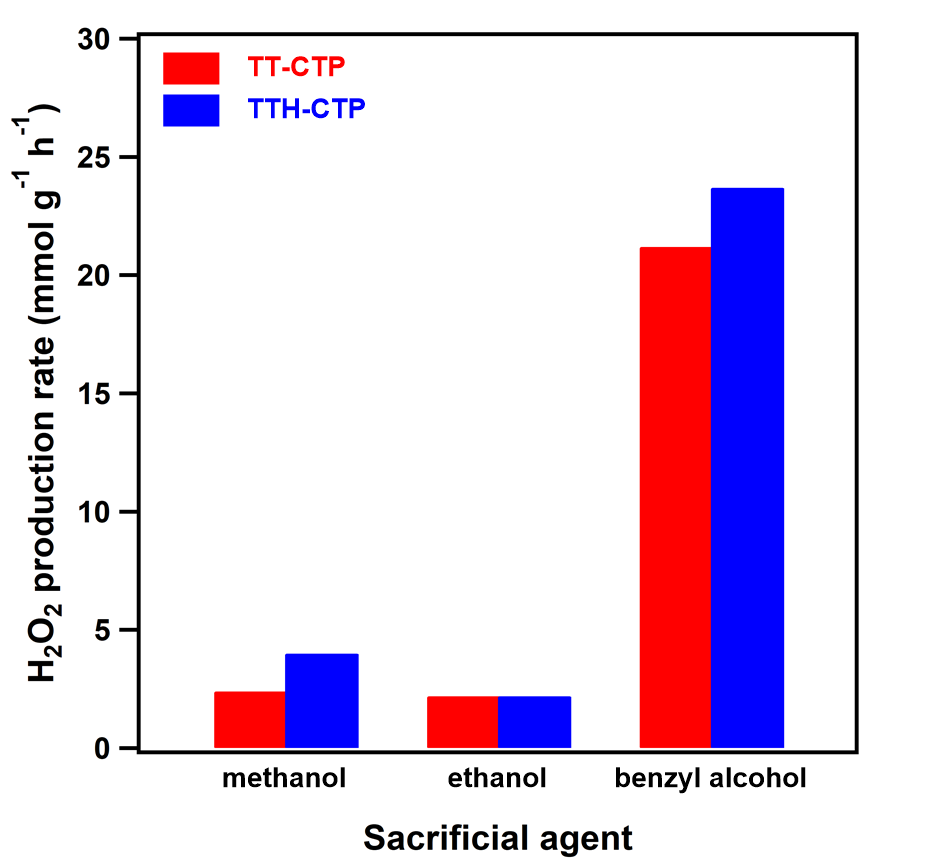
**

**Supplementary Figure S32.** H_2_O_2_ generation rates of photocatalytic half reaction of TT–CTP and TTH–CTP with different hole sacrificial agents.

**
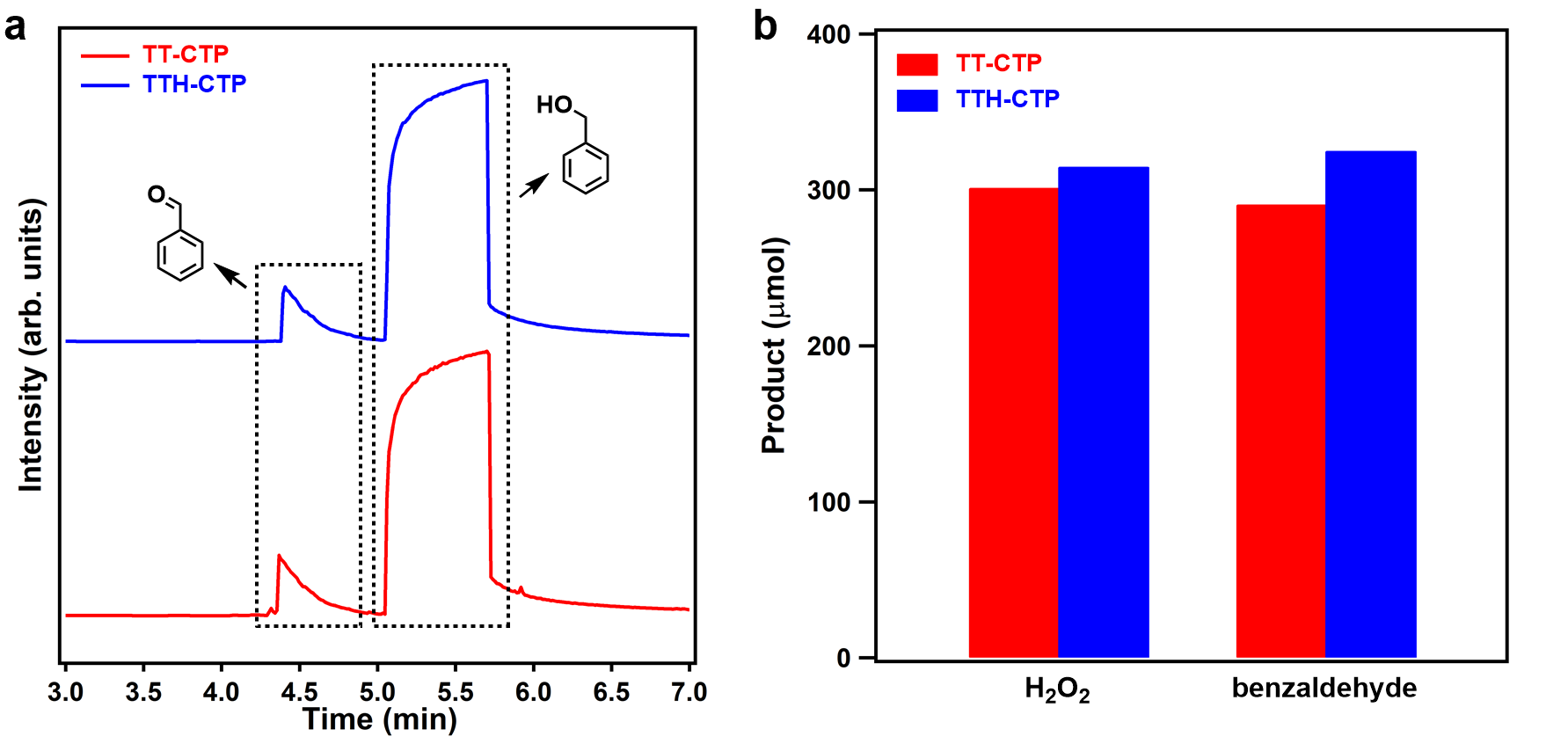
**

**Supplementary Figure S33.** Detection of the produced benzaldehyde. (a) Gas chromatography data of the produced benzaldehyde. (b) Concentrations of H_2_O_2_ and benzaldehyde in the photocatalytic full reaction of TT–CTP and TTH–CTP.


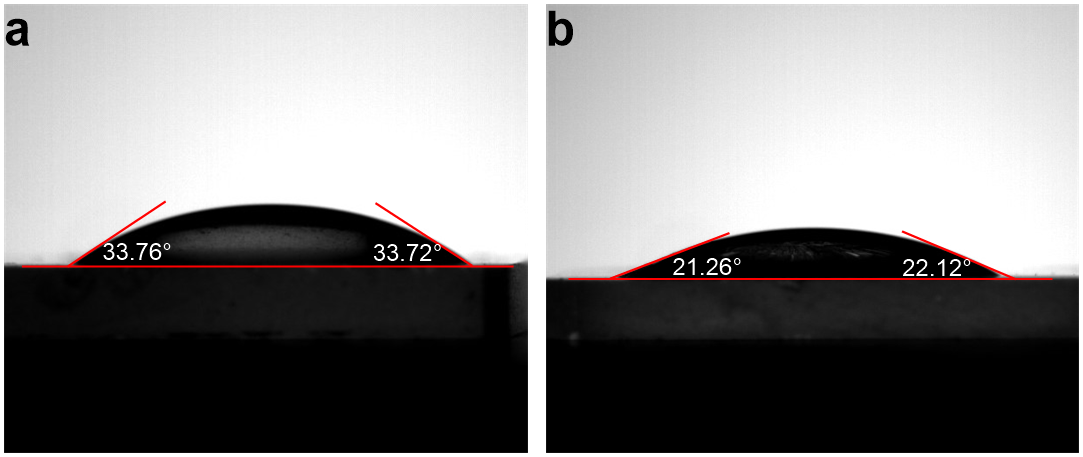


**Supplementary Figure S34.** Contact-angle measurements for (a) TT–CTP and (b) TTH–CTP thin films.

**
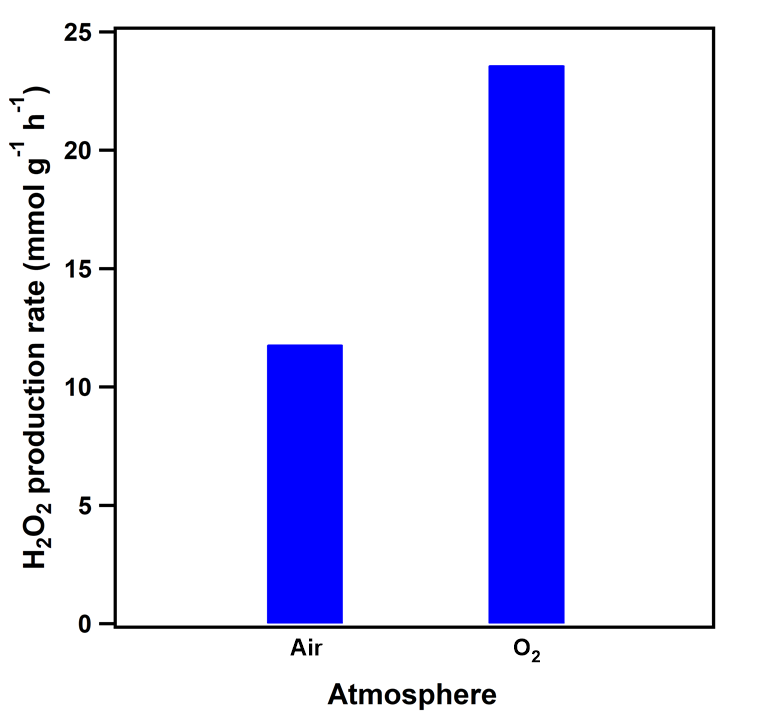
**

**Supplementary Figure S35.** H_2_O_2_ generation rates of photocatalytic half reaction of TTH–CTP in air and O_2_ conditions.

**
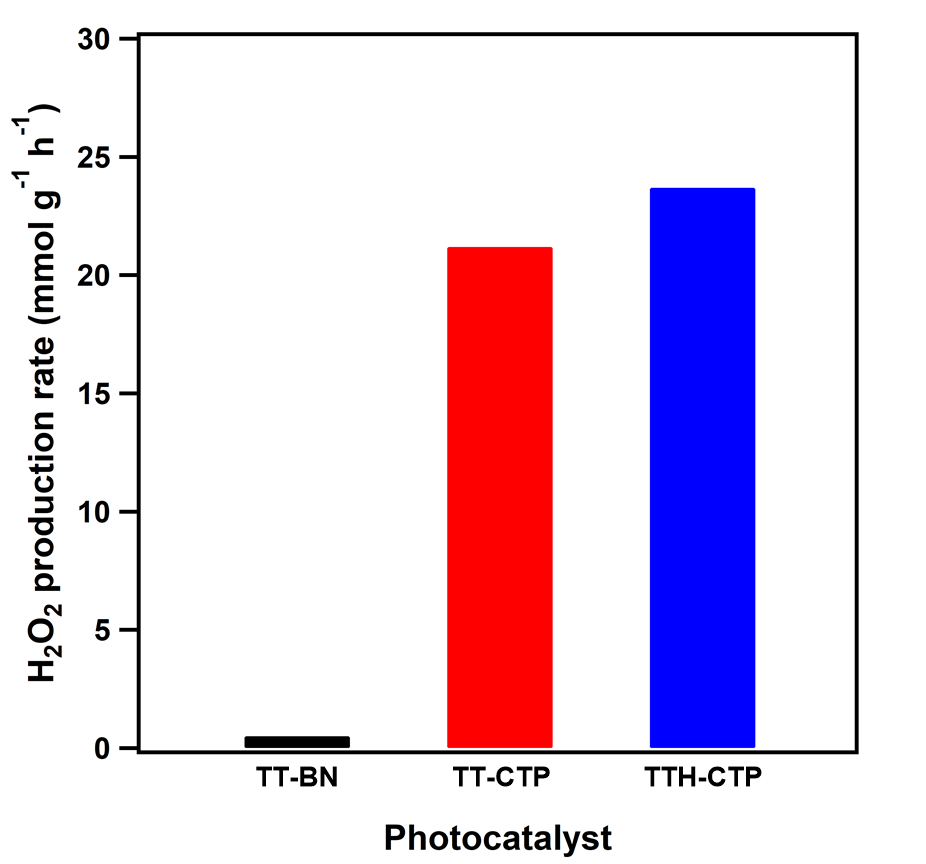
**

**Supplementary Figure S36.** H_2_O_2_ generation rates of photocatalytic half reaction with TT–BN, TT–CTP, and TTH–CTP as the photocatalyst.

**
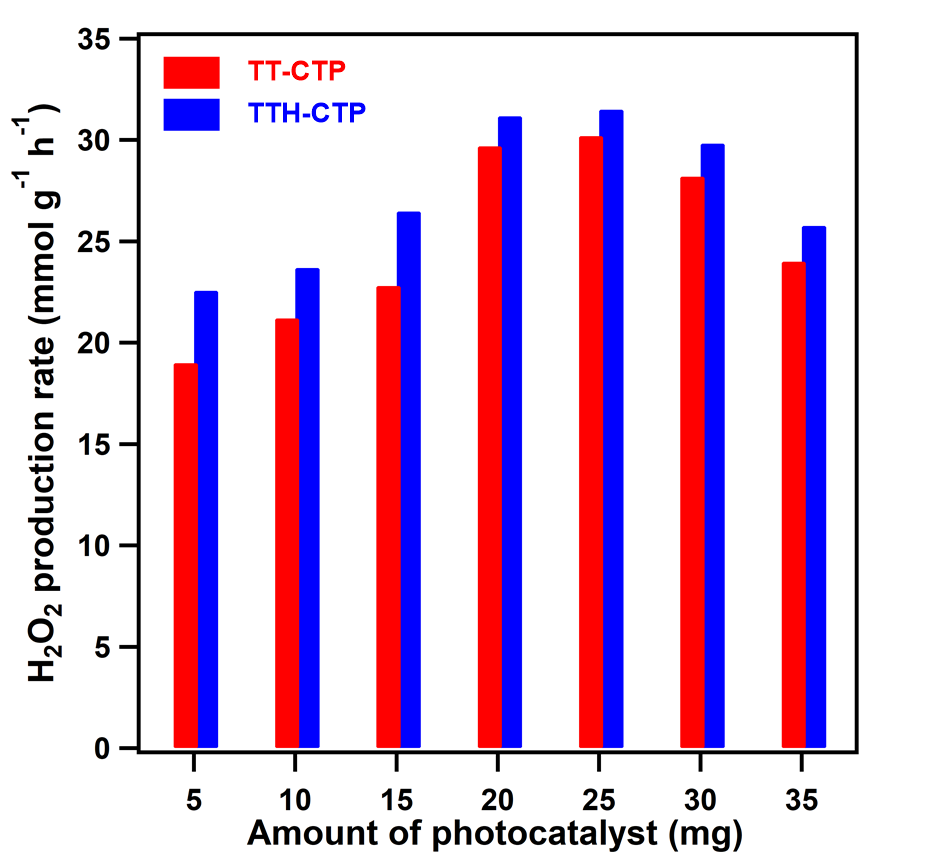
**

**Supplementary Figure S37.** H_2_O_2_ generation rates of photocatalytic half reaction of TT–CTP and TTH–CTP under different photocatalyst concentrations.

**
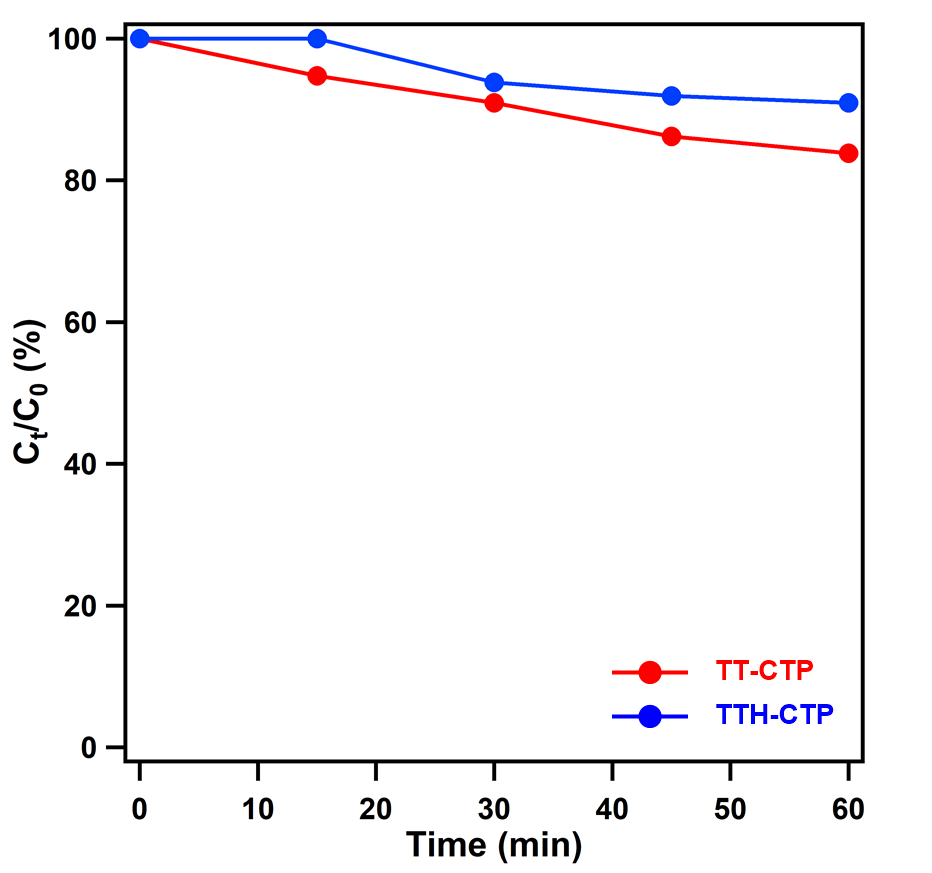
**

**Supplementary Figure S38.** H_2_O_2_ decomposition rates along with reaction time. Taking into account the acceleration of H_2_O_2_ decomposition under irradiation, the stability of the produced H_2_O_2_ was assessed by measuring the degradation behavior of H_2_O_2_ generated during the reaction of the prepared samples. The mixed solution of H_2_O_2_ (1 mM, 20 mL) and photocatalyst (1 mg mL^-1^) was sonicated for 2 minutes, followed by purging the system with argon. Subsequently, the light source was turned on, and the residual H_2_O_2_ concentration was measured every 15 minutes to assess its stability.

**
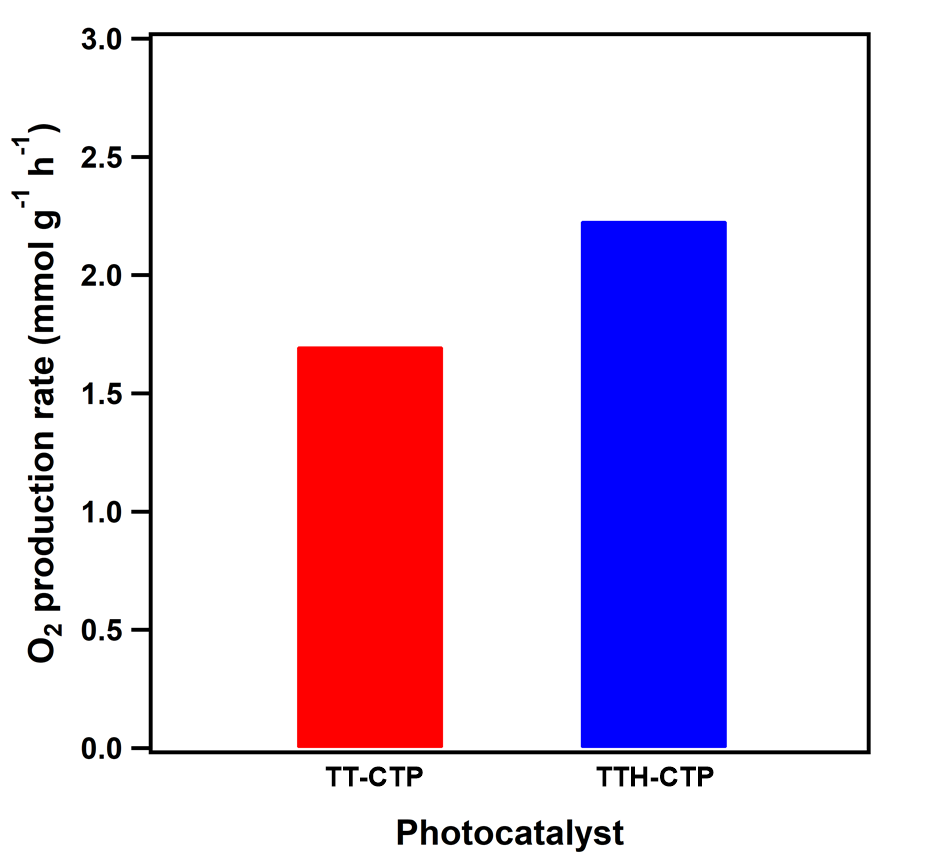
**

**Supplementary Figure S39.** O_2_ generation rates of photocatalytic half reaction with TT–CTP and TTH–CTP as the photocatalyst. The experiments were conducted in a system containing 10 mg photocatalyst, 0.17 g AgNO_3_, and 100 mL H_2_O at 12 °C.

**
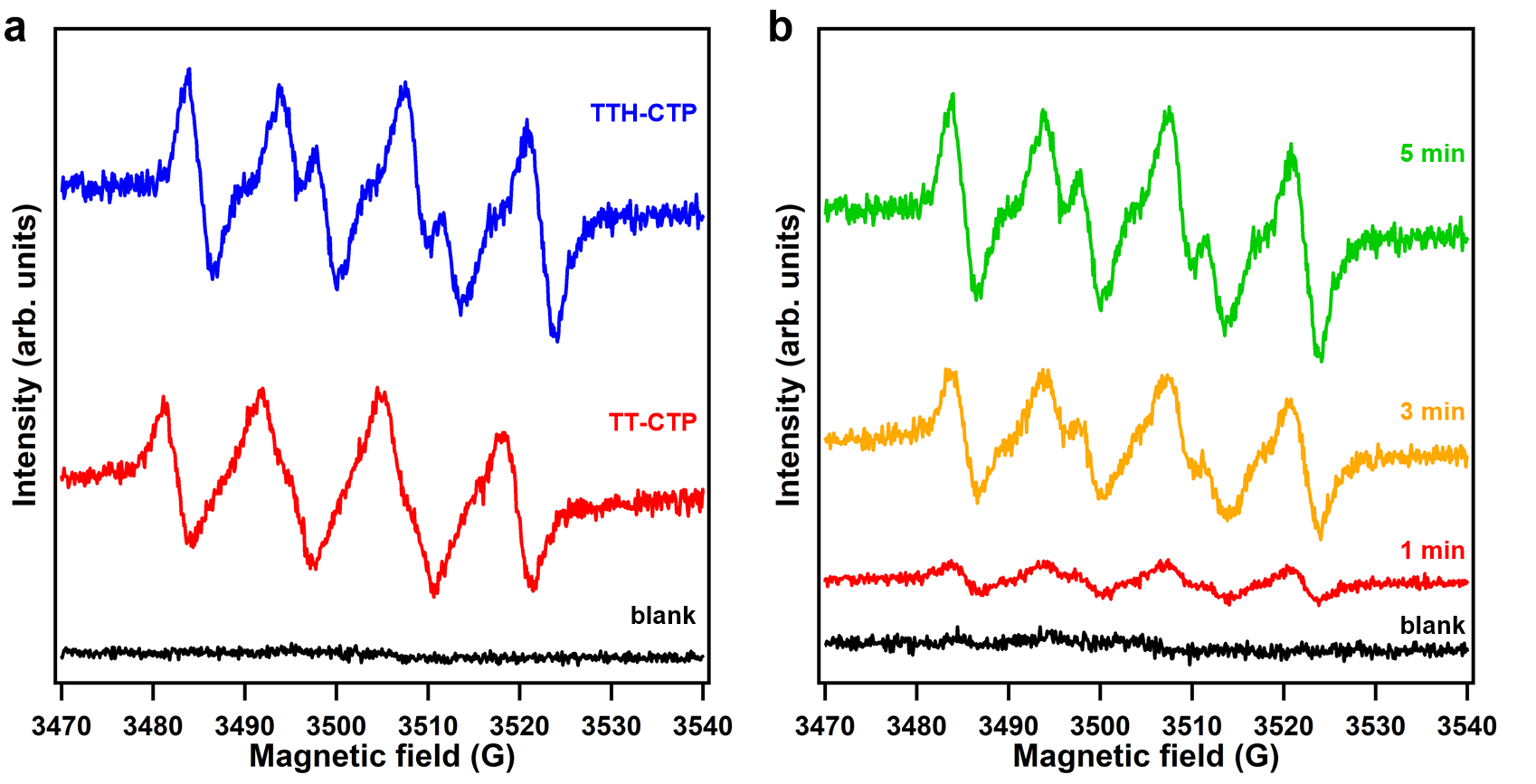
**

**Supplementary Figure S40.** Detection of the DMPO-^•^O_2_^–^ radicals. (a) EPR spectra of TT–CTP and TTH–CTP under O_2_ atmosphere. (b) Time-dependent EPR spectra of TTH–CTP under O_2_ atmosphere.

**
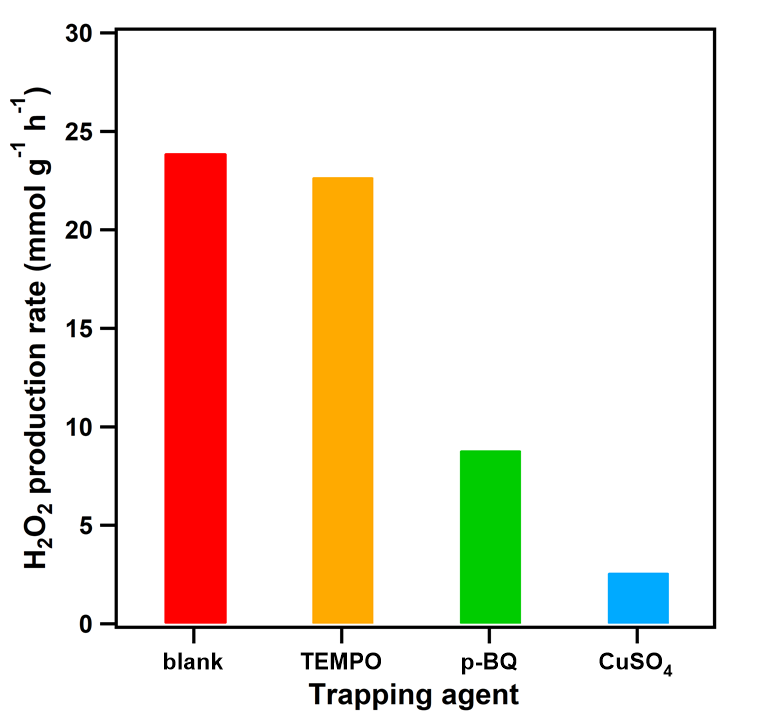
**

**Supplementary Figure S41.** H_2_O_2_ generation rates of photocatalytic half reaction of TTH–CTP by adding 2,2,6,6-tetramethylpiperidine (TEMP, 0.1 mM), 1,4-benzoquinone (p-BQ, 0.05 mM), and CuSO_4_ (0.05 mM) as trapping agents for singlet oxygen, superoxide radical, and free electron, respectively.

**Section 6: Theoretical studies**

**
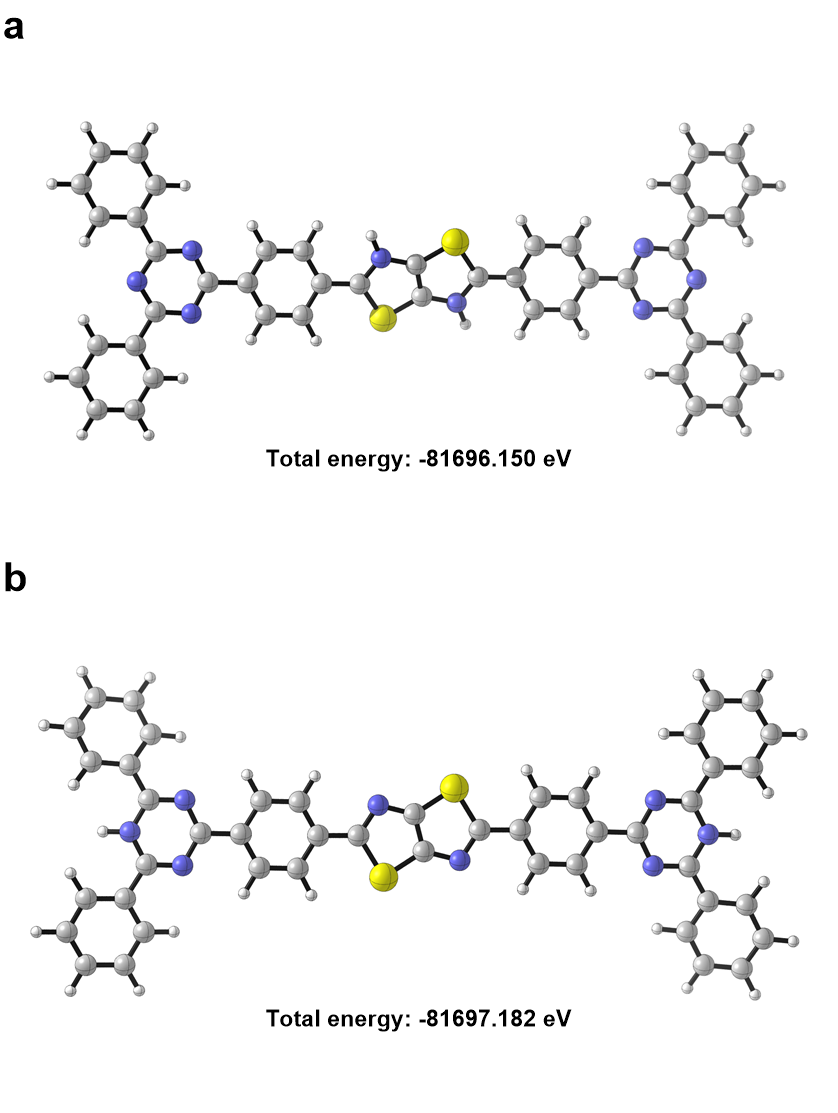
**

**Supplementary Figure S42.** Optimized geometrical structures and the total energies for model systems of (a) TT-protonated and (b) triazine-protonated TTH–CTP. C: grey; N: blue; H: white; S: yellow.

**
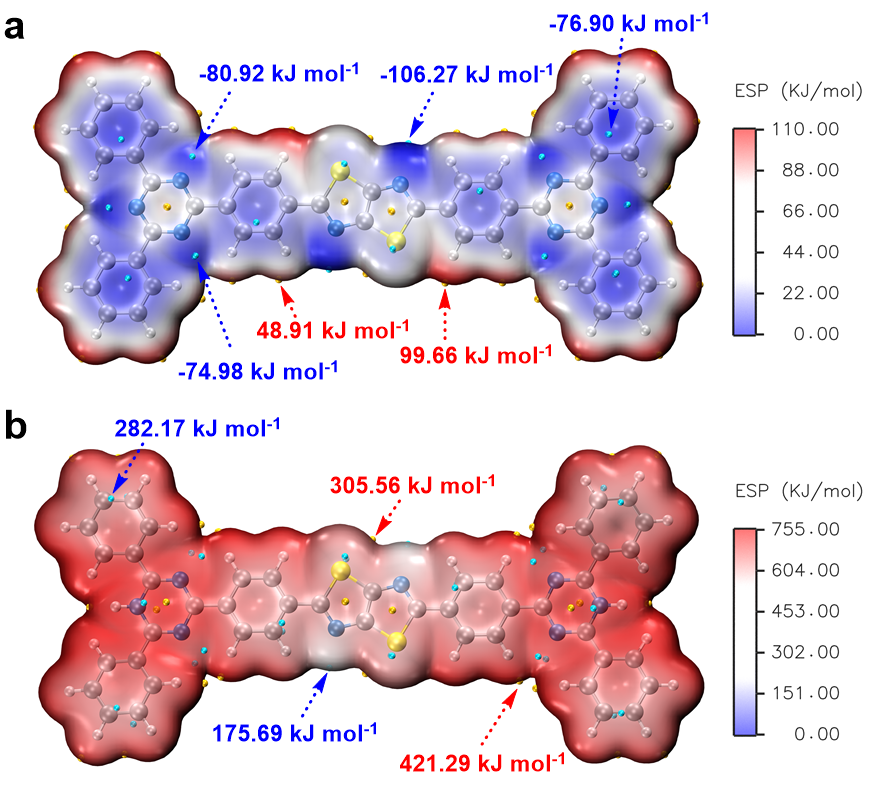
**

**Supplementary Figure S43.** Isosurface map of electrostatic potential (ESP) of (a) TT–CTP and (b) TTH–CTP. The maxima and minima of ESP are plotted as yellow and blue spheres, respectively. C: grey; N: blue; H: white; S: yellow.

**
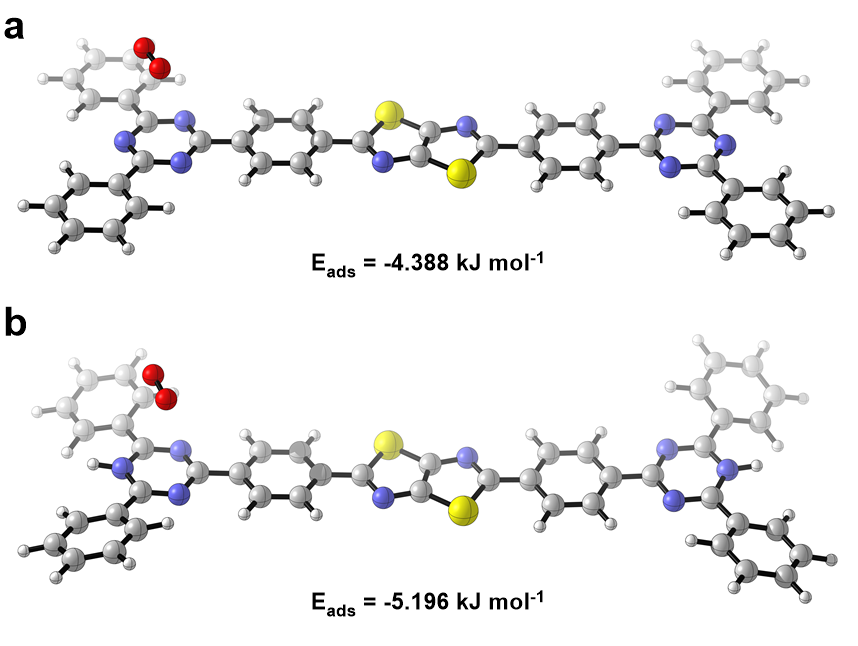
**

**Supplementary Figure S44.** O_2_-adsorption energies for model systems of (a) TT–CTP and (b) TTH–CTP. C: grey; N: blue; H: white; O: red; S: yellow.

**
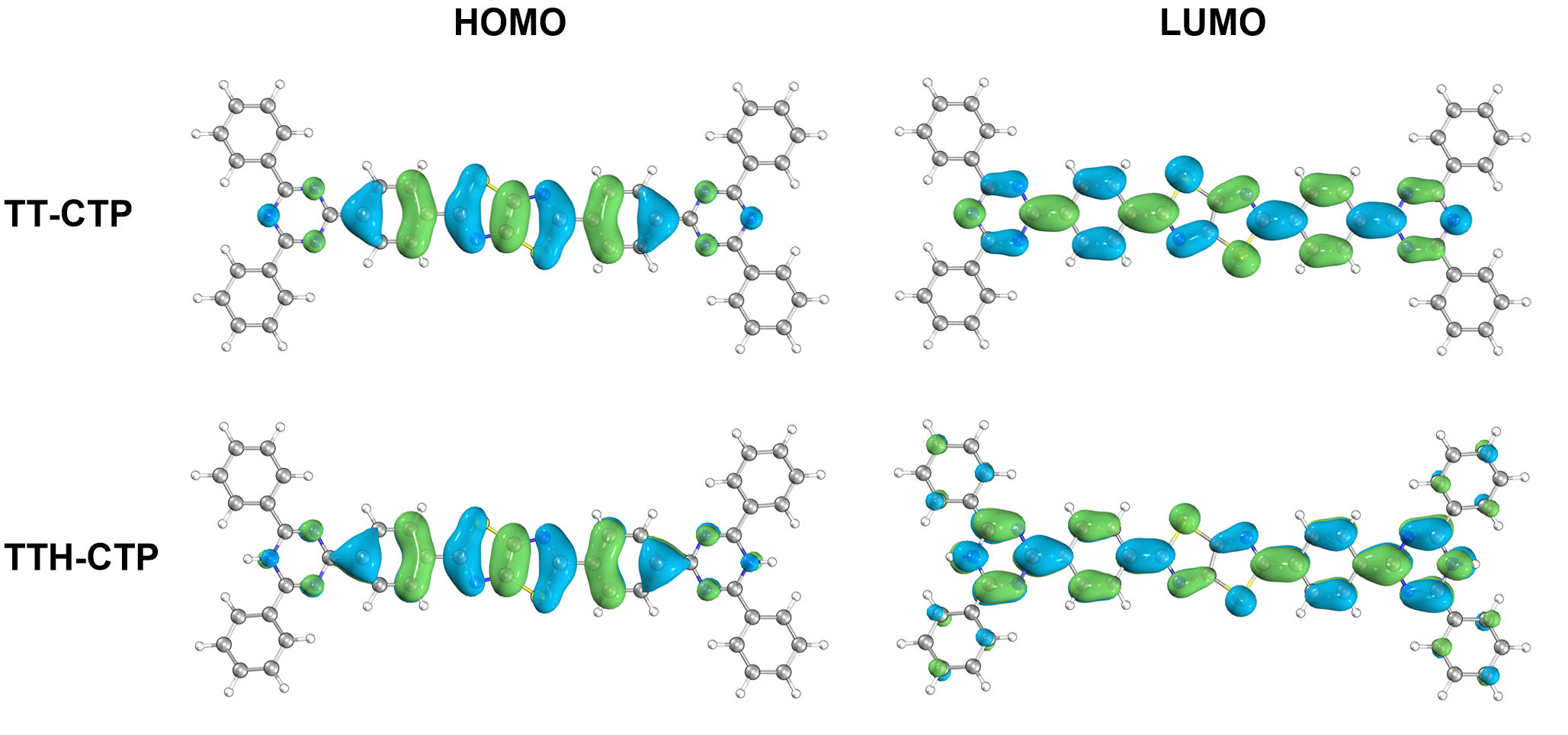
**

**Supplementary Figure S45.** HOMO/LUMO distributions of TT–CTP and TTH–CTP. C: grey; N: blue; H: white; S: yellow.

**
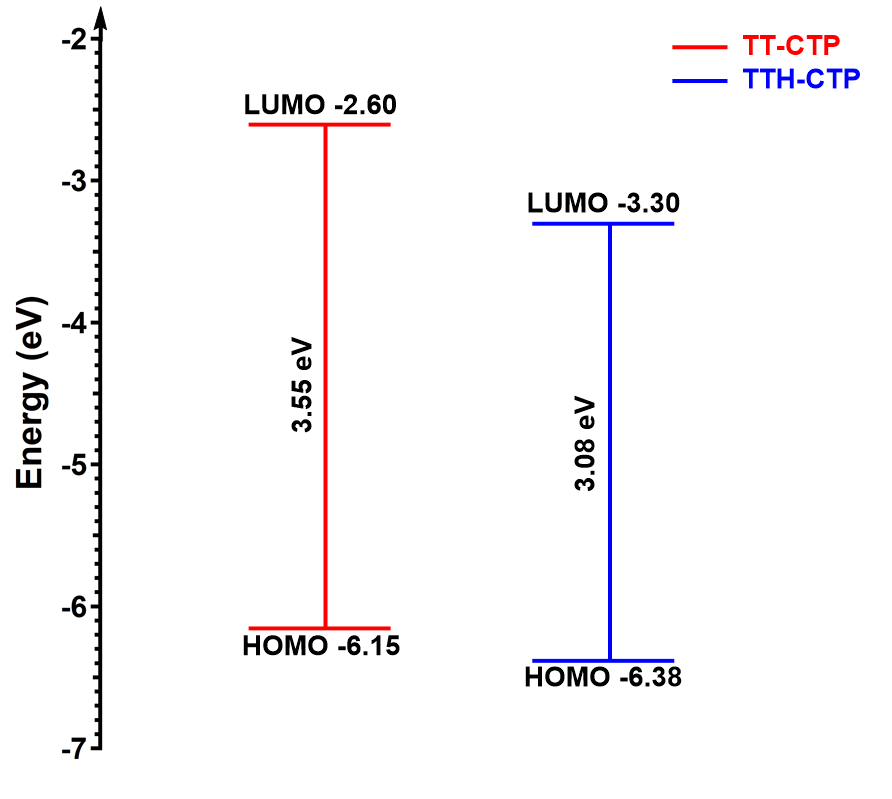
**

**Supplementary Figure S46.** Calculated HOMO/LUMO energy levels of TT–CTP and TTH–CTP.

**
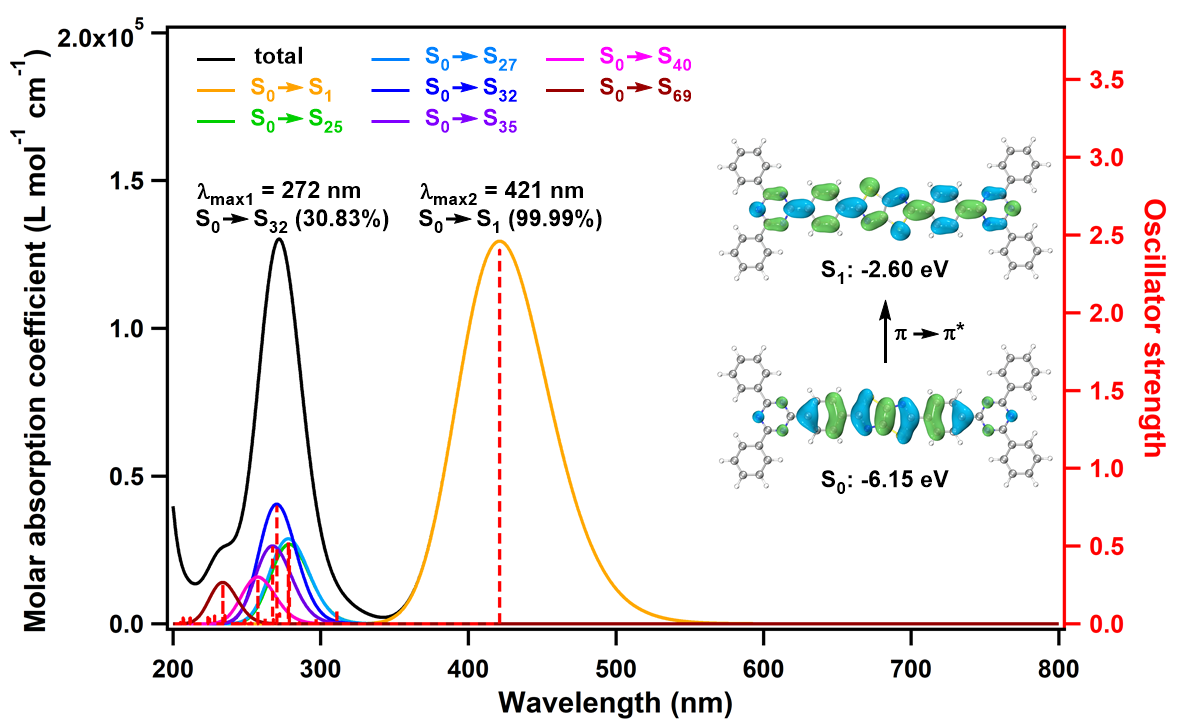
**

**Supplementary Figure S47.** TD-DFT-calculated absorption spectra and oscillator strengths for the model system of TT–CTP at the initial state. The insets are the transition orbits at the maximum oscillator strength.

**
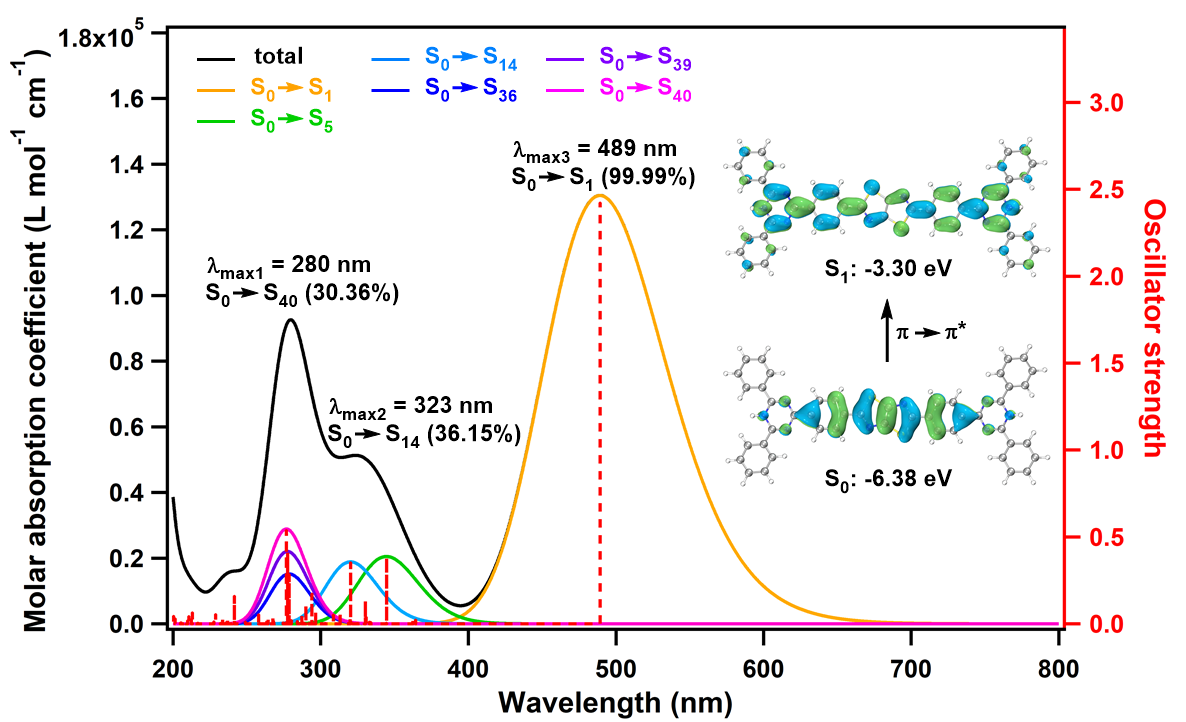
**

**Supplementary Figure S48.** TD-DFT-calculated absorption spectra and oscillator strengths for the model system of TTH–CTP at the initial state. The insets are the transition orbits at the maximum oscillator strength.

**Section 7: Exciton dissociation of the CTPs**

**
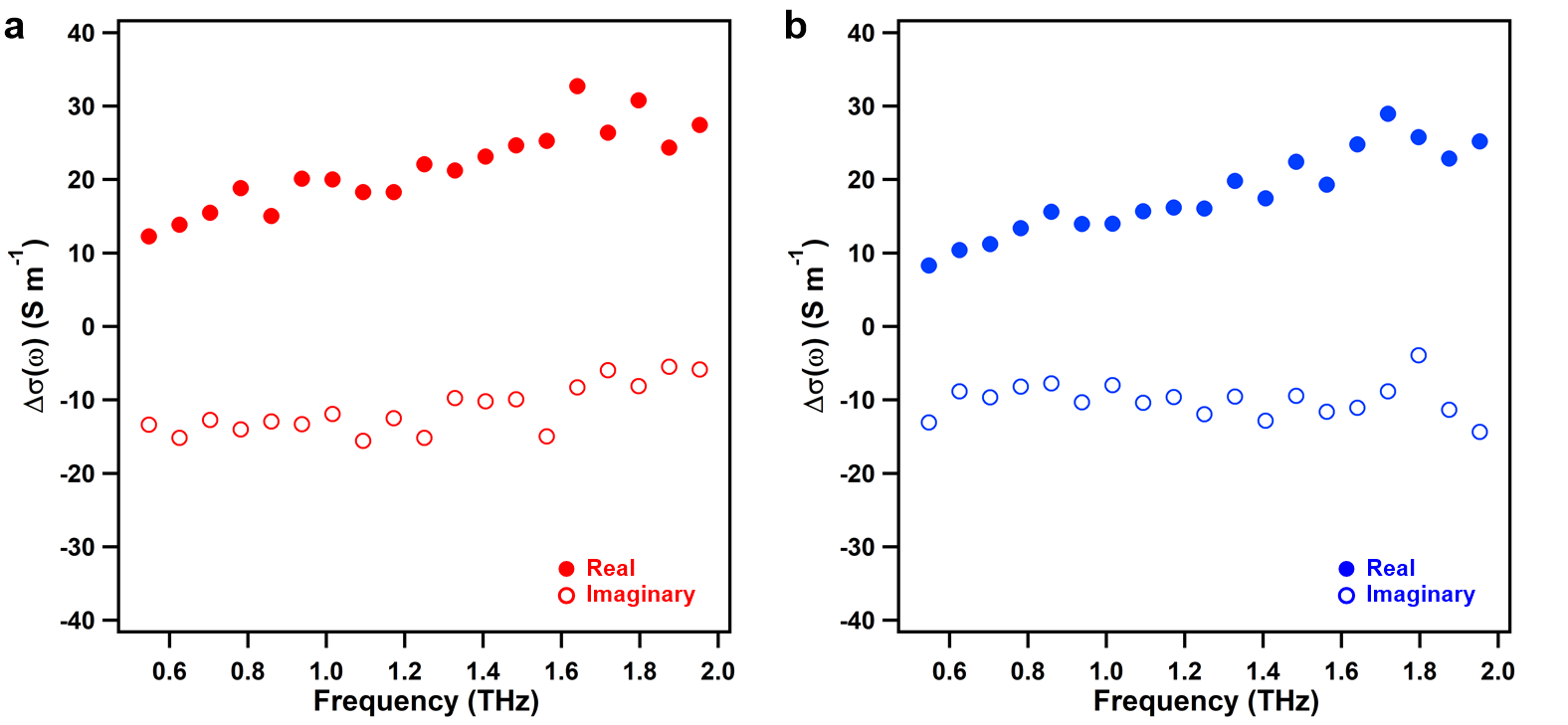
**

**Supplementary Figure S49.** Frequency-resolved complex photoconductivity of (a) TT–CTP and (b) TTH–CTP films. The solid and hollow data correspond to the real and imaginary parts of the complex photoconductivity, respectively.

**
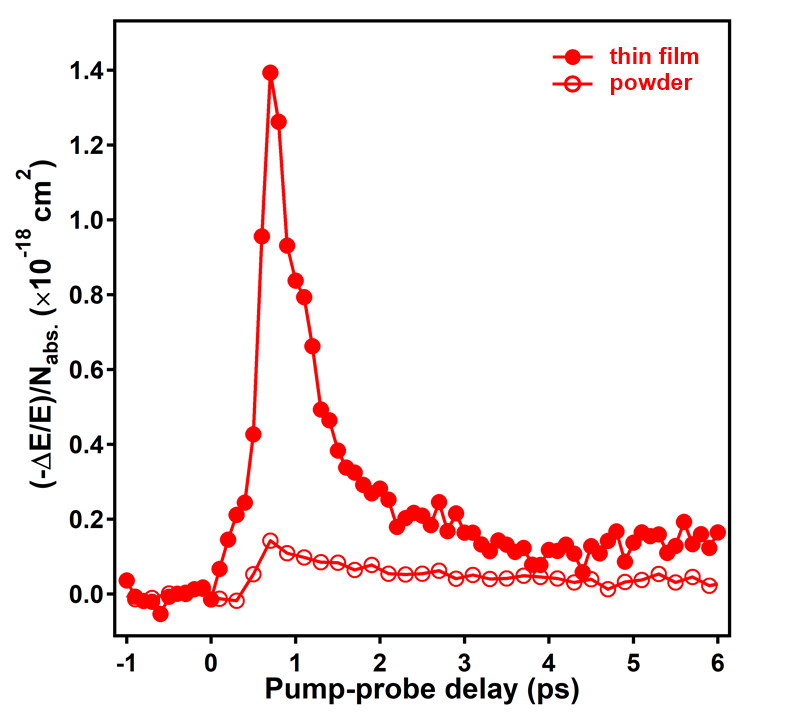
**

**Supplementary Figure S50.** Comparison of THz photoconductivity dynamics of TT–CTP thin film and powder.

**Supplementary Tables**

**Section 1: Comparison of H_2_O_2_ generation performances of reported photocatalysts**

**Supplementary Table S1.** **H_2_O_2_ generation rate and AQY of photocatalysts reported to date.**

| Material | Sacrificial reagent | Light | H_2_O_2_ generation rate (mmol g^–1^ h^–1^) | AQY | Ref |
| --- | --- | --- | --- | --- | --- |
| Au/TiO_2_ | ethanol | > 300 nm | 0.25 | 13% (355 nm) | S11 |
| Au/TiO_2_ | HCOOH | > 420 nm | 0.7 | 13.7% (450 nm) | S12 |
| CQD/TiO_2_ | ethanol | > 420 nm | 0.86 | 5.2% (365 nm) | S13 |
| g-C_3_N_4_/PDI/rGO | – | > 420 nm | 0.38 | 6.1% (420 nm) | S14 |
| (K,P,O)-g-C_3_N_4_ | ethanol | > 420 nm | 0.49 | 8% (420 nm) | S15 |
| O-enriched g-C_3_N_4_ | 2-propanol | > 420 nm | 1.2 | 10.2% (420 nm) | S16 |
| reduced g-C_3_N_4_ | – | > 420 nm | 0.17 | 4.3% (420 nm) | S17 |
| Au/BiVO_4_ | ethanol | > 420 nm | 2.41 | 0.24% (420 nm) | S18 |
| rGO/Cd_3_(TMT)_2_ | methanol | > 420 nm | 0.073 | 6.8% (450 nm) | S19 |
| EBA-COF | ethanol | > 420 nm | 1.82 | 4.4% (420 nm) | S20 |
| Bpt-CTF | – | Xe lamp | 3.27 | 8.6% (400 nm) | S21 |
| DBTP-COF | isopropanol | > 420 nm | 15 | 7.4% (450 nm) | S22 |
| TF-COF | ethanol | > 400 nm | 1.74 | 5.1% (400 nm) | S23 |
| CTF-NS-5BT | benzyl alcohol | > 420 nm | 1.63 | 6.6% (420 nm) | S24 |
| COF-TfpBpy | – | sun light | 0.69 | 8.1% (420 nm) | S25 |
| TTF-BT-COF | – | Xe lamp | 2.76 | 11.2% (420 nm) | S26 |
| TTH–CTP | benzyl alcohol | Xe lamp | 23.7 | 11.3% (450 nm) | This work |

**Section 2: Structural analyses of the CTPs**

**Supplementary Table S2. Elemental analysis results of TT–CTP and TTH–CTP.**

| Simple |  | C% | H% | N% | S% |
| --- | --- | --- | --- | --- | --- |
| TT–CTP | calcd. | 62.77 | 2.34 | 16.27 | 18.62 |
|  | found | 59.25 | 2.44 | 14.27 | 16.88 |
| TTH–CTP | calcd. | 62.77 | 2.34 | 16.27 | 18.62 |
|  | found | 58.63 | 2.74 | 14.99 | 17.05 |

**Supplementary Table S3. XPS analysis results of TT–CTF and TTH–CTF.**

| Sample | Element | Peak | Concentration (%) | |
| --- | --- | --- | --- | --- |
| TT–CTF | C | Peak 1 | 43.7 | 76.4 (in total) |
|  |  | Peak 2 | 22.0 |  |
|  |  | Peak 3 | 10.7 |  |
|  | N | Peak 1 | 15.1 | 16.1 (in total) |
|  |  | Peak 2 | 1.0 |  |
|  | S | Peak 1 | 4.8 | 7.5 (in total) |
|  |  | Peak 2 | 2.7 |  |
| TTH–CTF | C | Peak 1 | 38.4 | 76.9 (in total) |
|  |  | Peak 2 | 28.8 |  |
|  |  | Peak 3 | 9.7 |  |
|  | N | Peak 1 | 4.8 | 15.4 (in total) |
|  |  | Peak 2 | 7.3 |  |
|  |  | Peak 3 | 3.3 |  |
|  | S | Peak 1 | 4.6 | 7.7 (in total) |
|  |  | Peak 2 | 3.1 |  |

**Supplementary Table S4. Solubility of TTH–CTP in various solvents.**

| Solvent | acetonitrile | DMA | NMP | DMF | DMSO | DMI |
| --- | --- | --- | --- | --- | --- | --- |
| Concentration (mg mL^–1^) | 3.5 | 2.3 | 1.4 | 1.5 | 1.8 | 2.0 |

**Section 3: Theoretical calculations**

**Supplementary Table S5. Atomistic coordinates of model of TT–CTP calculated using DFT method at the PBE0/6-31G* level of theory.**

| ***Atom*** | ***x (Å)*** | ***y (Å)*** | ***z (Å)*** |
| --- | --- | --- | --- |
| C | 9.89260 | 1.13583 | -0.25468 |
| N | 8.55544 | 1.15932 | -0.26834 |
| C | 10.60962 | 2.39802 | -0.53360 |
| C | 5.74039 | 1.13815 | -0.25045 |
| C | 4.35452 | 1.11666 | -0.24699 |
| C | 3.66163 | -0.07956 | 0.00002 |
| C | 4.39792 | -1.24889 | 0.24231 |
| C | 5.78441 | -1.22452 | 0.23652 |
| C | 2.20132 | -0.08052 | 0.00257 |
| N | 1.46991 | 1.01223 | 0.00111 |
| S | -1.29346 | 1.60066 | 0.00194 |
| H | 6.26187 | 2.06947 | -0.44556 |
| H | 3.80019 | 2.02866 | -0.44346 |
| H | 3.89525 | -2.19024 | 0.45157 |
| H | 6.33764 | -2.13751 | 0.42969 |
| C | 0.16322 | 0.67507 | -0.00218 |
| C | 7.95322 | -0.00499 | -0.01010 |
| N | 8.59627 | -1.14718 | 0.24886 |
| C | 6.47554 | -0.03091 | -0.00877 |
| C | 10.02178 | -3.51480 | 0.77888 |
| C | 10.73929 | -4.67711 | 1.04391 |
| C | 12.13454 | -4.65249 | 1.05527 |
| C | 12.80943 | -3.45814 | 0.79925 |
| C | 12.09467 | -2.29423 | 0.53374 |
| H | 12.69406 | -5.56115 | 1.26304 |
| H | 8.93672 | -3.53559 | 0.77039 |
| H | 10.20826 | -5.60450 | 1.24243 |
| H | 13.89606 | -3.43307 | 0.80666 |
| H | 12.62103 | -1.36616 | 0.33506 |
| C | 9.93168 | -1.07521 | 0.24057 |
| N | 10.62141 | 0.04338 | -0.00473 |
| C | 10.69279 | -2.31098 | 0.52127 |
| C | 12.01108 | 2.43633 | -0.52003 |
| C | 12.68450 | 3.62517 | -0.78302 |
| C | 11.96819 | 4.78966 | -1.06303 |
| C | 10.57309 | 4.75927 | -1.07842 |
| C | 9.89681 | 3.57209 | -0.81515 |
| H | 12.49558 | 5.71778 | -1.26883 |
| H | 12.56951 | 1.53154 | -0.30240 |
| H | 13.77121 | 3.64307 | -0.76942 |
| H | 10.01005 | 5.66313 | -1.29628 |
| H | 8.81180 | 3.54992 | -0.82739 |
| C | -9.89162 | -1.13311 | -0.25915 |
| N | -8.55448 | -1.15467 | -0.27565 |
| C | -10.60748 | -2.39240 | -0.55367 |
| C | -5.73984 | -1.13246 | -0.25475 |
| C | -4.35400 | -1.11004 | -0.25244 |
| C | -3.66170 | 0.08489 | 0.00237 |
| C | -4.39853 | 1.25202 | 0.25344 |
| C | -5.78505 | 1.22663 | 0.24921 |
| C | -2.20138 | 0.08639 | 0.00404 |
| N | -1.46989 | -1.00627 | -0.00377 |
| S | 1.29330 | -1.59468 | -0.00744 |
| H | -6.26089 | -2.06273 | -0.45589 |
| H | -3.79924 | -2.02027 | -0.45574 |
| H | -3.89624 | 2.19224 | 0.46854 |
| H | -6.33872 | 2.13782 | 0.44948 |
| C | -0.16326 | -0.66901 | -0.00580 |
| C | -7.95322 | 0.00684 | -0.00300 |
| N | -8.59720 | 1.14463 | 0.27245 |
| C | -6.47556 | 0.03427 | -0.00374 |
| C | -10.02521 | 3.50493 | 0.82888 |
| C | -10.74396 | 4.66253 | 1.11072 |
| C | -12.13898 | 4.63357 | 1.13335 |
| C | -12.81245 | 3.43967 | 0.87153 |
| C | -12.09648 | 2.28056 | 0.58884 |
| H | -12.69946 | 5.53852 | 1.35434 |
| H | -8.94033 | 3.52910 | 0.81172 |
| H | -10.21404 | 5.58961 | 1.31361 |
| H | -13.89889 | 3.41118 | 0.88790 |
| H | -12.62164 | 1.35277 | 0.38570 |
| C | -9.93254 | 1.07096 | 0.26593 |
| N | -10.62135 | -0.04512 | 0.00694 |
| C | -10.69480 | 2.30168 | 0.56501 |
| C | -12.00887 | -2.43254 | -0.53901 |
| C | -12.68117 | -3.61877 | -0.81625 |
| C | -11.96379 | -4.77878 | -1.11169 |
| C | -10.56874 | -4.74649 | -1.12839 |
| C | -9.89358 | -3.56189 | -0.85100 |
| H | -12.49032 | -5.70488 | -1.32848 |
| H | -12.56809 | -1.53123 | -0.30928 |
| H | -13.76785 | -3.63816 | -0.80167 |
| H | -10.00486 | -5.64685 | -1.35826 |
| H | -8.80860 | -3.53817 | -0.86427 |

**Supplementary Table S6. Atomistic coordinates of model of TTH–CTP calculated using DFT method at the PBE0/6-31G* level of theory.**

| ***Atom*** | ***x (Å)*** | ***y (Å)*** | ***z (Å)*** |
| --- | --- | --- | --- |
| C | 9.84291 | 1.20767 | 0.02046 |
| N | 8.53096 | 1.18272 | -0.09618 |
| C | 10.55210 | 2.47866 | 0.13643 |
| C | 5.74416 | 1.13996 | -0.23331 |
| C | 4.36330 | 1.11152 | -0.27527 |
| C | 3.67910 | -0.11356 | -0.34330 |
| C | 4.41771 | -1.30629 | -0.37734 |
| C | 5.80016 | -1.27806 | -0.33602 |
| C | 2.22125 | -0.11090 | -0.36619 |
| N | 1.50173 | 0.98935 | -0.37250 |
| S | -1.25091 | 1.60746 | -0.37245 |
| H | 6.26196 | 2.09073 | -0.17657 |
| H | 3.79996 | 2.03743 | -0.25030 |
| H | 3.91877 | -2.26996 | -0.43588 |
| H | 6.35923 | -2.20658 | -0.35968 |
| C | 0.19524 | 0.66592 | -0.37580 |
| C | 7.94112 | -0.02080 | -0.18898 |
| N | 8.59951 | -1.19048 | -0.22686 |
| C | 6.48178 | -0.05435 | -0.25918 |
| C | 10.00423 | -3.60745 | -0.00798 |
| C | 10.70482 | -4.80437 | -0.00270 |
| C | 12.09536 | -4.79952 | -0.10405 |
| C | 12.78552 | -3.59272 | -0.21136 |
| C | 12.09357 | -2.38973 | -0.21313 |
| H | 12.64329 | -5.73737 | -0.10153 |
| H | 8.92312 | -3.60339 | 0.07571 |
| H | 10.16595 | -5.74297 | 0.08268 |
| H | 13.86743 | -3.58768 | -0.29934 |
| H | 12.66331 | -1.47098 | -0.31915 |
| C | 9.91198 | -1.15477 | -0.10322 |
| N | 10.53751 | 0.04122 | 0.04642 |
| C | 10.69298 | -2.38822 | -0.10947 |
| C | 11.92679 | 2.59175 | -0.12333 |
| C | 12.55402 | 3.82383 | -0.00056 |
| C | 11.82318 | 4.94670 | 0.38536 |
| C | 10.45652 | 4.84020 | 0.64120 |
| C | 9.81916 | 3.61488 | 0.51290 |
| H | 12.31992 | 5.90770 | 0.48320 |
| H | 12.51227 | 1.73914 | -0.45558 |
| H | 13.61502 | 3.90856 | -0.21395 |
| H | 9.88729 | 5.71414 | 0.94281 |
| H | 8.75771 | 3.52293 | 0.71593 |
| C | -9.79941 | -1.18364 | 0.06418 |
| N | -8.48785 | -1.16861 | -0.06133 |
| C | -10.51426 | -2.44590 | 0.22669 |
| C | -5.69743 | -1.13907 | -0.18448 |
| C | -4.31636 | -1.11492 | -0.22639 |
| C | -3.62971 | 0.10537 | -0.34047 |
| C | -4.36590 | 1.29742 | -0.41984 |
| C | -5.74826 | 1.27357 | -0.37798 |
| C | -2.17189 | 0.10038 | -0.36446 |
| N | -1.45276 | -1.00015 | -0.36468 |
| S | 1.30054 | -1.61817 | -0.36573 |
| H | -6.21710 | -2.08603 | -0.09188 |
| H | -3.75491 | -2.04035 | -0.16537 |
| H | -3.86531 | 2.25689 | -0.51900 |
| H | -6.30530 | 2.20154 | -0.43882 |
| C | -0.14608 | -0.67695 | -0.37154 |
| C | -7.89192 | 0.02856 | -0.18928 |
| N | -8.54591 | 1.19904 | -0.27150 |
| C | -6.43249 | 0.05501 | -0.25589 |
| C | -9.96639 | 3.62385 | -0.03122 |
| C | -10.67112 | 4.81752 | -0.08073 |
| C | -12.04398 | 4.80806 | -0.32504 |
| C | -12.71303 | 3.60067 | -0.52163 |
| C | -12.01811 | 2.40042 | -0.46623 |
| H | -12.59400 | 5.74380 | -0.36625 |
| H | -8.89971 | 3.62250 | 0.16482 |
| H | -10.14983 | 5.75728 | 0.07381 |
| H | -13.77908 | 3.59345 | -0.72623 |
| H | -12.55891 | 1.47698 | -0.65426 |
| C | -9.85809 | 1.17172 | -0.14883 |
| N | -10.48894 | -0.01355 | 0.05598 |
| C | -10.63650 | 2.40457 | -0.21644 |
| C | -11.88854 | -2.56158 | -0.03447 |
| C | -12.52278 | -3.78469 | 0.13369 |
| C | -11.79937 | -4.89534 | 0.56628 |
| C | -10.43314 | -4.78618 | 0.82353 |
| C | -9.78866 | -3.57014 | 0.65024 |
| H | -12.30166 | -5.84918 | 0.69969 |
| H | -12.46718 | -1.71879 | -0.40223 |
| H | -13.58333 | -3.87240 | -0.08072 |
| H | -9.87003 | -5.65057 | 1.16189 |
| H | -8.72772 | -3.47557 | 0.85491 |
| H | -11.48669 | -0.02077 | 0.25093 |
| H | 11.53915 | 0.06262 | 0.21331 |

**Supplementary Table S7. Atomistic coordinates of model of Abs_O_2_ on TT–CTP calculated using DFT method at the PBE0/6-31G* level of theory.**

| ***Atom*** | ***x (Å)*** | ***y (Å)*** | ***z (Å)*** |
| --- | --- | --- | --- |
| C | 9.90570 | 1.14154 | 0.16616 |
| N | 8.56845 | 1.16447 | 0.17413 |
| C | 10.62103 | 2.40715 | 0.43371 |
| C | 5.75311 | 1.13915 | 0.15455 |
| C | 4.36726 | 1.11585 | 0.14838 |
| C | 3.67639 | -0.08254 | -0.09377 |
| C | 4.41470 | -1.25205 | -0.32892 |
| C | 5.80112 | -1.22596 | -0.31997 |
| C | 2.21609 | -0.08588 | -0.09787 |
| N | 1.48272 | 1.00556 | -0.10086 |
| S | -1.28176 | 1.58901 | -0.10363 |
| H | 6.27298 | 2.07210 | 0.34605 |
| H | 3.81138 | 2.02810 | 0.33920 |
| H | 3.91371 | -2.19506 | -0.53469 |
| H | 6.35588 | -2.13926 | -0.50715 |
| C | 0.17662 | 0.66608 | -0.09673 |
| C | 7.96789 | -0.00307 | -0.07319 |
| N | 8.61259 | -1.14775 | -0.31665 |
| C | 6.49024 | -0.03030 | -0.07911 |
| C | 10.04117 | -3.51752 | -0.82792 |
| C | 10.76019 | -4.68248 | -1.07664 |
| C | 12.15550 | -4.66069 | -1.06708 |
| C | 12.82891 | -3.46637 | -0.80706 |
| C | 12.11264 | -2.29966 | -0.55850 |
| H | 12.71620 | -5.57150 | -1.26181 |
| H | 8.95606 | -3.53613 | -0.83546 |
| H | 10.23029 | -5.60978 | -1.27859 |
| H | 13.91557 | -3.44355 | -0.79797 |
| H | 12.63792 | -1.37167 | -0.35658 |
| C | 9.94791 | -1.07501 | -0.30369 |
| N | 10.63606 | 0.04668 | -0.06833 |
| C | 10.71069 | -2.31362 | -0.56678 |
| C | 12.02248 | 2.44659 | 0.42244 |
| C | 12.69435 | 3.63854 | 0.67506 |
| C | 11.97650 | 4.80503 | 0.94246 |
| C | 10.58140 | 4.77355 | 0.95546 |
| C | 9.90666 | 3.58329 | 0.70236 |
| H | 12.50267 | 5.73557 | 1.14030 |
| H | 12.58212 | 1.54021 | 0.21471 |
| H | 13.78107 | 3.65729 | 0.66327 |
| H | 10.01717 | 5.67897 | 1.16356 |
| H | 8.82165 | 3.56032 | 0.71275 |
| C | -9.87427 | -1.16030 | 0.17549 |
| N | -8.53707 | -1.18059 | 0.18689 |
| C | -10.58740 | -2.42021 | 0.47474 |
| C | -5.72268 | -1.15278 | 0.16306 |
| C | -4.33691 | -1.12737 | 0.16015 |
| C | -3.64723 | 0.06858 | -0.09706 |
| C | -4.38666 | 1.23367 | -0.34983 |
| C | -5.77310 | 1.20540 | -0.34475 |
| C | -2.18693 | 0.07304 | -0.09946 |
| N | -1.45340 | -1.01823 | -0.08819 |
| S | 1.31075 | -1.60164 | -0.08253 |
| H | -6.24169 | -2.08379 | 0.36605 |
| H | -3.78011 | -2.03612 | 0.36453 |
| H | -3.88646 | 2.17453 | -0.56694 |
| H | -6.32884 | 2.11504 | -0.54640 |
| C | -0.14742 | -0.67857 | -0.08808 |
| C | -7.93868 | -0.01805 | -0.08764 |
| N | -8.58477 | 1.11755 | -0.36685 |
| C | -6.46094 | 0.01214 | -0.08887 |
| C | -10.01787 | 3.47659 | -0.91474 |
| C | -10.73915 | 4.63335 | -1.19364 |
| C | -12.13422 | 4.60253 | -1.21161 |
| C | -12.80517 | 3.40754 | -0.94817 |
| C | -12.08672 | 2.24924 | -0.66847 |
| H | -12.69666 | 5.50681 | -1.43027 |
| H | -8.93298 | 3.50214 | -0.90114 |
| H | -10.21115 | 5.56126 | -1.39770 |
| H | -13.89162 | 3.37760 | -0.96086 |
| H | -12.60991 | 1.32062 | -0.46409 |
| C | -9.91993 | 1.04239 | -0.35317 |
| N | -10.60659 | -0.07469 | -0.09282 |
| C | -10.68499 | 2.27226 | -0.64956 |
| C | -11.98865 | -2.46396 | 0.45878 |
| C | -12.65821 | -3.65086 | 0.73980 |
| C | -11.93815 | -4.80799 | 1.04005 |
| C | -10.54319 | -4.77214 | 1.05775 |
| C | -9.87077 | -3.58692 | 0.77639 |
| H | -12.46256 | -5.73460 | 1.25979 |
| H | -12.54988 | -1.56490 | 0.22513 |
| H | -13.74482 | -3.67308 | 0.72420 |
| H | -9.97725 | -5.67026 | 1.29129 |
| H | -8.78586 | -3.56036 | 0.79034 |
| O | -9.80867 | 1.69551 | 3.16220 |
| O | -9.17495 | 0.67568 | 3.22197 |

**Supplementary Table S8. Atomistic coordinates of model of Abs_O_2_ on TTH–CTP calculated using DFT method at the PBE0/6-31G* level of theory.**

| ***Atom*** | ***x (Å)*** | ***y (Å)*** | ***z (Å)*** |
| --- | --- | --- | --- |
| C | 9.82685 | 1.20353 | 0.05265 |
| N | 8.51549 | 1.18071 | -0.07410 |
| C | 10.53676 | 2.47230 | 0.18348 |
| C | 5.72575 | 1.13989 | -0.20373 |
| C | 4.34479 | 1.11062 | -0.24474 |
| C | 3.66151 | -0.11448 | -0.32187 |
| C | 4.40100 | -1.30635 | -0.36477 |
| C | 5.78334 | -1.27735 | -0.32477 |
| C | 2.20372 | -0.11375 | -0.34669 |
| N | 1.48213 | 0.98512 | -0.35507 |
| S | -1.27195 | 1.59757 | -0.36383 |
| H | 6.24285 | 2.09064 | -0.14040 |
| H | 3.78075 | 2.03590 | -0.21250 |
| H | 3.90288 | -2.26992 | -0.43104 |
| H | 6.34303 | -2.20515 | -0.35680 |
| C | 0.17625 | 0.65909 | -0.36173 |
| C | 7.92348 | -0.02100 | -0.17366 |
| N | 8.58131 | -1.19118 | -0.22738 |
| C | 6.46419 | -0.05370 | -0.23985 |
| C | 10.01345 | -3.60252 | 0.09461 |
| C | 10.72367 | -4.79391 | 0.07850 |
| C | 12.09409 | -4.78625 | -0.17926 |
| C | 12.75536 | -3.58315 | -0.42305 |
| C | 12.05511 | -2.38488 | -0.40093 |
| H | 12.64833 | -5.72028 | -0.19421 |
| H | 8.94869 | -3.59938 | 0.30099 |
| H | 10.20857 | -5.73034 | 0.26975 |
| H | 13.81930 | -3.57807 | -0.63839 |
| H | 12.58833 | -1.46507 | -0.62542 |
| C | 9.89302 | -1.15613 | -0.10251 |
| N | 10.51990 | 0.03567 | 0.07454 |
| C | 10.67612 | -2.38728 | -0.13741 |
| C | 11.91313 | 2.58437 | -0.06808 |
| C | 12.54187 | 3.81448 | 0.06596 |
| C | 11.81075 | 4.93622 | 0.45471 |
| C | 10.44251 | 4.83066 | 0.70259 |
| C | 9.80368 | 3.60731 | 0.56341 |
| H | 12.30857 | 5.89577 | 0.56104 |
| H | 12.49830 | 1.73222 | -0.40218 |
| H | 13.60415 | 3.89878 | -0.14115 |
| H | 9.87330 | 5.70381 | 1.00651 |
| H | 8.74103 | 3.51588 | 0.76033 |
| C | -9.81614 | -1.20845 | 0.05639 |
| N | -8.50449 | -1.19250 | -0.06794 |
| C | -10.52949 | -2.47081 | 0.22650 |
| C | -5.71304 | -1.15793 | -0.18104 |
| C | -4.33186 | -1.13075 | -0.21825 |
| C | -3.64773 | 0.09020 | -0.34017 |
| C | -4.38646 | 1.27970 | -0.43259 |
| C | -5.76889 | 1.25282 | -0.39497 |
| C | -2.18980 | 0.08850 | -0.35871 |
| N | -1.46835 | -1.01047 | -0.35553 |
| S | 1.28594 | -1.62284 | -0.34765 |
| H | -6.23075 | -2.10531 | -0.08194 |
| H | -3.76843 | -2.05425 | -0.14699 |
| H | -3.88786 | 2.23938 | -0.53925 |
| H | -6.32794 | 2.17886 | -0.46554 |
| C | -0.16236 | -0.68448 | -0.35797 |
| C | -7.91038 | 0.00496 | -0.20131 |
| N | -8.56589 | 1.17370 | -0.29467 |
| C | -6.45065 | 0.03373 | -0.26489 |
| C | -9.99005 | 3.59566 | -0.02446 |
| C | -10.69600 | 4.78897 | -0.06723 |
| C | -12.06651 | 4.78044 | -0.32438 |
| C | -12.73239 | 3.57444 | -0.53962 |
| C | -12.03650 | 2.37442 | -0.49072 |
| H | -12.61733 | 5.71591 | -0.36059 |
| H | -8.92526 | 3.59316 | 0.18173 |
| H | -10.17752 | 5.72764 | 0.10267 |
| H | -13.79666 | 3.56824 | -0.75336 |
| H | -12.57397 | 1.45178 | -0.69194 |
| C | -9.87732 | 1.14543 | -0.16645 |
| N | -10.50782 | -0.03996 | 0.03651 |
| C | -10.65705 | 2.37805 | -0.23007 |
| C | -11.90262 | -2.59119 | -0.03850 |
| C | -12.53525 | -3.81394 | 0.13838 |
| C | -11.81150 | -4.91935 | 0.58365 |
| C | -10.44643 | -4.80544 | 0.84483 |
| C | -9.80346 | -3.58986 | 0.66277 |
| H | -12.31267 | -5.87278 | 0.72405 |
| H | -12.48167 | -1.75271 | -0.41548 |
| H | -13.59489 | -3.90547 | -0.07891 |
| H | -9.88306 | -5.66565 | 1.19327 |
| H | -8.74349 | -3.49146 | 0.87063 |
| H | -11.50557 | -0.04693 | 0.23160 |
| H | 11.51696 | 0.04986 | 0.27246 |
| O | -9.39195 | 1.42581 | 3.09474 |
| O | -8.89882 | 0.33448 | 2.99237 |

**Supplementary Table S9. Atomistic coordinates of model of Act_1 on TT–CTP calculated using DFT method at the PBE0/6-31G* level of theory.**

| ***Atom*** | ***x (Å)*** | ***y (Å)*** | ***z (Å)*** |
| --- | --- | --- | --- |
| C | 9.91882 | 1.17050 | -0.10882 |
| N | 8.58792 | 1.20020 | -0.08982 |
| C | 10.63847 | 2.46471 | -0.15206 |
| C | 5.76182 | 1.17286 | -0.04819 |
| C | 4.38297 | 1.15226 | -0.03559 |
| C | 3.66775 | -0.07276 | -0.00997 |
| C | 4.42743 | -1.26893 | 0.00586 |
| C | 5.80725 | -1.24194 | -0.00626 |
| C | 2.23724 | -0.07872 | -0.00410 |
| N | 1.47385 | 1.01872 | -0.02059 |
| S | -1.28088 | 1.60734 | -0.03318 |
| H | 6.27948 | 2.12695 | -0.06933 |
| H | 3.83222 | 2.08770 | -0.04701 |
| H | 3.92791 | -2.23525 | 0.02682 |
| H | 6.35781 | -2.17736 | 0.00524 |
| C | 0.18576 | 0.67917 | -0.01555 |
| C | 7.97060 | 0.00368 | -0.05317 |
| N | 8.63093 | -1.17013 | -0.03502 |
| C | 6.51535 | -0.02197 | -0.03489 |
| C | 10.06069 | -3.59377 | 0.02696 |
| C | 10.78157 | -4.78402 | 0.04344 |
| C | 12.17628 | -4.76039 | -0.00461 |
| C | 12.84579 | -3.53786 | -0.06801 |
| C | 12.12663 | -2.34604 | -0.08362 |
| H | 12.73878 | -5.69063 | 0.00765 |
| H | 8.97613 | -3.61387 | 0.06409 |
| H | 10.25345 | -5.73280 | 0.09375 |
| H | 13.93190 | -3.51199 | -0.10571 |
| H | 12.64900 | -1.39600 | -0.13308 |
| C | 9.95975 | -1.09328 | -0.05557 |
| N | 10.65663 | 0.05142 | -0.09260 |
| C | 10.72568 | -2.36116 | -0.03680 |
| C | 12.03855 | 2.49763 | -0.21387 |
| C | 12.71438 | 3.71392 | -0.25579 |
| C | 12.00186 | 4.91341 | -0.23570 |
| C | 10.60774 | 4.88929 | -0.17350 |
| C | 9.93012 | 3.67445 | -0.13291 |
| H | 12.53051 | 5.86282 | -0.26811 |
| H | 12.59441 | 1.56562 | -0.22995 |
| H | 13.80028 | 3.72525 | -0.30479 |
| H | 10.04614 | 5.81986 | -0.15698 |
| H | 8.84597 | 3.65761 | -0.08489 |
| C | -9.88865 | -1.15981 | -0.09644 |
| N | -8.55775 | -1.19234 | -0.07533 |
| C | -10.61173 | -2.45295 | -0.08976 |
| C | -5.73089 | -1.16842 | -0.03227 |
| C | -4.35200 | -1.14913 | -0.01472 |
| C | -3.63533 | 0.07525 | -0.02378 |
| C | -4.39343 | 1.27225 | -0.04802 |
| C | -5.77316 | 1.24661 | -0.06570 |
| C | -2.20482 | 0.08046 | -0.01289 |
| N | -1.44163 | -1.01703 | 0.00653 |
| S | 1.31345 | -1.60566 | 0.02016 |
| H | -6.24981 | -2.12205 | -0.02618 |
| H | -3.80245 | -2.08516 | 0.00446 |
| H | -3.89275 | 2.23817 | -0.05430 |
| H | -6.32256 | 2.18258 | -0.08553 |
| C | -0.15343 | -0.67759 | 0.00488 |
| C | -7.93812 | 0.00352 | -0.08256 |
| N | -8.59524 | 1.17866 | -0.11590 |
| C | -6.48273 | 0.02720 | -0.05930 |
| C | -10.01946 | 3.60620 | -0.14004 |
| C | -10.73755 | 4.79793 | -0.16753 |
| C | -12.13219 | 4.77580 | -0.21845 |
| C | -12.80441 | 3.55329 | -0.24072 |
| C | -12.08804 | 2.36000 | -0.21242 |
| H | -12.69254 | 5.70716 | -0.24021 |
| H | -8.93496 | 3.62511 | -0.10060 |
| H | -10.20723 | 5.74665 | -0.14923 |
| H | -13.89046 | 3.52856 | -0.28057 |
| H | -12.61252 | 1.41000 | -0.22982 |
| C | -9.92417 | 1.10400 | -0.13215 |
| N | -10.62393 | -0.03954 | -0.12780 |
| C | -10.68720 | 2.37363 | -0.16214 |
| C | -12.01216 | -2.48394 | -0.14444 |
| C | -12.69150 | -3.69897 | -0.14066 |
| C | -11.98213 | -4.89907 | -0.08188 |
| C | -10.58768 | -4.87679 | -0.02657 |
| C | -9.90654 | -3.66324 | -0.03126 |
| H | -12.51352 | -5.84749 | -0.07880 |
| H | -12.56529 | -1.55128 | -0.19052 |
| H | -13.77765 | -3.70890 | -0.18424 |
| H | -10.02859 | -5.80787 | 0.01984 |
| H | -8.82214 | -3.64785 | 0.01157 |
| O | -9.86742 | 0.99341 | 3.41626 |
| O | -9.23506 | -0.02664 | 3.34664 |

**Supplementary Table S10. Atomistic coordinates of model of Act_1 on TTH–CTP calculated using DFT method at the PBE0/6-31G* level of theory.**

| ***Atom*** | ***x (Å)*** | ***y (Å)*** | ***z (Å)*** |
| --- | --- | --- | --- |
| C | 9.83857 | 1.21175 | 0.03150 |
| N | 8.53102 | 1.18198 | -0.10446 |
| C | 10.54414 | 2.48640 | 0.14530 |
| C | 5.74013 | 1.12599 | -0.25582 |
| C | 4.36192 | 1.09066 | -0.30011 |
| C | 3.67695 | -0.13951 | -0.34374 |
| C | 4.42720 | -1.32972 | -0.35250 |
| C | 5.80711 | -1.29420 | -0.31122 |
| C | 2.22803 | -0.14571 | -0.36882 |
| N | 1.49376 | 0.95035 | -0.39999 |
| S | -1.26149 | 1.56012 | -0.41831 |
| H | 6.25302 | 2.08051 | -0.21733 |
| H | 3.79489 | 2.01478 | -0.29525 |
| H | 3.93233 | -2.29653 | -0.39212 |
| H | 6.36979 | -2.22091 | -0.31697 |
| C | 0.19425 | 0.62279 | -0.39751 |
| C | 7.93930 | -0.02572 | -0.18549 |
| N | 8.60811 | -1.19529 | -0.21523 |
| C | 6.48853 | -0.06569 | -0.25712 |
| C | 10.05694 | -3.59299 | 0.16881 |
| C | 10.77615 | -4.77928 | 0.17337 |
| C | 12.14497 | -4.76718 | -0.09302 |
| C | 12.79506 | -3.56447 | -0.36584 |
| C | 12.08524 | -2.37116 | -0.36484 |
| H | 12.70622 | -5.69715 | -0.09173 |
| H | 8.99342 | -3.59336 | 0.38177 |
| H | 10.26919 | -5.71525 | 0.38761 |
| H | 13.85775 | -3.55552 | -0.58747 |
| H | 12.60932 | -1.45176 | -0.61154 |
| C | 9.91593 | -1.15050 | -0.08250 |
| N | 10.53939 | 0.04729 | 0.07895 |
| C | 10.70818 | -2.37809 | -0.09334 |
| C | 11.92191 | 2.59854 | -0.09682 |
| C | 12.54634 | 3.83313 | 0.01911 |
| C | 11.80929 | 4.95995 | 0.38027 |
| C | 10.43941 | 4.85427 | 0.61913 |
| C | 9.80559 | 3.62622 | 0.49836 |
| H | 12.30336 | 5.92291 | 0.47246 |
| H | 12.51268 | 1.74180 | -0.40856 |
| H | 13.61019 | 3.91630 | -0.18050 |
| H | 9.86489 | 5.73107 | 0.90204 |
| H | 8.74167 | 3.53510 | 0.68860 |
| C | -9.81984 | -1.22173 | 0.03755 |
| N | -8.52428 | -1.21940 | -0.07184 |
| C | -10.56097 | -2.48017 | 0.18346 |
| C | -5.70565 | -1.19428 | -0.21381 |
| C | -4.33148 | -1.17473 | -0.26011 |
| C | -3.62126 | 0.04567 | -0.35038 |
| C | -4.37056 | 1.24254 | -0.40402 |
| C | -5.74557 | 1.22541 | -0.36146 |
| C | -2.18437 | 0.03965 | -0.37352 |
| N | -1.44444 | -1.05981 | -0.34934 |
| S | 1.30720 | -1.65764 | -0.33583 |
| H | -6.22588 | -2.14304 | -0.13914 |
| H | -3.77670 | -2.10644 | -0.22128 |
| H | -3.86646 | 2.20342 | -0.48067 |
| H | -6.29499 | 2.15938 | -0.40327 |
| C | -0.14613 | -0.72436 | -0.36301 |
| C | -7.88944 | -0.01199 | -0.18908 |
| N | -8.56481 | 1.17772 | -0.25548 |
| C | -6.46075 | 0.00576 | -0.25893 |
| C | -10.00859 | 3.60732 | 0.10259 |
| C | -10.72574 | 4.79529 | 0.07744 |
| C | -12.08627 | 4.78662 | -0.23223 |
| C | -12.72509 | 3.58223 | -0.51812 |
| C | -12.01345 | 2.38760 | -0.49090 |
| H | -12.64650 | 5.71719 | -0.25109 |
| H | -8.95252 | 3.60730 | 0.35139 |
| H | -10.22387 | 5.73140 | 0.30496 |
| H | -13.78152 | 3.57031 | -0.76979 |
| H | -12.52907 | 1.46562 | -0.74538 |
| C | -9.85928 | 1.15156 | -0.14072 |
| N | -10.52900 | -0.04142 | 0.01484 |
| C | -10.64595 | 2.39011 | -0.17974 |
| C | -11.93222 | -2.56883 | -0.09729 |
| C | -12.59914 | -3.78008 | 0.05178 |
| C | -11.91133 | -4.91155 | 0.48401 |
| C | -10.54646 | -4.83043 | 0.76254 |
| C | -9.87419 | -3.62575 | 0.61236 |
| H | -12.43636 | -5.85515 | 0.60301 |
| H | -12.48646 | -1.70846 | -0.46221 |
| H | -13.65925 | -3.83895 | -0.17687 |
| H | -10.00600 | -5.70906 | 1.10288 |
| H | -8.81477 | -3.55580 | 0.83587 |
| H | -11.50912 | -0.03584 | 0.26664 |
| H | 11.53358 | 0.06861 | 0.28698 |
| O | -9.55958 | 1.58366 | 3.16374 |
| O | -8.94411 | 0.55262 | 3.10933 |

**Supplementary Table S11. Atomistic coordinates of model of Act_2 on TT–CTP calculated using DFT method at the PBE0/6-31G* level of theory.**

| ***Atom*** | ***x (Å)*** | ***y (Å)*** | ***z (Å)*** |
| --- | --- | --- | --- |
| C | -10.24636 | -1.13385 | -0.21470 |
| N | -8.90960 | -1.15960 | -0.24715 |
| C | -10.96896 | -2.39983 | -0.46037 |
| C | -6.09510 | -1.14170 | -0.27616 |
| C | -4.70934 | -1.12275 | -0.29310 |
| C | -4.01081 | 0.07509 | -0.07101 |
| C | -4.74148 | 1.24881 | 0.16720 |
| C | -6.12799 | 1.22703 | 0.18145 |
| C | -2.55065 | 0.07255 | -0.08922 |
| N | -1.82219 | -1.02218 | -0.09358 |
| S | 0.93924 | -1.61836 | -0.12709 |
| H | -6.62091 | -2.07445 | -0.45178 |
| H | -4.15937 | -2.03818 | -0.48573 |
| H | -4.23422 | 2.19182 | 0.35734 |
| H | -6.67682 | 2.14343 | 0.37104 |
| C | -0.51480 | -0.68870 | -0.11727 |
| C | -8.30221 | 0.00830 | -0.01868 |
| N | -8.94007 | 1.15601 | 0.22856 |
| C | -6.82468 | 0.03175 | -0.03885 |
| C | -10.35467 | 3.53328 | 0.74434 |
| C | -11.06686 | 4.70122 | 0.99876 |
| C | -12.46203 | 4.68071 | 1.02175 |
| C | -13.14221 | 3.48481 | 0.78796 |
| C | -12.43278 | 2.31521 | 0.53344 |
| H | -13.01738 | 5.59379 | 1.22118 |
| H | -9.26966 | 3.55086 | 0.72688 |
| H | -10.53175 | 5.62980 | 1.18000 |
| H | -14.22882 | 3.46295 | 0.80423 |
| H | -12.96324 | 1.38592 | 0.35211 |
| C | -10.27553 | 1.08593 | 0.24068 |
| N | -10.97012 | -0.03594 | 0.02586 |
| C | -11.03100 | 2.32780 | 0.50946 |
| C | -12.37022 | -2.43512 | -0.42927 |
| C | -13.04885 | -3.62739 | -0.66153 |
| C | -12.33805 | -4.79844 | -0.92785 |
| C | -10.94318 | -4.77114 | -0.96040 |
| C | -10.26170 | -3.58053 | -0.72783 |
| H | -12.86945 | -5.72926 | -1.10968 |
| H | -12.92449 | -1.52523 | -0.22246 |
| H | -14.13535 | -3.64284 | -0.63465 |
| H | -10.38436 | -5.68012 | -1.16760 |
| H | -9.17689 | -3.56092 | -0.75326 |
| C | 9.54335 | 1.07098 | -0.54340 |
| N | 8.20658 | 1.10290 | -0.53161 |
| C | 10.26244 | 2.32495 | -0.85391 |
| C | 5.39114 | 1.09690 | -0.46043 |
| C | 4.00530 | 1.08107 | -0.43620 |
| C | 3.31145 | -0.11027 | -0.16913 |
| C | 4.04680 | -1.28016 | 0.07331 |
| C | 5.43303 | -1.26140 | 0.04662 |
| C | 1.85139 | -0.10651 | -0.14719 |
| N | 1.12274 | 0.98808 | -0.15232 |
| S | -1.63878 | 1.58395 | -0.12194 |
| H | 5.91336 | 2.02449 | -0.67064 |
| H | 3.45181 | 1.99377 | -0.63181 |
| H | 3.54381 | -2.21747 | 0.29922 |
| H | 5.98572 | -2.17453 | 0.24054 |
| C | -0.18473 | 0.65437 | -0.13425 |
| C | 7.60301 | -0.05474 | -0.24762 |
| N | 8.24382 | -1.19914 | 0.00564 |
| C | 6.12516 | -0.07300 | -0.22003 |
| C | 9.66608 | -3.56238 | 0.56385 |
| C | 10.38168 | -4.72689 | 0.82446 |
| C | 11.77635 | -4.71835 | 0.77652 |
| C | 12.45247 | -3.53742 | 0.46706 |
| C | 11.73953 | -2.37125 | 0.20643 |
| H | 12.33443 | -5.62872 | 0.98069 |
| H | 8.58151 | -3.57030 | 0.60233 |
| H | 9.84948 | -5.64336 | 1.06599 |
| H | 13.53865 | -3.52463 | 0.42845 |
| H | 12.26697 | -1.45362 | -0.03354 |
| C | 9.57903 | -1.13401 | -0.02469 |
| N | 10.27083 | -0.02308 | -0.29735 |
| C | 10.33834 | -2.37203 | 0.25282 |
| C | 11.66375 | 2.34945 | -0.88961 |
| C | 12.33914 | 3.52970 | -1.18443 |
| C | 11.62489 | 4.69948 | -1.44709 |
| C | 10.22989 | 4.68301 | -1.41263 |
| C | 9.55175 | 3.50418 | -1.11831 |
| H | 12.15378 | 5.62102 | -1.67723 |
| H | 12.22035 | 1.44056 | -0.68471 |
| H | 13.42579 | 3.53675 | -1.20908 |
| H | 9.66835 | 5.59124 | -1.61572 |
| H | 8.46685 | 3.49285 | -1.09120 |
| O | 9.17686 | 0.36049 | 3.28151 |
| O | 7.96063 | 0.89366 | 3.25617 |

**Supplementary Table S12. Atomistic coordinates of model of Act_2 on TTH–CTP calculated using DFT method at the PBE0/6-31G* level of theory.**

| ***Atom*** | ***x (Å)*** | ***y (Å)*** | ***z (Å)*** |
| --- | --- | --- | --- |
| C | 9.86814 | 1.20802 | -0.65022 |
| N | 8.55388 | 1.19558 | -0.73867 |
| C | 10.58937 | 2.46948 | -0.51010 |
| C | 5.76191 | 1.16807 | -0.73263 |
| C | 4.38060 | 1.14591 | -0.71532 |
| C | 3.68660 | -0.07047 | -0.83017 |
| C | 4.41705 | -1.26069 | -0.97052 |
| C | 5.79998 | -1.23907 | -0.98669 |
| C | 2.22944 | -0.06197 | -0.79071 |
| N | 1.51792 | 1.03243 | -0.63544 |
| S | -1.22994 | 1.65487 | -0.45503 |
| H | 6.28680 | 2.11218 | -0.63976 |
| H | 3.82405 | 2.07021 | -0.60928 |
| H | 3.91094 | -2.21782 | -1.06448 |
| H | 6.35209 | -2.16614 | -1.09153 |
| C | 0.20958 | 0.71589 | -0.62746 |
| C | 7.95164 | -0.00037 | -0.85036 |
| N | 8.60141 | -1.17147 | -0.95767 |
| C | 6.49158 | -0.02451 | -0.86374 |
| C | 10.01678 | -3.60045 | -0.76746 |
| C | 10.71540 | -4.79688 | -0.83394 |
| C | 12.08495 | -4.79192 | -1.09604 |
| C | 12.75660 | -3.58612 | -1.29335 |
| C | 12.06770 | -2.38327 | -1.22167 |
| H | 12.63028 | -5.72974 | -1.15027 |
| H | 8.95285 | -3.59575 | -0.55725 |
| H | 10.19190 | -5.73526 | -0.67849 |
| H | 13.82009 | -3.58200 | -1.51094 |
| H | 12.61148 | -1.46165 | -1.40975 |
| C | 9.91616 | -1.14736 | -0.86731 |
| N | 10.55467 | 0.03627 | -0.67551 |
| C | 10.68933 | -2.38267 | -0.95431 |
| C | 11.95901 | 2.57972 | -0.79643 |
| C | 12.59907 | 3.80261 | -0.65005 |
| C | 11.88600 | 4.91884 | -0.21472 |
| C | 10.52434 | 4.81518 | 0.06775 |
| C | 9.87424 | 3.59915 | -0.08304 |
| H | 12.39269 | 5.87264 | -0.09886 |
| H | 12.52933 | 1.73248 | -1.16679 |
| H | 13.65593 | 3.88573 | -0.88365 |
| H | 9.96917 | 5.68390 | 0.40814 |
| H | 8.81683 | 3.50898 | 0.14095 |
| C | -9.78874 | -1.14115 | -0.27743 |
| N | -8.47931 | -1.12163 | -0.30272 |
| C | -10.50036 | -2.43079 | -0.35298 |
| C | -5.70671 | -1.05133 | -0.49183 |
| C | -4.32326 | -1.03938 | -0.56837 |
| C | -3.61957 | 0.17323 | -0.52745 |
| C | -4.34280 | 1.36985 | -0.41165 |
| C | -5.72490 | 1.35308 | -0.33359 |
| C | -2.16278 | 0.15978 | -0.59904 |
| N | -1.45164 | -0.93367 | -0.76022 |
| S | 1.29617 | -1.55431 | -0.93883 |
| H | -6.23970 | -1.99463 | -0.52311 |
| H | -3.77698 | -1.97211 | -0.65744 |
| H | -3.83166 | 2.32903 | -0.38283 |
| H | -6.26980 | 2.28563 | -0.24125 |
| C | -0.14212 | -0.61499 | -0.77413 |
| C | -7.90835 | 0.12420 | -0.27683 |
| N | -8.52639 | 1.26960 | -0.21862 |
| C | -6.42842 | 0.14267 | -0.37140 |
| C | -10.33416 | 3.70566 | -0.35209 |
| C | -10.96394 | 4.76964 | -0.98792 |
| C | -11.89446 | 4.53006 | -1.99872 |
| C | -12.18835 | 3.22200 | -2.37234 |
| C | -11.55966 | 2.15284 | -1.73654 |
| H | -12.38533 | 5.36234 | -2.49556 |
| H | -9.60837 | 3.89610 | 0.43270 |
| H | -10.72759 | 5.78836 | -0.69363 |
| H | -12.90611 | 3.02749 | -3.16422 |
| H | -11.78874 | 1.13820 | -2.04980 |
| C | -9.91636 | 1.25840 | -0.02674 |
| N | -10.51852 | -0.01098 | -0.21638 |
| C | -10.63428 | 2.39002 | -0.72028 |
| C | -11.81759 | -2.52229 | -0.82056 |
| C | -12.45157 | -3.75838 | -0.88049 |
| C | -11.78259 | -4.90982 | -0.47038 |
| C | -10.47052 | -4.82423 | -0.00673 |
| C | -9.82921 | -3.59321 | 0.04671 |
| H | -12.28242 | -5.87337 | -0.51493 |
| H | -12.35112 | -1.64247 | -1.16984 |
| H | -13.46926 | -3.82126 | -1.25433 |
| H | -9.94569 | -5.71896 | 0.31543 |
| H | -8.80931 | -3.51916 | 0.40882 |
| H | -11.52774 | -0.06743 | -0.14082 |
| H | 11.55700 | 0.04098 | -0.50671 |
| O | -10.15437 | 1.61624 | 1.48476 |
| O | -9.57528 | 0.75356 | 2.25701 |

**Supplementary Table S13. Atomistic coordinates of model of Act_3 on TT–CTP calculated using DFT method at the PBE0/6-31G* level of theory.**

| ***Atom*** | ***x (Å)*** | ***y (Å)*** | ***z (Å)*** |
| --- | --- | --- | --- |
| C | 10.26882 | 1.16289 | -0.21413 |
| N | 8.93204 | 1.18425 | -0.18271 |
| C | 10.97708 | 2.44638 | -0.40470 |
| C | 6.11692 | 1.14436 | -0.14843 |
| C | 4.73157 | 1.11640 | -0.12250 |
| C | 4.04621 | -0.09216 | 0.08383 |
| C | 4.79220 | -1.26663 | 0.26498 |
| C | 6.17815 | -1.23616 | 0.23625 |
| C | 2.58620 | -0.10010 | 0.10372 |
| N | 1.84788 | 0.98212 | -0.01086 |
| S | -0.91999 | 1.55743 | -0.04043 |
| H | 6.63102 | 2.08601 | -0.31080 |
| H | 4.17104 | 2.03440 | -0.26559 |
| H | 4.29832 | -2.22064 | 0.43397 |
| H | 6.73741 | -2.15494 | 0.37768 |
| C | 0.54304 | 0.64272 | 0.04254 |
| C | 8.33780 | 0.00037 | -0.00663 |
| N | 8.98917 | -1.15772 | 0.13612 |
| C | 6.86116 | -0.03066 | 0.02870 |
| C | 10.43323 | -3.55135 | 0.46477 |
| C | 11.15952 | -4.72966 | 0.60685 |
| C | 12.55247 | -4.70904 | 0.52439 |
| C | 13.21638 | -3.50228 | 0.29964 |
| C | 12.49300 | -2.32201 | 0.15920 |
| H | 13.11869 | -5.63045 | 0.63485 |
| H | 9.34999 | -3.56892 | 0.52863 |
| H | 10.63714 | -5.66655 | 0.78236 |
| H | 14.30108 | -3.48040 | 0.23363 |
| H | 13.01045 | -1.38410 | -0.01540 |
| C | 10.32346 | -1.08204 | 0.08991 |
| N | 11.00521 | 0.05476 | -0.08401 |
| C | 11.09335 | -2.33502 | 0.24010 |
| C | 12.37680 | 2.48088 | -0.47788 |
| C | 13.04182 | 3.68965 | -0.65854 |
| C | 12.31882 | 4.87841 | -0.76659 |
| C | 10.92547 | 4.85208 | -0.69351 |
| C | 10.25754 | 3.64465 | -0.51451 |
| H | 12.83957 | 5.82226 | -0.90733 |
| H | 12.94036 | 1.55720 | -0.39390 |
| H | 14.12718 | 3.70414 | -0.71513 |
| H | 10.35720 | 5.77490 | -0.77611 |
| H | 9.17395 | 3.62560 | -0.45800 |
| C | -9.60128 | -1.23860 | 0.34487 |
| N | -8.17728 | -1.21143 | 0.14771 |
| C | -10.25618 | -2.27331 | -0.57892 |
| C | -5.35583 | -1.17315 | 0.46650 |
| C | -3.96922 | -1.15056 | 0.43765 |
| C | -3.28087 | 0.04120 | 0.15684 |
| C | -4.02843 | 1.20167 | -0.09116 |
| C | -5.41622 | 1.17255 | -0.05197 |
| C | -1.82164 | 0.04650 | 0.13426 |
| N | -1.08188 | -1.03515 | 0.24842 |
| S | 1.68399 | -1.60791 | 0.28141 |
| H | -5.87153 | -2.10075 | 0.69314 |
| H | -3.40995 | -2.05792 | 0.64352 |
| H | -3.53451 | 2.14247 | -0.32525 |
| H | -5.97806 | 2.08077 | -0.24422 |
| C | 0.22319 | -0.69320 | 0.19649 |
| C | -7.60135 | -0.04331 | 0.25923 |
| N | -8.20398 | 1.16465 | 0.36339 |
| C | -6.10687 | -0.01485 | 0.22240 |
| C | -9.55958 | 3.60156 | -0.03001 |
| C | -10.21746 | 4.82976 | -0.07611 |
| C | -11.59281 | 4.89871 | 0.14646 |
| C | -12.30323 | 3.72747 | 0.41435 |
| C | -11.64422 | 2.50073 | 0.45494 |
| H | -12.10669 | 5.85649 | 0.11427 |
| H | -8.48888 | 3.55156 | -0.20222 |
| H | -9.65332 | 5.73534 | -0.28735 |
| H | -13.37454 | 3.76965 | 0.59697 |
| H | -12.20074 | 1.59345 | 0.66843 |
| C | -9.55424 | 1.10008 | 0.27198 |
| N | -10.28728 | 0.02035 | 0.17635 |
| C | -10.26311 | 2.41806 | 0.23128 |
| C | -11.57297 | -2.69538 | -0.34988 |
| C | -12.19298 | -3.60262 | -1.20753 |
| C | -11.50935 | -4.10167 | -2.31791 |
| C | -10.20243 | -3.68189 | -2.55909 |
| C | -9.58414 | -2.77542 | -1.69535 |
| H | -11.99286 | -4.80809 | -2.98862 |
| H | -12.12146 | -2.31220 | 0.50596 |
| H | -13.21441 | -3.91956 | -1.00873 |
| H | -9.65885 | -4.05852 | -3.42284 |
| H | -8.56619 | -2.45080 | -1.88938 |
| O | -9.39001 | -0.95169 | 2.73329 |
| O | -9.88891 | -1.78601 | 1.65171 |

**Supplementary Table S14. Atomistic coordinates of model of Act_3 on TTH–CTP calculated using DFT method at the PBE0/6-31G* level of theory.**

| ***Atom*** | ***x (Å)*** | ***y (Å)*** | ***z (Å)*** |
| --- | --- | --- | --- |
| C | 9.85084 | 1.22041 | -0.17279 |
| N | 8.53581 | 1.18840 | -0.23891 |
| C | 10.56174 | 2.49541 | -0.18244 |
| C | 5.74245 | 1.13667 | -0.24359 |
| C | 4.36146 | 1.10373 | -0.22285 |
| C | 3.67861 | -0.12387 | -0.17905 |
| C | 4.42113 | -1.31507 | -0.16364 |
| C | 5.80355 | -1.28260 | -0.18516 |
| C | 2.22183 | -0.12577 | -0.14348 |
| N | 1.49711 | 0.97107 | -0.13065 |
| S | -1.25973 | 1.58062 | -0.03920 |
| H | 6.25812 | 2.08988 | -0.27451 |
| H | 3.79595 | 2.02858 | -0.23766 |
| H | 3.92393 | -2.28085 | -0.13049 |
| H | 6.36468 | -2.21004 | -0.16962 |
| C | 0.19222 | 0.64365 | -0.08602 |
| C | 7.94324 | -0.01756 | -0.21773 |
| N | 8.60291 | -1.18795 | -0.19973 |
| C | 6.48399 | -0.05585 | -0.22162 |
| C | 10.02212 | -3.58212 | 0.16000 |
| C | 10.72451 | -4.77689 | 0.21359 |
| C | 12.10486 | -4.78465 | 0.01689 |
| C | 12.78315 | -3.59271 | -0.23445 |
| C | 12.08954 | -2.39162 | -0.28469 |
| H | 12.65368 | -5.72115 | 0.05668 |
| H | 8.94935 | -3.56800 | 0.31771 |
| H | 10.19511 | -5.70390 | 0.41150 |
| H | 13.85622 | -3.59824 | -0.39816 |
| H | 12.64547 | -1.48499 | -0.50555 |
| C | 9.91820 | -1.14455 | -0.12946 |
| N | 10.54813 | 0.05813 | -0.08407 |
| C | 10.69967 | -2.37738 | -0.08574 |
| C | 11.93284 | 2.58141 | -0.46921 |
| C | 12.56269 | 3.81823 | -0.47028 |
| C | 11.83755 | 4.97377 | -0.18243 |
| C | 10.47442 | 4.89405 | 0.10069 |
| C | 9.83493 | 3.66319 | 0.09687 |
| H | 12.33599 | 5.93885 | -0.18205 |
| H | 12.51456 | 1.70017 | -0.72374 |
| H | 13.62115 | 3.88047 | -0.70305 |
| H | 9.90976 | 5.79337 | 0.32692 |
| H | 8.77630 | 3.59240 | 0.32166 |
| C | -9.93029 | -1.09811 | 0.38149 |
| N | -8.50844 | -1.18330 | 0.33386 |
| C | -10.58388 | -2.43919 | 0.14610 |
| C | -5.71448 | -1.15212 | 0.21336 |
| C | -4.33076 | -1.14013 | 0.14282 |
| C | -3.63253 | 0.07562 | 0.06920 |
| C | -4.36664 | 1.27041 | 0.06511 |
| C | -5.75085 | 1.24857 | 0.13655 |
| C | -2.17673 | 0.06686 | 0.00646 |
| N | -1.45088 | -1.02924 | -0.01253 |
| S | 1.30425 | -1.63476 | -0.10325 |
| H | -6.24542 | -2.09559 | 0.27277 |
| H | -3.77765 | -2.07361 | 0.14754 |
| H | -3.86218 | 2.23228 | 0.00506 |
| H | -6.30866 | 2.17810 | 0.13388 |
| C | -0.14465 | -0.69843 | -0.06323 |
| C | -7.94438 | 0.02015 | 0.29277 |
| N | -8.53979 | 1.20886 | 0.30765 |
| C | -6.45117 | 0.03901 | 0.21189 |
| C | -10.32273 | 3.52485 | 0.82059 |
| C | -10.93375 | 4.75272 | 0.58844 |
| C | -11.85771 | 4.89054 | -0.44727 |
| C | -12.16598 | 3.79137 | -1.24396 |
| C | -11.56043 | 2.55790 | -1.00592 |
| H | -12.33198 | 5.85058 | -0.63276 |
| H | -9.59576 | 3.42268 | 1.62099 |
| H | -10.68569 | 5.60534 | 1.21513 |
| H | -12.87953 | 3.88994 | -2.05776 |
| H | -11.80846 | 1.71351 | -1.64206 |
| C | -9.96127 | 1.09855 | 0.33827 |
| N | -10.43110 | -0.02318 | -0.44426 |
| C | -10.63474 | 2.41473 | 0.02823 |
| C | -11.53797 | -2.64542 | -0.85107 |
| C | -12.12416 | -3.89982 | -1.01749 |
| C | -11.76834 | -4.95744 | -0.18526 |
| C | -10.81574 | -4.75683 | 0.81363 |
| C | -10.22419 | -3.50819 | 0.97404 |
| H | -12.22733 | -5.93390 | -0.31501 |
| H | -11.82385 | -1.83540 | -1.51548 |
| H | -12.85999 | -4.04719 | -1.80358 |
| H | -10.53022 | -5.57665 | 1.46754 |
| H | -9.47456 | -3.35716 | 1.74530 |
| H | -11.44989 | -0.03396 | -0.39612 |
| H | 11.55504 | 0.08982 | 0.04588 |
| O | -10.43385 | 0.75721 | 1.69977 |
| O | -10.38177 | -0.69966 | 1.74218 |

**Supplementary Table S15. Atomistic coordinates of model of *OOH on TT–CTP calculated using DFT method at the PBE0/6-31G* level of theory.**

| ***Atom*** | ***x (Å)*** | ***y (Å)*** | ***z (Å)*** |
| --- | --- | --- | --- |
| C | 9.87855 | 1.17560 | -0.25133 |
| N | 8.54261 | 1.19621 | -0.19568 |
| C | 10.58584 | 2.46763 | -0.37692 |
| C | 5.72947 | 1.15842 | -0.11488 |
| C | 4.34463 | 1.13109 | -0.07123 |
| C | 3.65920 | -0.08716 | 0.06569 |
| C | 4.40435 | -1.27225 | 0.15968 |
| C | 5.78997 | -1.24208 | 0.11478 |
| C | 2.19949 | -0.09309 | 0.10310 |
| N | 1.46153 | 0.98718 | -0.03012 |
| S | -1.30495 | 1.56642 | -0.04274 |
| H | 6.24357 | 2.10794 | -0.22173 |
| H | 3.78452 | 2.05758 | -0.14313 |
| H | 3.90988 | -2.23503 | 0.26576 |
| H | 6.34885 | -2.16912 | 0.18632 |
| C | 0.15704 | 0.65170 | 0.04925 |
| C | 7.94917 | 0.00455 | -0.07990 |
| N | 8.60070 | -1.16038 | -0.01509 |
| C | 6.47304 | -0.02680 | -0.02425 |
| C | 10.04405 | -3.57206 | 0.12846 |
| C | 10.77005 | -4.75773 | 0.18536 |
| C | 12.16245 | -4.73230 | 0.09533 |
| C | 12.82614 | -3.51343 | -0.05115 |
| C | 12.10302 | -2.32599 | -0.10709 |
| H | 12.72863 | -5.65929 | 0.13895 |
| H | 8.96118 | -3.59320 | 0.19788 |
| H | 10.24795 | -5.70409 | 0.30021 |
| H | 13.91043 | -3.48770 | -0.12215 |
| H | 12.62011 | -1.37866 | -0.22117 |
| C | 9.93414 | -1.08335 | -0.08079 |
| N | 10.61495 | 0.06100 | -0.20050 |
| C | 10.70390 | -2.34376 | -0.01843 |
| C | 11.98445 | 2.50556 | -0.46776 |
| C | 12.64867 | 3.72254 | -0.58518 |
| C | 11.92602 | 4.91612 | -0.61197 |
| C | 10.53376 | 4.88633 | -0.52157 |
| C | 9.86661 | 3.67079 | -0.40559 |
| H | 12.44618 | 5.86634 | -0.70321 |
| H | 12.54791 | 1.57823 | -0.44677 |
| H | 13.73316 | 3.73969 | -0.65590 |
| H | 9.96558 | 5.81268 | -0.54140 |
| H | 8.78384 | 3.64922 | -0.33563 |
| C | -9.97893 | -1.12854 | 0.70358 |
| N | -8.56384 | -1.15246 | 0.51621 |
| C | -10.62547 | -2.36530 | 0.08295 |
| C | -5.73927 | -1.10214 | 0.68681 |
| C | -4.35311 | -1.08886 | 0.63754 |
| C | -3.66548 | 0.06338 | 0.22281 |
| C | -4.41190 | 1.19372 | -0.13956 |
| C | -5.79897 | 1.17511 | -0.08156 |
| C | -2.20633 | 0.06269 | 0.18199 |
| N | -1.46799 | -1.01694 | 0.31935 |
| S | 1.29781 | -1.59357 | 0.33594 |
| H | -6.25373 | -1.99813 | 1.01826 |
| H | -3.79393 | -1.97131 | 0.93254 |
| H | -3.91756 | 2.09972 | -0.48324 |
| H | -6.35964 | 2.05890 | -0.36632 |
| C | -0.16283 | -0.67908 | 0.24374 |
| C | -7.98069 | 0.01465 | 0.39323 |
| N | -8.58290 | 1.21934 | 0.27336 |
| C | -6.48851 | 0.02749 | 0.32916 |
| C | -10.01124 | 3.66025 | 0.17819 |
| C | -10.67738 | 4.85970 | -0.06782 |
| C | -11.98548 | 4.84970 | -0.55211 |
| C | -12.62348 | 3.62996 | -0.78359 |
| C | -11.96105 | 2.43219 | -0.52550 |
| H | -12.50393 | 5.78492 | -0.74939 |
| H | -8.99159 | 3.67179 | 0.55066 |
| H | -10.17224 | 5.80436 | 0.11938 |
| H | -13.64099 | 3.61120 | -1.16677 |
| H | -12.46075 | 1.48502 | -0.70535 |
| C | -9.93090 | 1.14667 | 0.22683 |
| N | -10.66712 | 0.06935 | 0.36104 |
| C | -10.64506 | 2.43114 | -0.04429 |
| C | -11.63428 | -2.25027 | -0.87505 |
| C | -12.19781 | -3.38827 | -1.45453 |
| C | -11.76259 | -4.65727 | -1.07626 |
| C | -10.75600 | -4.78091 | -0.11680 |
| C | -10.19009 | -3.64350 | 0.45558 |
| H | -12.20188 | -5.54434 | -1.52631 |
| H | -11.97523 | -1.26220 | -1.16933 |
| H | -12.97889 | -3.27999 | -2.20356 |
| H | -10.40910 | -5.76617 | 0.18588 |
| H | -9.40282 | -3.75136 | 1.19636 |
| O | -9.74088 | -0.34410 | 2.94843 |
| O | -10.26715 | -1.40373 | 2.14802 |
| H | -10.39273 | 0.36741 | 2.80524 |

**Supplementary Table S16. Atomistic coordinates of model of *OOH on TTH–CTP calculated using DFT method at the PBE0/6-31G* level of theory.**

| ***Atom*** | ***x (Å)*** | ***y (Å)*** | ***z (Å)*** |
| --- | --- | --- | --- |
| C | 9.82516 | 1.21745 | -0.12017 |
| N | 8.51105 | 1.18470 | -0.20819 |
| C | 10.53402 | 2.49311 | -0.09760 |
| C | 5.71797 | 1.13291 | -0.25455 |
| C | 4.33673 | 1.09967 | -0.24128 |
| C | 3.65449 | -0.12795 | -0.19626 |
| C | 4.39661 | -1.31908 | -0.17556 |
| C | 5.77925 | -1.28631 | -0.19068 |
| C | 2.19712 | -0.12967 | -0.16482 |
| N | 1.47019 | 0.96010 | -0.27435 |
| S | -1.28773 | 1.56808 | -0.24760 |
| H | 6.23372 | 2.08612 | -0.28370 |
| H | 3.77105 | 2.02437 | -0.25828 |
| H | 3.89956 | -2.28515 | -0.15176 |
| H | 6.34046 | -2.21360 | -0.17333 |
| C | 0.16611 | 0.63608 | -0.19063 |
| C | 7.91885 | -0.02113 | -0.21202 |
| N | 8.57891 | -1.19197 | -0.21466 |
| C | 6.45921 | -0.05945 | -0.22550 |
| C | 10.03244 | -3.57635 | 0.21667 |
| C | 10.74698 | -4.76499 | 0.24495 |
| C | 12.10571 | -4.77037 | -0.06880 |
| C | 12.75103 | -3.58322 | -0.41271 |
| C | 12.04664 | -2.38730 | -0.43576 |
| H | 12.66306 | -5.70247 | -0.04945 |
| H | 8.97689 | -3.56268 | 0.46557 |
| H | 10.24423 | -5.68899 | 0.51383 |
| H | 13.80527 | -3.58924 | -0.67136 |
| H | 12.56536 | -1.48191 | -0.73876 |
| C | 9.89298 | -1.14704 | -0.12910 |
| N | 10.52147 | 0.05385 | -0.03939 |
| C | 10.67965 | -2.37656 | -0.11663 |
| C | 11.90868 | 2.58543 | -0.36521 |
| C | 12.53644 | 3.82299 | -0.33885 |
| C | 11.80564 | 4.97286 | -0.04266 |
| C | 10.43908 | 4.88688 | 0.22162 |
| C | 9.80171 | 3.65535 | 0.19061 |
| H | 12.30241 | 5.93856 | -0.02114 |
| H | 12.49440 | 1.70878 | -0.62610 |
| H | 13.59766 | 3.89045 | -0.55718 |
| H | 9.87017 | 5.78183 | 0.45438 |
| H | 8.74041 | 3.57955 | 0.40081 |
| C | -9.92017 | -1.09313 | 0.89028 |
| N | -8.51398 | -1.08840 | 0.66378 |
| C | -10.56560 | -2.36216 | 0.35388 |
| C | -5.71678 | -1.11006 | 0.48584 |
| C | -4.33628 | -1.09963 | 0.39311 |
| C | -3.65539 | 0.07764 | 0.04190 |
| C | -4.40136 | 1.23394 | -0.22045 |
| C | -5.78508 | 1.21809 | -0.12554 |
| C | -2.19879 | 0.06787 | -0.03087 |
| N | -1.47150 | -1.02154 | 0.07566 |
| S | 1.28504 | -1.62682 | 0.05085 |
| H | -6.23079 | -2.02286 | 0.76555 |
| H | -3.77133 | -2.00186 | 0.60169 |
| H | -3.90998 | 2.15977 | -0.50974 |
| H | -6.34921 | 2.12045 | -0.33087 |
| C | -0.16642 | -0.69599 | -0.01447 |
| C | -7.94531 | 0.03595 | 0.35239 |
| N | -8.55955 | 1.23727 | 0.09195 |
| C | -6.46395 | 0.04803 | 0.23175 |
| C | -9.98951 | 3.68440 | -0.01579 |
| C | -10.66835 | 4.86557 | -0.28877 |
| C | -11.98408 | 4.82619 | -0.74793 |
| C | -12.61794 | 3.59927 | -0.93280 |
| C | -11.94569 | 2.41441 | -0.65239 |
| H | -12.51356 | 5.74987 | -0.96455 |
| H | -8.96601 | 3.70835 | 0.34317 |
| H | -10.16971 | 5.81931 | -0.14131 |
| H | -13.63861 | 3.56161 | -1.30211 |
| H | -12.45464 | 1.47049 | -0.82885 |
| C | -9.86989 | 1.21438 | 0.11877 |
| N | -10.56045 | 0.09306 | 0.38447 |
| C | -10.62402 | 2.44812 | -0.18884 |
| C | -11.39331 | -2.33616 | -0.76806 |
| C | -11.94911 | -3.51729 | -1.25697 |
| C | -11.67917 | -4.73043 | -0.63014 |
| C | -10.84446 | -4.76075 | 0.48645 |
| C | -10.28772 | -3.58388 | 0.97592 |
| H | -12.11283 | -5.65050 | -1.01229 |
| H | -11.60086 | -1.39795 | -1.27444 |
| H | -12.59174 | -3.48530 | -2.13245 |
| H | -10.62536 | -5.70430 | 0.97875 |
| H | -9.63526 | -3.61355 | 1.84280 |
| H | -11.56901 | 0.13161 | 0.46771 |
| H | 11.52430 | 0.07982 | 0.12407 |
| O | -9.55917 | -0.08899 | 2.96978 |
| O | -10.18630 | -1.18700 | 2.30985 |
| H | -10.30018 | 0.53642 | 3.05980 |

**Supplementary Table S17. Atomistic coordinates of model of *HOOH on TT–CTP calculated using DFT method at the PBE0/6-31G* level of theory.**

| ***Atom*** | ***x (Å)*** | ***y (Å)*** | ***z (Å)*** |
| --- | --- | --- | --- |
| O | -9.82560 | 2.10260 | 3.20008 |
| O | -9.27179 | 0.82073 | 3.50051 |
| H | -9.03220 | 2.66439 | 3.15240 |
| H | -9.21154 | 0.41149 | 2.61906 |
| C | 9.87477 | 1.14146 | 0.17711 |
| N | 8.53749 | 1.16246 | 0.16558 |
| C | 10.58449 | 2.41092 | 0.44128 |
| C | 5.72202 | 1.12828 | 0.12426 |
| C | 4.33632 | 1.10063 | 0.10613 |
| C | 3.65127 | -0.10050 | -0.13896 |
| C | 4.39527 | -1.26790 | -0.36642 |
| C | 5.78144 | -1.23759 | -0.34498 |
| C | 2.19102 | -0.10942 | -0.15146 |
| N | 1.45337 | 0.97909 | -0.15779 |
| S | -1.31337 | 1.55167 | -0.16709 |
| H | 6.23727 | 2.06317 | 0.31876 |
| H | 3.77603 | 2.01144 | 0.29079 |
| H | 3.89909 | -2.21277 | -0.57510 |
| H | 6.34057 | -2.14959 | -0.52529 |
| C | 0.14860 | 0.63446 | -0.15734 |
| C | 7.94219 | -0.00887 | -0.07656 |
| N | 8.59196 | -1.15500 | -0.29876 |
| C | 6.46477 | -0.03951 | -0.09966 |
| C | 10.03125 | -3.52622 | -0.77230 |
| C | 10.75552 | -4.69251 | -0.99863 |
| C | 12.15037 | -4.66987 | -0.96180 |
| C | 12.81808 | -3.47328 | -0.69751 |
| C | 12.09657 | -2.30517 | -0.47169 |
| H | 12.71512 | -5.58179 | -1.13878 |
| H | 8.94650 | -3.54548 | -0.80079 |
| H | 10.23006 | -5.62155 | -1.20416 |
| H | 13.90434 | -3.44981 | -0.66717 |
| H | 12.61732 | -1.37537 | -0.26637 |
| C | 9.92684 | -1.07999 | -0.26796 |
| N | 10.60995 | 0.04516 | -0.03451 |
| C | 10.69507 | -2.32006 | -0.50714 |
| C | 11.98591 | 2.45292 | 0.44642 |
| C | 12.65258 | 3.64832 | 0.69647 |
| C | 11.92948 | 4.81572 | 0.94514 |
| C | 10.53438 | 4.78173 | 0.94159 |
| C | 9.86485 | 3.58802 | 0.69092 |
| H | 12.45158 | 5.74894 | 1.14112 |
| H | 12.54956 | 1.54578 | 0.25337 |
| H | 13.73933 | 3.66905 | 0.69743 |
| H | 9.96611 | 5.68786 | 1.13507 |
| H | 8.77983 | 3.56308 | 0.68863 |
| C | -9.89512 | -1.22838 | 0.12495 |
| N | -8.55759 | -1.24718 | 0.12228 |
| C | -10.60417 | -2.49874 | 0.38561 |
| C | -5.74322 | -1.20033 | 0.13399 |
| C | -4.35753 | -1.17161 | 0.12152 |
| C | -3.67275 | 0.02265 | -0.15565 |
| C | -4.41668 | 1.18223 | -0.42017 |
| C | -5.80295 | 1.15104 | -0.40393 |
| C | -2.21250 | 0.03212 | -0.16293 |
| N | -1.47483 | -1.05626 | -0.15030 |
| S | 1.29156 | -1.62880 | -0.13849 |
| H | -6.25856 | -2.12981 | 0.35262 |
| H | -3.79710 | -2.07584 | 0.33576 |
| H | -3.92002 | 2.12074 | -0.65457 |
| H | -6.36227 | 2.05649 | -0.61434 |
| C | -0.17015 | -0.71146 | -0.14879 |
| C | -7.96350 | -0.07387 | -0.11534 |
| N | -8.61424 | 1.06937 | -0.34979 |
| C | -6.48599 | -0.04043 | -0.12759 |
| C | -10.05715 | 3.45212 | -0.75131 |
| C | -10.78169 | 4.61439 | -0.99665 |
| C | -12.17375 | 4.56752 | -1.08375 |
| C | -12.83848 | 3.35103 | -0.92311 |
| C | -12.11678 | 2.18725 | -0.67636 |
| H | -12.73861 | 5.47612 | -1.27674 |
| H | -8.97451 | 3.48982 | -0.68447 |
| H | -10.25885 | 5.55927 | -1.12006 |
| H | -13.92240 | 3.30835 | -0.99154 |
| H | -12.63476 | 1.24167 | -0.55226 |
| C | -9.94932 | 0.99073 | -0.32975 |
| N | -10.63189 | -0.13528 | -0.09860 |
| C | -10.71819 | 2.22654 | -0.58822 |
| C | -11.99752 | -2.51454 | 0.54016 |
| C | -12.66356 | -3.71104 | 0.78672 |
| C | -11.94813 | -4.90577 | 0.87833 |
| C | -10.56120 | -4.89791 | 0.72352 |
| C | -9.89186 | -3.70251 | 0.48039 |
| H | -12.46989 | -5.84006 | 1.07003 |
| H | -12.55468 | -1.58586 | 0.46907 |
| H | -13.74355 | -3.71112 | 0.90911 |
| H | -9.99927 | -5.82578 | 0.79139 |
| H | -8.81329 | -3.69757 | 0.35984 |

**Supplementary Table S18. Atomistic coordinates of model of *HOOH on TTH–CTP calculated using DFT method at the PBE0/6-31G* level of theory.**

| ***Atom*** | ***x (Å)*** | ***y (Å)*** | ***z (Å)*** |
| --- | --- | --- | --- |
| O | -9.05741 | 1.72488 | 2.95845 |
| O | -8.64847 | 0.35583 | 2.95663 |
| H | -8.91436 | 1.97260 | 3.88809 |
| H | -7.72722 | 0.42479 | 2.65191 |
| C | 9.76747 | 1.20580 | 0.06010 |
| N | 8.45625 | 1.17678 | -0.06415 |
| C | 10.47187 | 2.47871 | 0.18432 |
| C | 5.67024 | 1.12458 | -0.22237 |
| C | 4.28987 | 1.09121 | -0.27607 |
| C | 3.61079 | -0.13618 | -0.35325 |
| C | 4.35401 | -1.32609 | -0.38397 |
| C | 5.73594 | -1.29293 | -0.33085 |
| C | 2.15320 | -0.13892 | -0.38871 |
| N | 1.42962 | 0.95865 | -0.39622 |
| S | -1.32502 | 1.56662 | -0.41567 |
| H | 6.18404 | 2.07708 | -0.15888 |
| H | 3.72296 | 2.01500 | -0.25352 |
| H | 3.85909 | -2.29138 | -0.44928 |
| H | 6.29858 | -2.21932 | -0.35221 |
| C | 0.12447 | 0.63035 | -0.41202 |
| C | 7.87107 | -0.02849 | -0.16398 |
| N | 8.53351 | -1.19593 | -0.20002 |
| C | 6.41242 | -0.06695 | -0.24520 |
| C | 9.94616 | -3.60865 | 0.02152 |
| C | 10.65154 | -4.80275 | 0.02781 |
| C | 12.04254 | -4.79211 | -0.06682 |
| C | 12.72841 | -3.58237 | -0.16839 |
| C | 12.03162 | -2.38218 | -0.17109 |
| H | 12.59419 | -5.72777 | -0.06354 |
| H | 8.86464 | -3.60904 | 0.09991 |
| H | 10.11605 | -5.74368 | 0.10870 |
| H | 13.81070 | -3.57284 | -0.25111 |
| H | 12.59793 | -1.46075 | -0.27242 |
| C | 9.84514 | -1.15602 | -0.06931 |
| N | 10.46588 | 0.04167 | 0.08609 |
| C | 10.63060 | -2.38656 | -0.07416 |
| C | 11.84710 | 2.59785 | -0.07008 |
| C | 12.46946 | 3.83158 | 0.06084 |
| C | 11.73335 | 4.94997 | 0.44972 |
| C | 10.36620 | 4.83743 | 0.70027 |
| C | 9.73362 | 3.61051 | 0.56369 |
| H | 12.22637 | 5.91222 | 0.55401 |
| H | 12.43697 | 1.74906 | -0.40452 |
| H | 13.53087 | 3.92112 | -0.14851 |
| H | 9.79285 | 5.70786 | 1.00421 |
| H | 8.67182 | 3.51386 | 0.76260 |
| C | -9.86454 | -1.24774 | -0.00341 |
| N | -8.55555 | -1.23108 | -0.15186 |
| C | -10.57733 | -2.51206 | 0.15285 |
| C | -5.76368 | -1.19544 | -0.27560 |
| C | -4.38247 | -1.16718 | -0.31154 |
| C | -3.69852 | 0.05629 | -0.40659 |
| C | -4.43748 | 1.24729 | -0.47443 |
| C | -5.81993 | 1.21947 | -0.43849 |
| C | -2.24055 | 0.05618 | -0.42143 |
| N | -1.51744 | -1.04172 | -0.42155 |
| S | 1.23818 | -1.64962 | -0.40311 |
| H | -6.28139 | -2.14477 | -0.19721 |
| H | -3.81885 | -2.09187 | -0.26006 |
| H | -3.93904 | 2.20930 | -0.55840 |
| H | -6.37920 | 2.14667 | -0.48921 |
| C | -0.21190 | -0.71377 | -0.41685 |
| C | -7.96149 | -0.03200 | -0.26731 |
| N | -8.61693 | 1.13827 | -0.33941 |
| C | -6.50160 | -0.00233 | -0.33444 |
| C | -10.04297 | 3.55450 | 0.02406 |
| C | -10.75181 | 4.74682 | 0.00880 |
| C | -12.12069 | 4.74196 | -0.25764 |
| C | -12.78262 | 3.54079 | -0.50873 |
| C | -12.08391 | 2.34141 | -0.48742 |
| H | -12.67347 | 5.67688 | -0.27228 |
| H | -8.98044 | 3.54771 | 0.24147 |
| H | -10.23716 | 5.68181 | 0.20825 |
| H | -13.84560 | 3.53806 | -0.72890 |
| H | -12.61573 | 1.42174 | -0.71582 |
| C | -9.92534 | 1.10921 | -0.18614 |
| N | -10.55357 | -0.07778 | 0.01335 |
| C | -10.70624 | 2.34202 | -0.21910 |
| C | -11.95491 | -2.62386 | -0.09160 |
| C | -12.58745 | -3.84892 | 0.06873 |
| C | -11.85883 | -4.96529 | 0.47720 |
| C | -10.48936 | -4.85975 | 0.71825 |
| C | -9.84669 | -3.64165 | 0.55259 |
| H | -12.35967 | -5.92071 | 0.60462 |
| H | -12.53738 | -1.77553 | -0.43997 |
| H | -13.65075 | -3.93361 | -0.13296 |
| H | -9.92231 | -5.72863 | 1.03806 |
| H | -8.78324 | -3.54975 | 0.74508 |
| H | -11.54670 | -0.08424 | 0.23143 |
| H | 11.46662 | 0.06581 | 0.25809 |

**Supplementary Table S19. Atomistic coordinates of model of H_2_O on TT–CTP calculated using DFT method at the PBE0/6-31G* level of theory.**

| ***Atom*** | ***x (Å)*** | ***y (Å)*** | ***z (Å)*** |
| --- | --- | --- | --- |
| H | 5.98886 | 7.66728 | 6.37841 |
| H | 4.46098 | 7.66736 | 6.37841 |
| O | 5.22495 | 8.26239 | 6.37841 |

**Supplementary Table S20. Atomistic coordinates of model of H_2_O on TTH–CTP calculated using DFT method at the PBE0/6-31G* level of theory.**

| ***Atom*** | ***x (Å)*** | ***y (Å)*** | ***z (Å)*** |
| --- | --- | --- | --- |
| H | 5.98052 | 7.66441 | 6.37841 |
| H | 4.46932 | 7.66448 | 6.37841 |
| O | 5.22495 | 8.26813 | 6.37841 |

**Supplementary Table S21. Atomistic coordinates of model of H_3_O^+^ on TT–CTP calculated using DFT method at the PBE0/6-31G* level of theory.**

| ***Atom*** | ***x (Å)*** | ***y (Å)*** | ***z (Å)*** |
| --- | --- | --- | --- |
| H | 5.51908 | 8.27447 | 6.36394 |
| H | 4.73607 | 6.89632 | 6.36397 |
| O | 4.73168 | 7.80991 | 6.72337 |
| H | 3.93688 | 8.26476 | 6.36791 |

**Supplementary Table S22. Atomistic coordinates of model of H_3_O^+^ on TTH–CTP calculated using DFT method at the PBE0/6-31G* level of theory.**

| ***Atom*** | ***x (Å)*** | ***y (Å)*** | ***z (Å)*** |
| --- | --- | --- | --- |
| H | 5.51850 | 8.27413 | 6.36346 |
| H | 4.73606 | 6.89698 | 6.36349 |
| O | 4.73169 | 7.80992 | 6.72480 |
| H | 3.93747 | 8.26443 | 6.36744 |

**Supplementary Table S23. Atomistic coordinates of model of O_2_ on TT–CTP calculated using DFT method at the PBE0/6-31G* level of theory.**

| ***Atom*** | ***x (Å)*** | ***y (Å)*** | ***z (Å)*** |
| --- | --- | --- | --- |
| O | 4.38794 | 9.38616 | 4.32438 |
| O | 5.59000 | 9.38616 | 4.32438 |

**Supplementary Table S24. Atomistic coordinates of model of O_2_ on TTH–CTP calculated using DFT method at the PBE0/6-31G* level of theory.**

| ***Atom*** | ***x (Å)*** | ***y (Å)*** | ***z (Å)*** |
| --- | --- | --- | --- |
| O | 4.38803 | 9.38616 | 4.32438 |
| O | 5.58991 | 9.38616 | 4.32438 |

**Supplementary Table S25. Atomistic coordinates of model of H_2_O_2_ on TT–CTP calculated using DFT method at the PBE0/6-31G* level of theory.**

| ***Atom*** | ***x (Å)*** | ***y (Å)*** | ***z (Å)*** |
| --- | --- | --- | --- |
| H | 4.49899 | 8.62312 | 6.11310 |
| H | 6.40815 | 7.29965 | 6.11341 |
| O | 4.74033 | 7.92523 | 6.74699 |
| O | 6.16687 | 7.99792 | 6.74689 |

**Supplementary Table S26. Atomistic coordinates of model of H_2_O_2_ on TTH–CTP calculated using DFT method at the PBE0/6-31G* level of theory.**

| ***Atom*** | ***x (Å)*** | ***y (Å)*** | ***z (Å)*** |
| --- | --- | --- | --- |
| H | 4.48388 | 8.64820 | 6.13306 |
| H | 6.42321 | 7.27455 | 6.13336 |
| O | 4.71824 | 7.90453 | 6.72700 |
| O | 6.18901 | 8.01864 | 6.72697 |

**Section 4: Comparison of the E_b_ values of organic/polymer semiconductors**

**Supplementary Table S27. E_b_ values of different organic semiconductor materials.**

| Material | Status | E_b_ (meV) | Ref. |
| --- | --- | --- | --- |
| 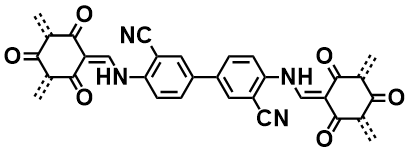 | powder | 31.2 | S27 |
| 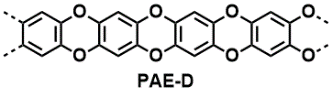 | powder | 42 | S28 |
| 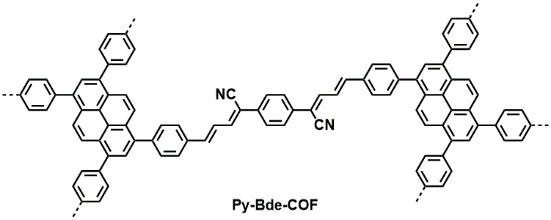 | powder | 44.4 | S29 |
| **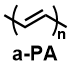** | crystal | 50 | S30 |
| **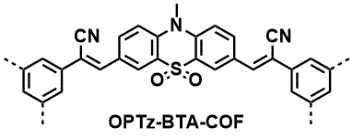** | powder | 53.9 | S31 |
| **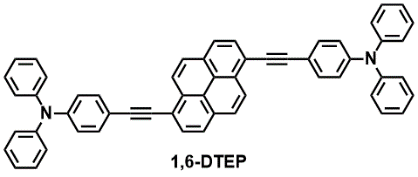** | crystal | 59.2 | S32 |
| **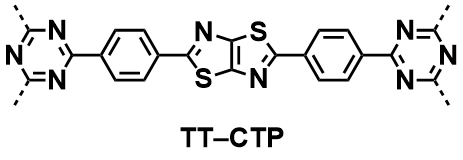** | thin film | 74.5 | this work |
| 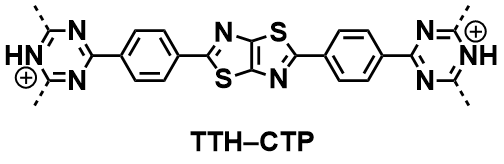 | thin film | 83.2 | this work |
| 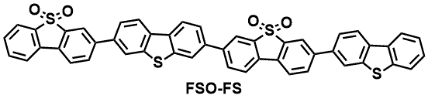 | powder | 88 | S33 |
| 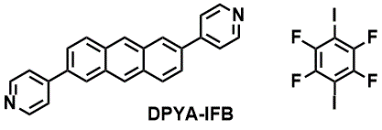 | cocrystal | 95.9 | S34 |
| 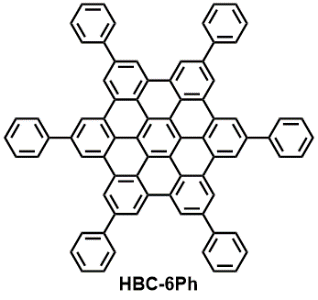 | thin film | 110 | S35 |
| 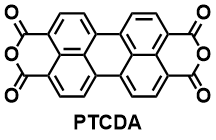 | thin film | 150 | S36 |
| 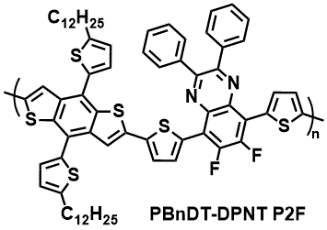 | thin film | 180 | S37 |
| 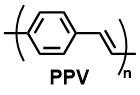 | crystal | 200 | S38 |
| 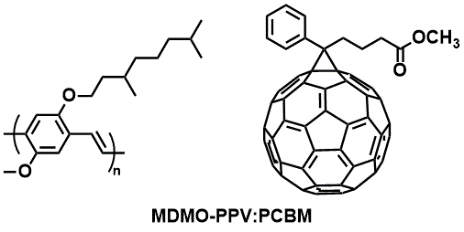 | thin film | 203 | S39 |
| 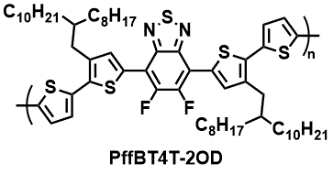 | thin film | 300 | S40 |
| 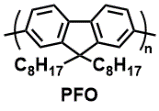 | thin film | 300 | S41 |
| 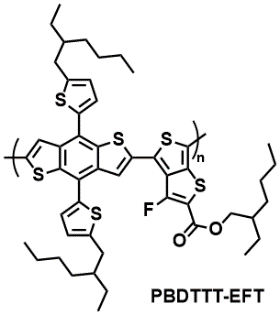 | thin film | 360 | S40 |
| 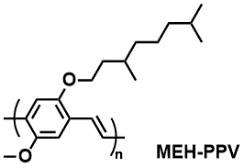 | thin film | 360 | S41 |
| 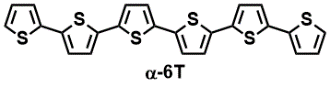 | thin film | 400 | S41 |
| 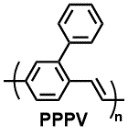 | thin film | 400 | S42 |
| 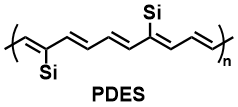 | thin film | 500 | S43 |
| 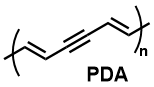 | thin film | 500 | S44 |
| 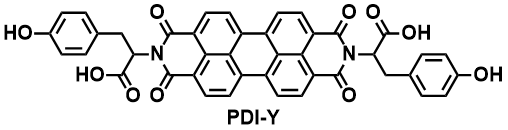 | thin film | 510 | S45 |
| 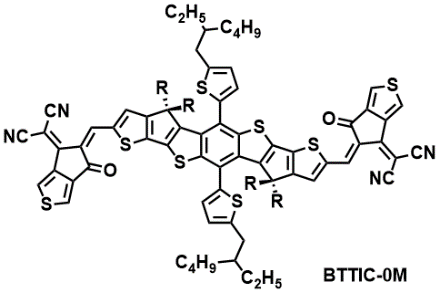 | crystal | 540 | S46 |
| 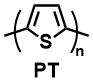 | thin film | 600 | S43 |
| 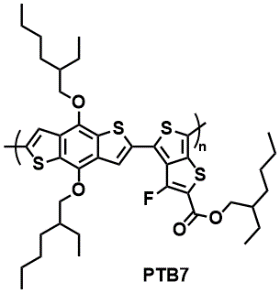 | thin film | 710 | S47 |
| 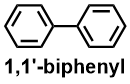 | crystal | 720 | S48 |
| 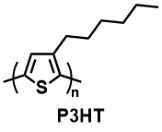 | thin film | 730 | S47 |
| 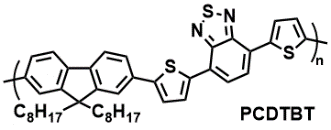 | thin film | 740 | S47 |
| 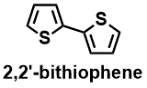 | crystal | 780 | S49 |
| 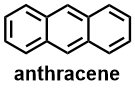 | crystal | 1000 | S50 |
| 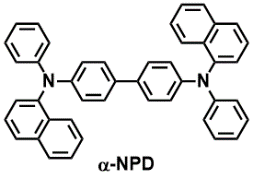 | thin film | 1000 | S51 |

**Section 5: Transient kinetics of the CTPs**

**Supplementary Table S28. Rate constants derived from the fitting on a 660-nm kinetic curve for TT–CTP film and a 680-nm kinetic curve for TTH–CTP film, respectively.**

| Simple | τ_1_ (ps) | τ_2_ (ps) | τ_3_ (ps) |
| --- | --- | --- | --- |
| TT–CTP | 0.62 | 12.8 | 640 |
| TTH–CTP | 0.38 | 9.3 | 340 |

**Supplementary References**

1. Frisch, M. J. *et al.* Gaussian 16, 2016.
2. Perdew, J. P. *et al.* Restoring the density-gradient expansion for exchange in solids and surfaces. *Phys. Rev. Lett.* **100**, 136406 (2008).
3. Perdew, J. P., Burke, K. & Ernzerhof. M. Generalized gradient approximation made simple. *Phys. Rev. Lett.* **77**, 3865 (1996).
4. Hariharan, P. C. & Pople. J. A. The influence of polarization functions on molecular orbital hydrogenation energies. *Theoreti. chim. Acta* **28**, 213 (1973).
5. Hehre, W. J., Ditchfield, R. & Pople, J. A. Self-consistent molecular orbital methods. XII. Further extensions of Gaussian-type basis sets for use in molecular orbital studies of organic molecules. *J. Chem. Phys.* **56**, 2257 (1972).
6. Marenich, A. V., Cramer, C. J. & Truhlar, D. G. Universal solvation model based on solute electron density and on a continuum model of the solvent defined by the bulk dielectric constant and atomic surface tensions. *J. Phys. Chem. B* **113**, 6378 (2009).
7. Lu, T. & Chen, F. Multiwfn: a multifunctional wavefunction analyzer. *J. Comput. Chem.* **33**, 580 (2012).
8. Humphrey, W., Dalke, A. & Schulten, K. VMD: visual molecular dynamics. *J. Mol. Graphics* **14**, 33 (1996).
9. Zhang, J. & Lu, T. Efficient evaluation of electrostatic potential with computerized optimized code. *Phys. Chem. Chem. Phys.* **23**, 20323 (2021).
10. CYLview, 1.0b; Legault, C. Y., Université de Sherbrooke, 2009 (http://www.cylview.org).
11. Teranishi, M., Naya, S.-i. & Tada, H. In situ liquid phase synthesis of hydrogen peroxide from molecular oxygen using gold nanoparticle-loaded titanium(IV) dioxide photocatalyst. *J. Am. Chem. Soc.* **132**, 7850 (2010).
12. Shiraishi, Y. *et al.* Sunlight-driven hydrogen peroxide production from water and molecular oxygen by metal-free photocatalysts. *Angew. Chem. Int. Ed.* **53**, 13454 (2014).
13. Ma, R. *et al.* Solid acids accelerate the photocatalytic hydrogen peroxide synthesis over a hybrid catalyst of titania nanotube with carbon dot. *Appl. Catal. B* **244**, 594 (2019).
14. Kofuji, Y. *et al.* Carbon nitride–aromatic diimide–graphene nanohybrids: metal-free photocatalysts for solar-to-hydrogen peroxide energy conversion with 0.2% efficiency. *J. Am. Chem. Soc.* **138**, 10019 (2016).
15. Moon, G.-h., Fujitsuka, M., Kim, S., Majima, T., Wang, X. & Choi, W. Eco-friendly photochemical production of H_2_O_2_ through O_2_ reduction over carbon nitride frameworks incorporated with multiple heteroelements. *ACS Catal.* **7**, 2886 (2017).
16. Wei, Z., Liu, M., Zhang, Z., Yao, W., Tan, H. & Zhu, Y. Efficient visible-light-driven selective oxygen reduction to hydrogen peroxide by oxygen-enriched graphitic carbon nitride polymers. *Energy Environ. Sci.* **11**, 2581 (2018).
17. Zhu, Z., Pan, H., Murugananthan, M., Gong, J. & Zhang, Y. Visible light-driven photocatalytically active g-C_3_N_4_ material for enhanced generation of H_2_O_2_. *Appl. Catal. B* **232**, 19 (2018).
18. Hirakawa, H., Shiota, S., Shiraishi, Y., Sakamoto, H., Ichikawa, S. & Hirai, T. Au nanoparticles supported on BiVO_4_: effective inorganic photocatalysts for H_2_O_2_ production from water and O_2_ under visible light. *ACS Catal.* **6**, 4976 (2016).
19. Xu, J. *et al.* Cd_3_(C_3_N_3_S_3_)_2_ coordination polymer/graphene nanoarchitectures for enhanced photocatalytic H_2_O_2_ production under visible light. *Sci. Bull.* **62**, 610 (2017).
20. Zhai, L. et al. Constructing synergistic triazine and acetylene cores in fully conjugated covalent organic frameworks for cascade photocatalytic H_2_O_2_ production. *Chem. Mater.* **34**, 5232 (2022).
21. Wu, C. *et al.* Polarization engineering of covalent triazine frameworks for highly efficient photosynthesis of hydrogen peroxide from molecular oxygen and water. *Adv. Mater.* **34**, 2110266 (2022).
22. Li, G. *et al.* Boosting exciton dissociation by regulating dielectric constant in covalent organic framework for photocatalysis. *Chem Catalysis* **2**, 1734–1747 (2022).
23. Wang, H., Yang, C., Chen, F., Zheng, G. & Han, Q. A crystalline partially fluorinated triazine covalent organic framework for efficient photosynthesis of hydrogen peroxide. *Angew. Chem. Int. Ed.* **61**, e202202328 (2022).
24. Yu, X. *et al.* Electronic tuning of covalent triazine framework nanoshells for highly efficient photocatalytic H_2_O_2_ production. *Adv. Sustainable Syst.* **5**, 2100184 (2021).
25. Kou, M. *et al.* Molecularly engineered covalent organic frameworks for hydrogen peroxide photosynthesis. *Angew. Chem. Int. Ed.* **61**, e202200413 (2022).
26. Chang, J.-N. *et al.* Oxidation-reduction molecular junction covalent organic frameworks for full reaction photosynthesis of H_2_O_2_. *Angew. Chem. Int. Ed.* **62**, e202218868 (2023).
27. Li, C., Liu, J., Li, H., Wu, K., Wang, J. & Yang, Q. Covalent organic frameworks with high quantum efficiency in sacrificial photocatalytic hydrogen evolution. *Nat. Commun.* **13**, 2357 (2022).
28. Lan, Z.-A. *et al.* A fully coplanar donor–acceptor polymeric semiconductor with promoted charge separation kinetics for photochemistry. *Angew. Chem. Int. Ed.* **60**, 16355 (2021).
29. Su, Y. *et al.* Multi-component synthesis of a buta-1,3-diene-linked covalent organic framework. *J. Am. Chem. Soc.* **144**, 18218 (2022).
30. Puschnig, P. & Ambrosch-Draxl, C. Suppression of electron-hole correlations in 3D polymer materials. *Phys. Rev. Lett.* **89**, 056405 (2002).
31. Wang, W. *et al.* Phenothiazine-based covalent organic frameworks with low exciton binding energies for photocatalysis. *Chem. Sci.* **13**, 8679 (2022).
32. Tao, J. *et al.* Organic UV-sensitive phototransistors based on distriphenylamineethynylpyrene derivatives with ultra-high detectivity approaching 10^18^. *Adv. Mater.* **32**, 1907791 (2020).
33. Lan, Z. A., Zhang, G., Chen, X., Zhang, Y., Zhang, K. A. I. & Wang*.* X. Reducing the exciton binding energy of donor-acceptor-based conjugated polymers to promote charge-induced reactions. *Angew. Chem. Int. Ed*. **58**, 10236 (2019).
34. Bolla, G. *et al.* Cocrystallization tailoring multiple radiative decay pathways for amplified spontaneous emission. *Angew. Chem. Int. Ed.* **60**, 281 (2021).
35. Zeng, C. *et al.* Electrochemical deposition of a single-crystalline nanorod polycyclic aromatic hydrocarbon film with efficient charge and exciton transport, *Angew. Chem. Int. Ed.* **61**, e202115389 (2022).
36. Shen, Z., Burrows, P. E., Forrest, S. R., Ziari, M. & Steier, W. H. Electroabsorption due to excitons in crystalline molecular thin films grown by organic molecular beam deposition. *Chem. Phys. Lett.* **236**, 129 (1995).
37. Yang, P., Yuan, M., Zeigler, D. F., Watkins, S. E., Lee, J. A. & Luscombe, C. K. Influence of fluorine substituents on the film dielectric constant and open-circuit voltage in organic photovoltaics. *J. Mater. Chem. C* **2**, 3278 (2014).
38. Ruini, A., Caldas, M. J., Bussi, G. & Molinari, E. Solid state effects on exciton states and optical properties of PPV. *Phys. Rev. Lett.* **88**, 206403 (2002).
39. Kern, J., Schwab, S., Deibel, C. & Dyakonov, V. Binding energy of singlet excitons and charge transfer complexes in MDMO-PPV:PCBM solar cells. *Phys. Status Solidi RRL* **5**, 364 (2011).
40. Cha, H. *et al.* An analysis of the factors determining the efficiency of photocurrent generation in polymer:nonfullerene acceptor solar cells. *Adv. Energy Mater.* **8**, 1801537 (2018).
41. Alvarado, S. F., Seidler, P. F., Lidzey, D. G. & Bradley, D. D. C. Direct determination of the exciton binding energy of conjugated polymers using a scanning tunneling microscope. *Phys. Rev. Lett.* **81**, 1082 (1998).
42. Kersting, R. *et al.* Ultrafast field-induced dissociation of excitons in conjugated polymers. *Phys. Rev. Lett.* **73**, 1440 (1994).
43. Liess, M. *et al.* Electroabsorption spectroscopy of luminescent and nonluminescent π-conjugated polymers. *Phys. Rev. B* **56**, 15712 (1997).
44. Weiser, G. Stark effect of one-dimensional Wannier excitons in polydiacetylene single crystals. *Phys. Rev. B* **45**, 14076 (1992).
45. Kim, Y.-O. *et al.* A multifunctional tyrosine-immobilized PAH molecule as a universal cathode interlayer enables high-efficiency inverted polymer solar cells. *Adv. Optical Mater.* **9**, 2101006 (2021).
46. Gao, W. *et al.* Regulating exciton bonding energy and bulk heterojunction morphology in organic solar cells via methyl-functionalized non-fullerene acceptors. *J. Mater. Chem. A* **7**, 6809 (2019).
47. Li, H.-W. *et al.* On the study of exciton binding energy with direct charge generation in photovoltaic polymers. *Adv. Electron. Mater.* **2**, 1600200 (2016).
48. Ambrosch-Draxl, C., Hummer, K., Sagmeister, S. & Puschnig, P. Excitonic effects in molecular crystals built up by small organic molecules. *Chem. Phys.* **325**, 3 (2006).
49. Mani, A., Schoonman, J. & Goossens. A. Photoluminescence study of sexithiophene thin films. *J. Phys. Chem. B* **109**, 4829 (2005).
50. Schweitzer, B. & Bässler, H. Excitons in conjugated polymers. *Synth. Met.* **109**, 1 (2000).
51. Hill, I. G., Kahn, A., Soos, Z. G. & Pascal, R. A. Charge-separation energy in films of π-conjugated organic molecules. *Chem. Phys. Lett.* **327**, 181 (2000).
