# Supplementary figures and images for: Efficient photocatalytic production of hydrogen peroxide using dispersible and photoactive porous polymers
Source: Nat Commun. 2023 Oct 28;14:6891. doi: 10.1038/s41467-023-42720-6 (PMC10613291; doi:10.1038/s41467-023-42720-6)

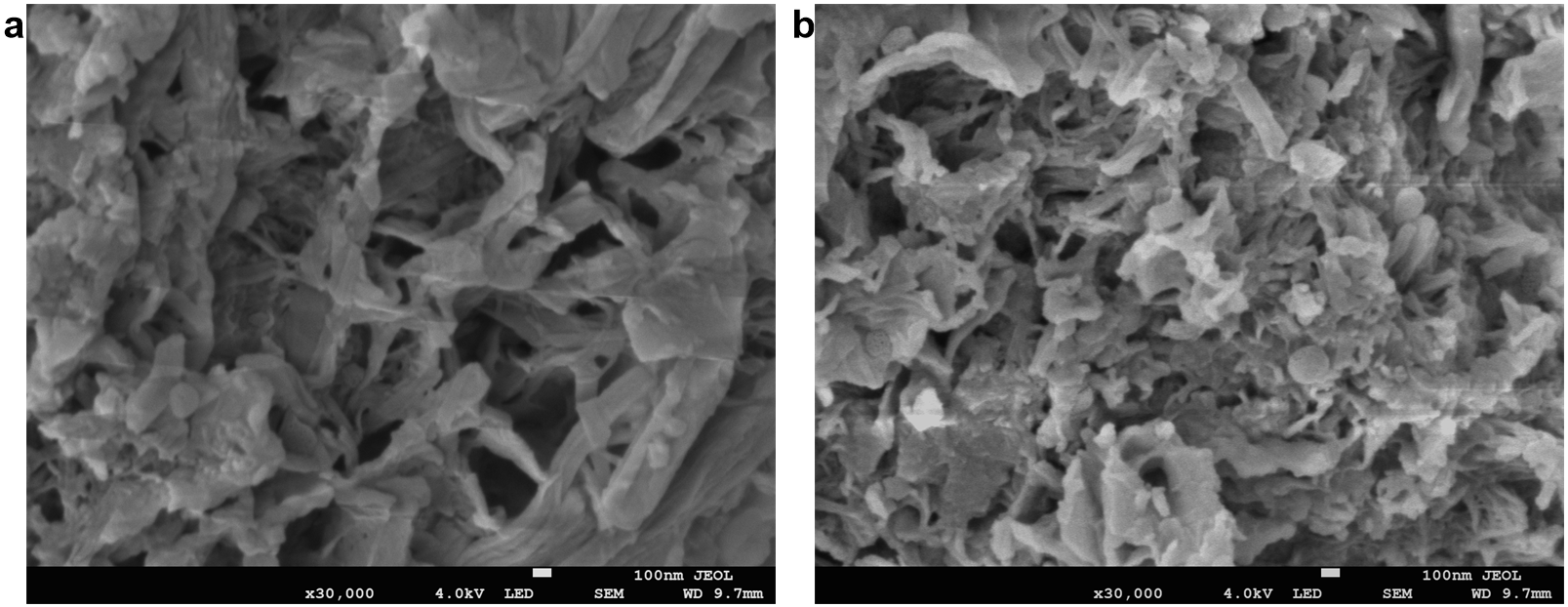

Supplement: Supplementary file 3 — Source Data [file 41467_2023_42720_MOESM3_ESM.zip › source data/Fig S13.png]

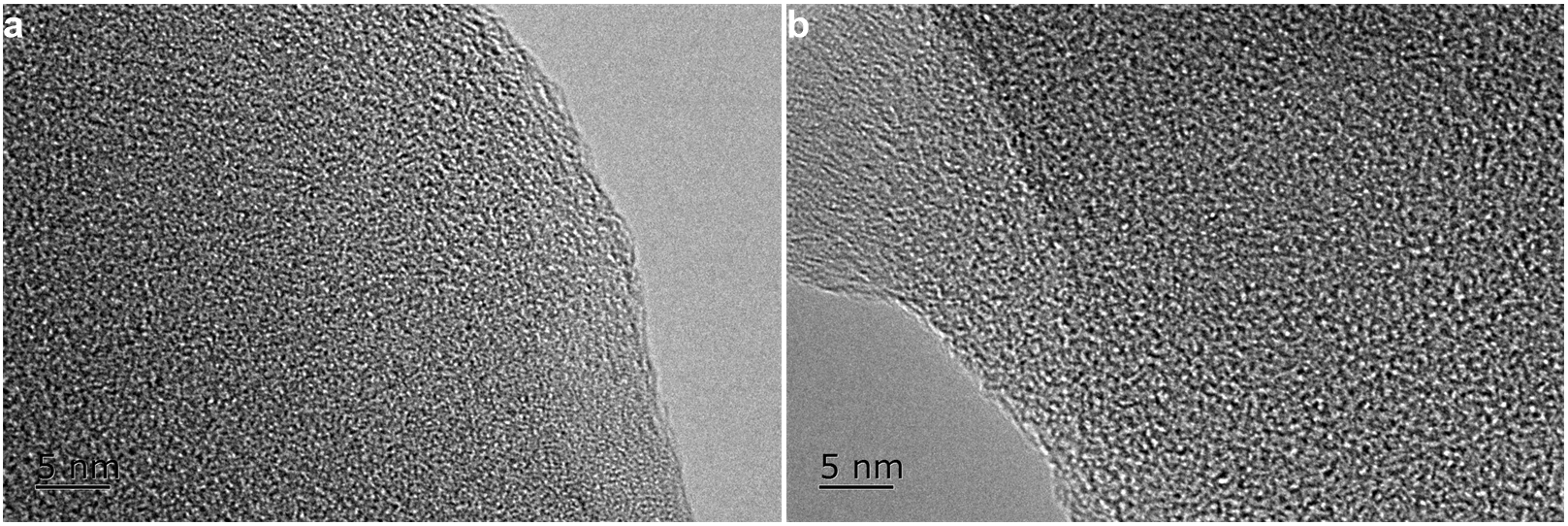

Supplement: Supplementary file 3 — Source Data [file 41467_2023_42720_MOESM3_ESM.zip › source data/Fig S14.png]

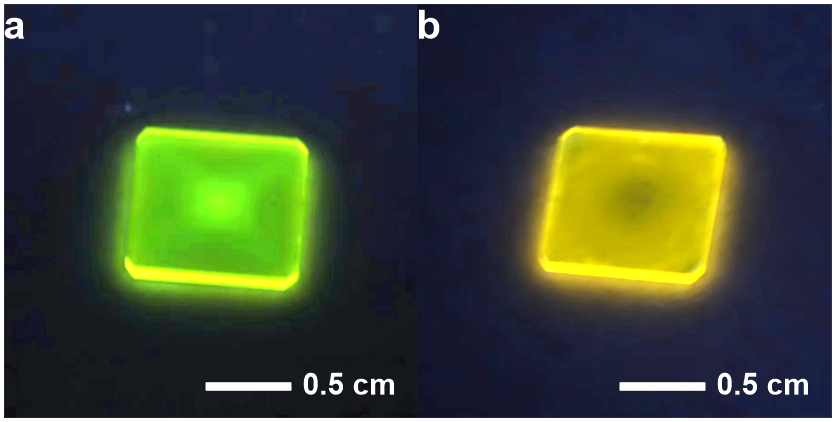

Supplement: Supplementary file 3 — Source Data [file 41467_2023_42720_MOESM3_ESM.zip › source data/Fig S18.png]

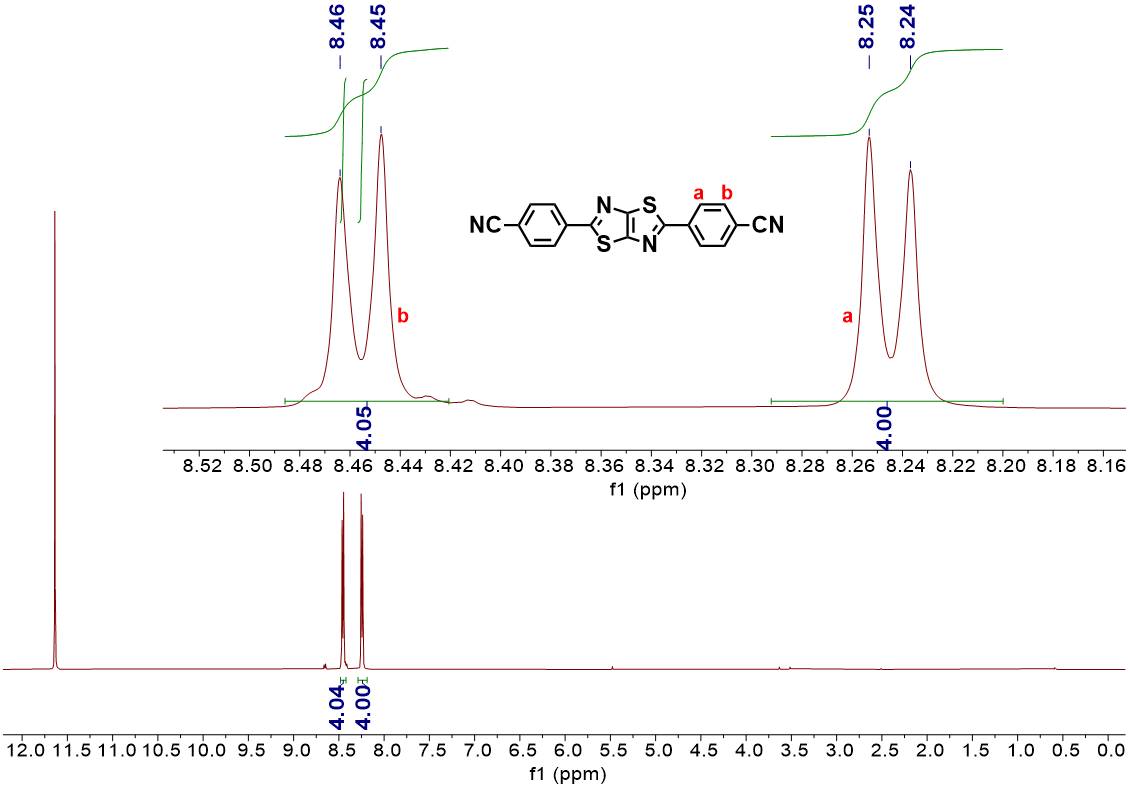

Supplement: Supplementary file 3 — Source Data [file 41467_2023_42720_MOESM3_ESM.zip › source data/Fig S2.png]

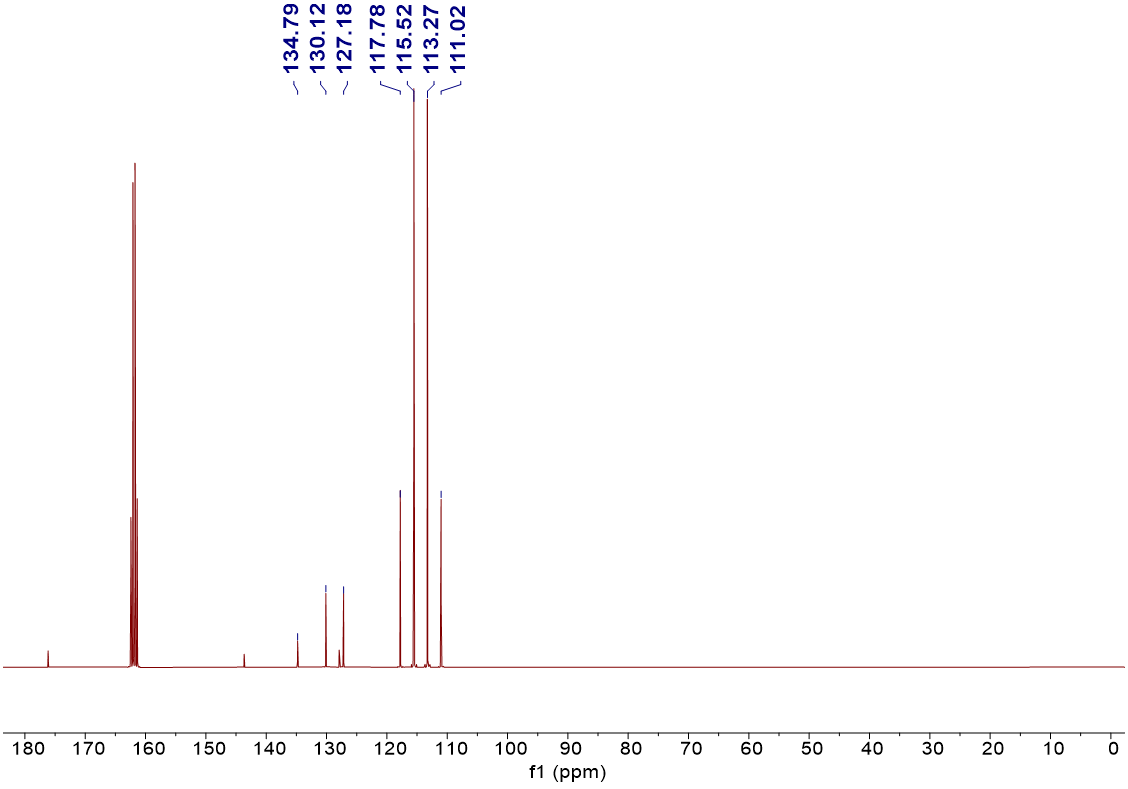

Supplement: Supplementary file 3 — Source Data [file 41467_2023_42720_MOESM3_ESM.zip › source data/Fig S3.png]

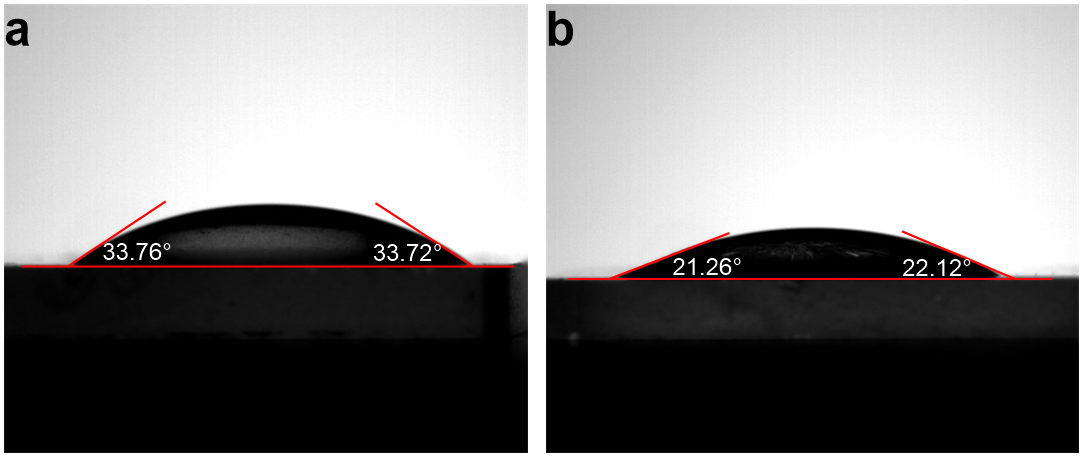

Supplement: Supplementary file 3 — Source Data [file 41467_2023_42720_MOESM3_ESM.zip › source data/Fig S34.png]

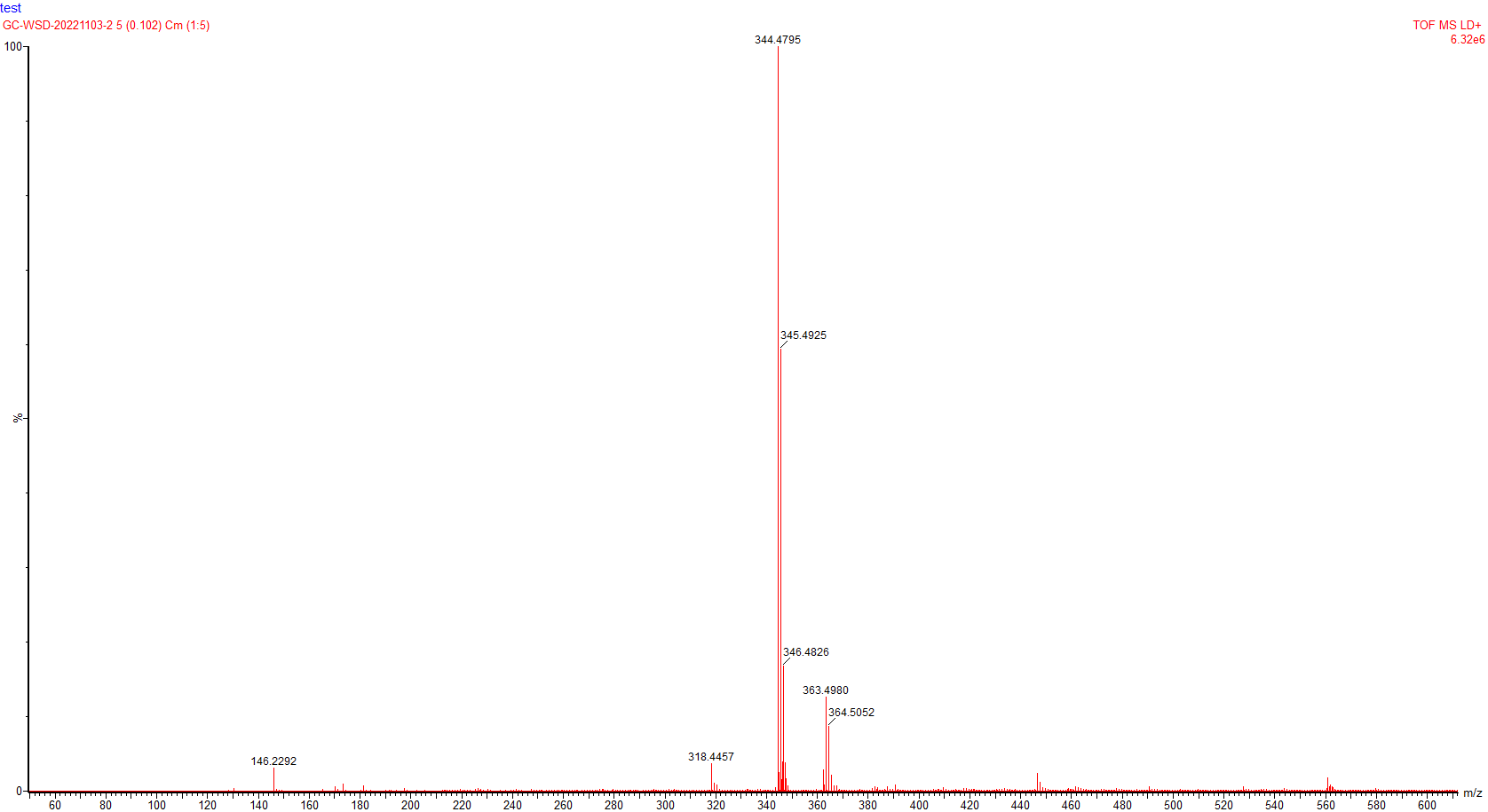

Supplement: Supplementary file 3 — Source Data [file 41467_2023_42720_MOESM3_ESM.zip › source data/Fig S4.png]

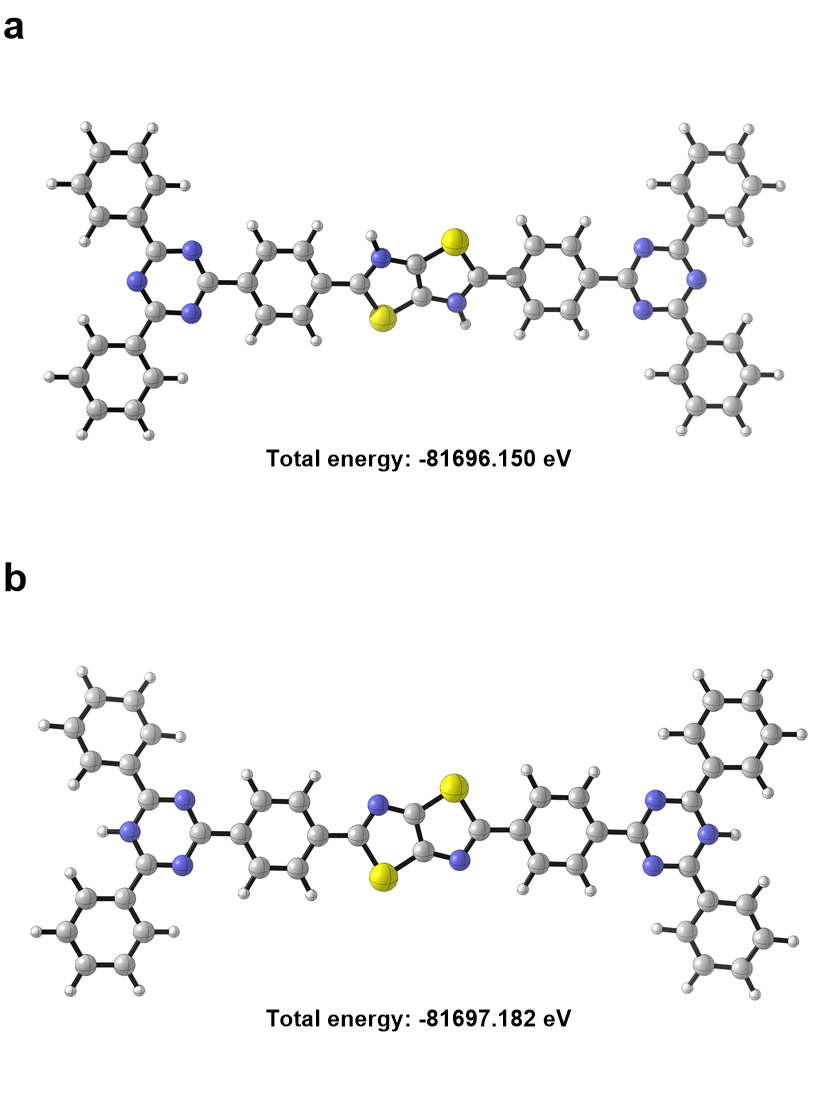

Supplement: Supplementary file 3 — Source Data [file 41467_2023_42720_MOESM3_ESM.zip › source data/Fig S42.png]

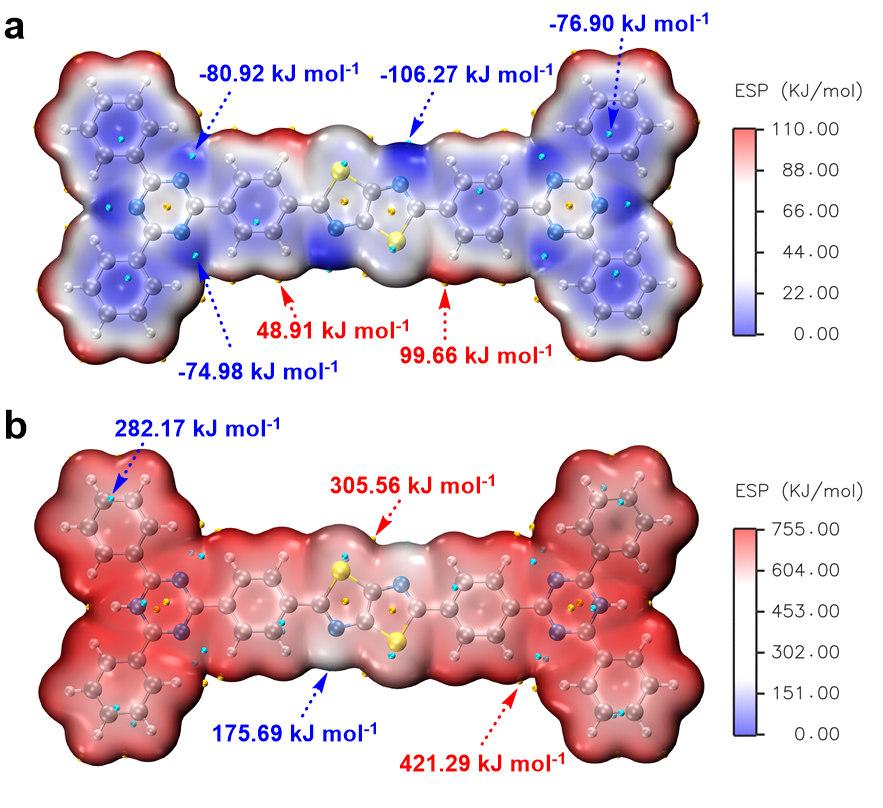

Supplement: Supplementary file 3 — Source Data [file 41467_2023_42720_MOESM3_ESM.zip › source data/Fig S43.png]

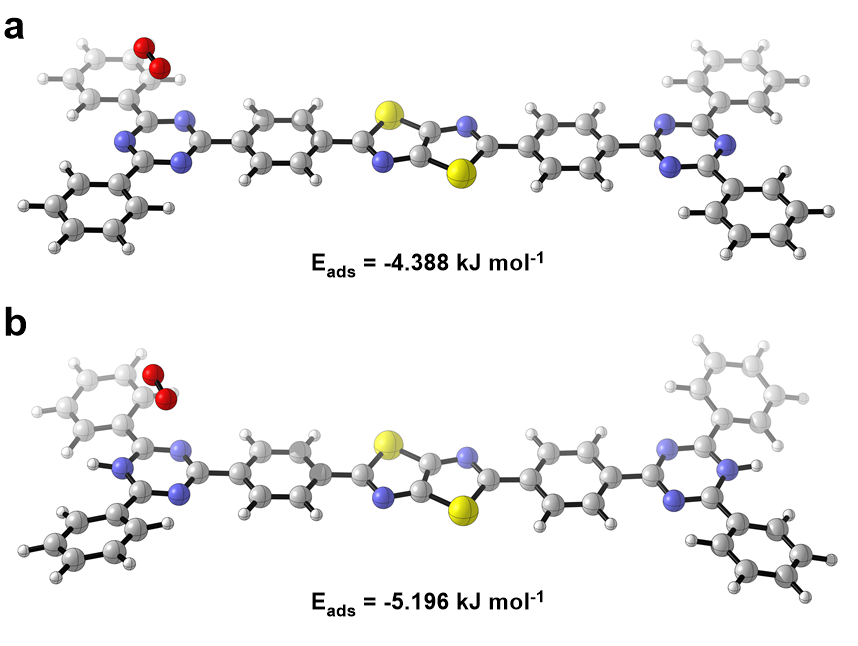

Supplement: Supplementary file 3 — Source Data [file 41467_2023_42720_MOESM3_ESM.zip › source data/Fig S44.png]

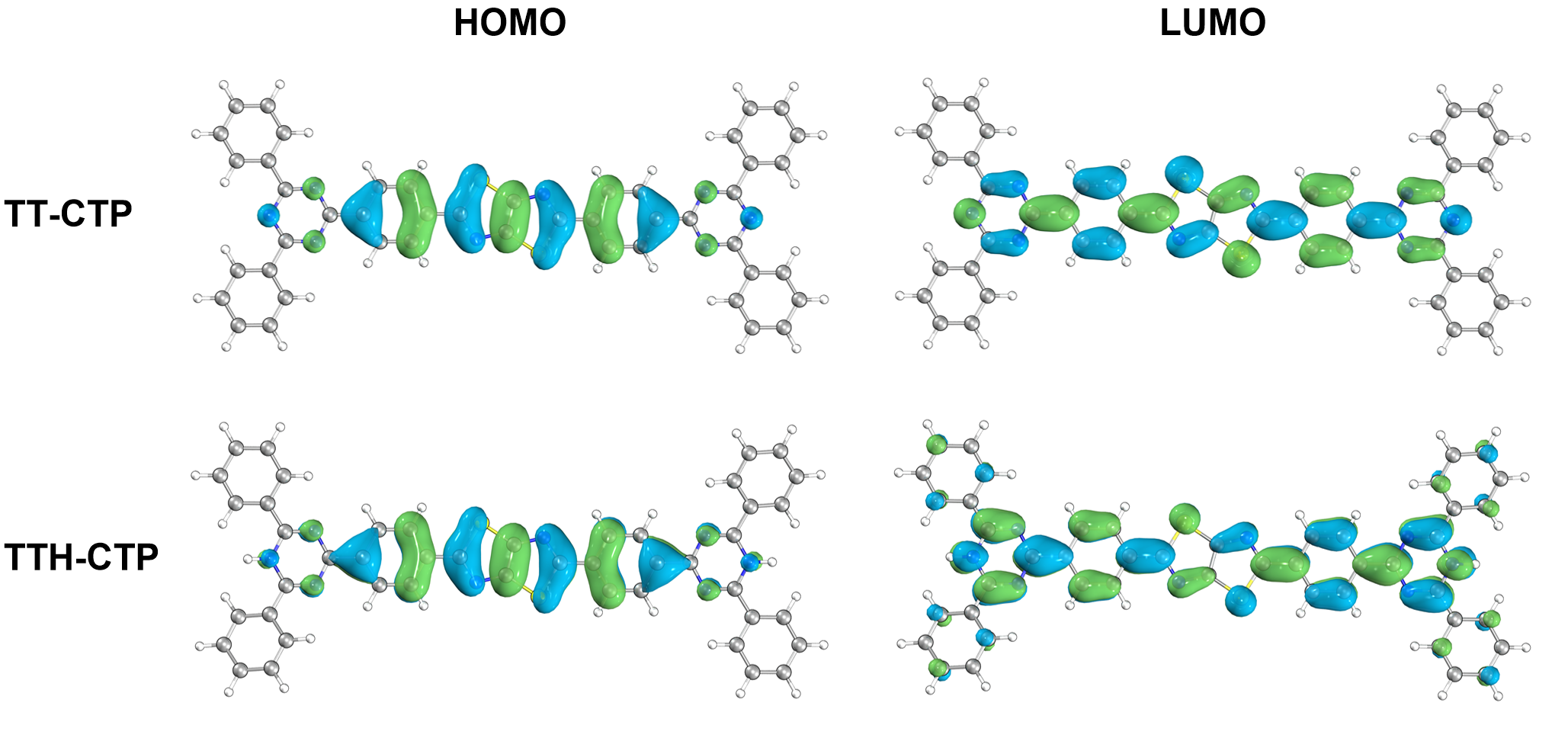

Supplement: Supplementary file 3 — Source Data [file 41467_2023_42720_MOESM3_ESM.zip › source data/Fig S45.png]

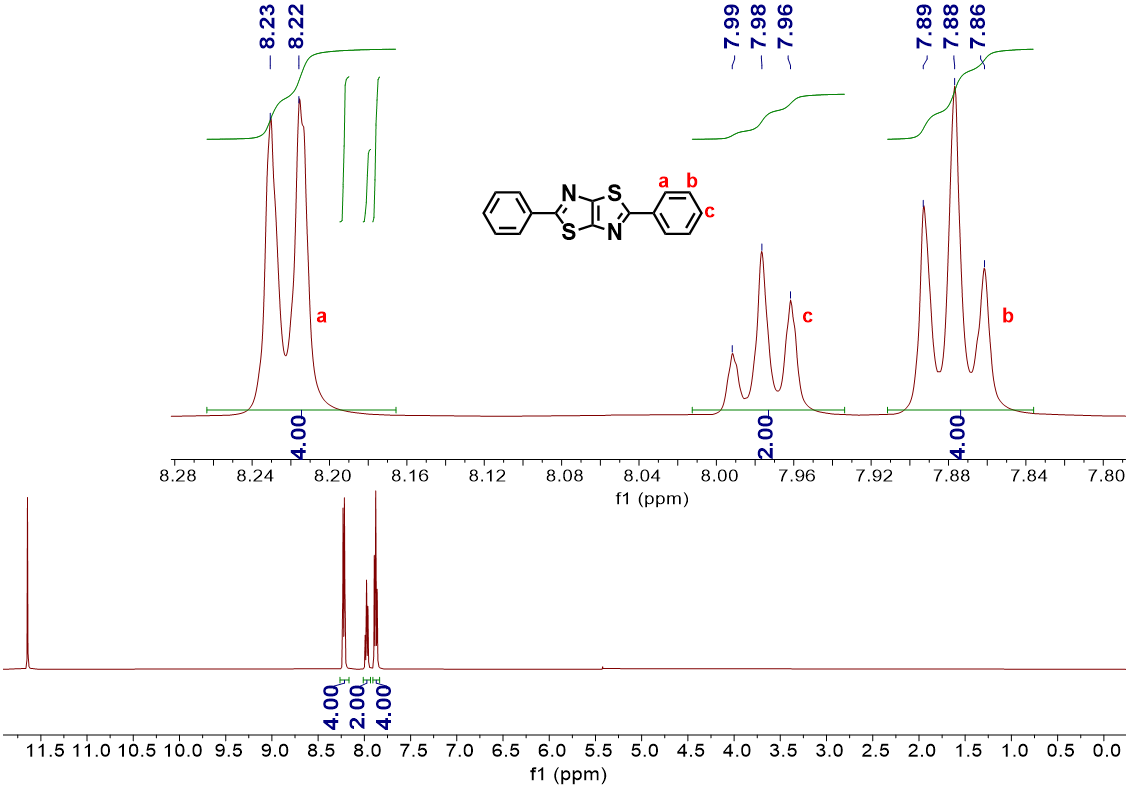

Supplement: Supplementary file 3 — Source Data [file 41467_2023_42720_MOESM3_ESM.zip › source data/Fig S5.png]

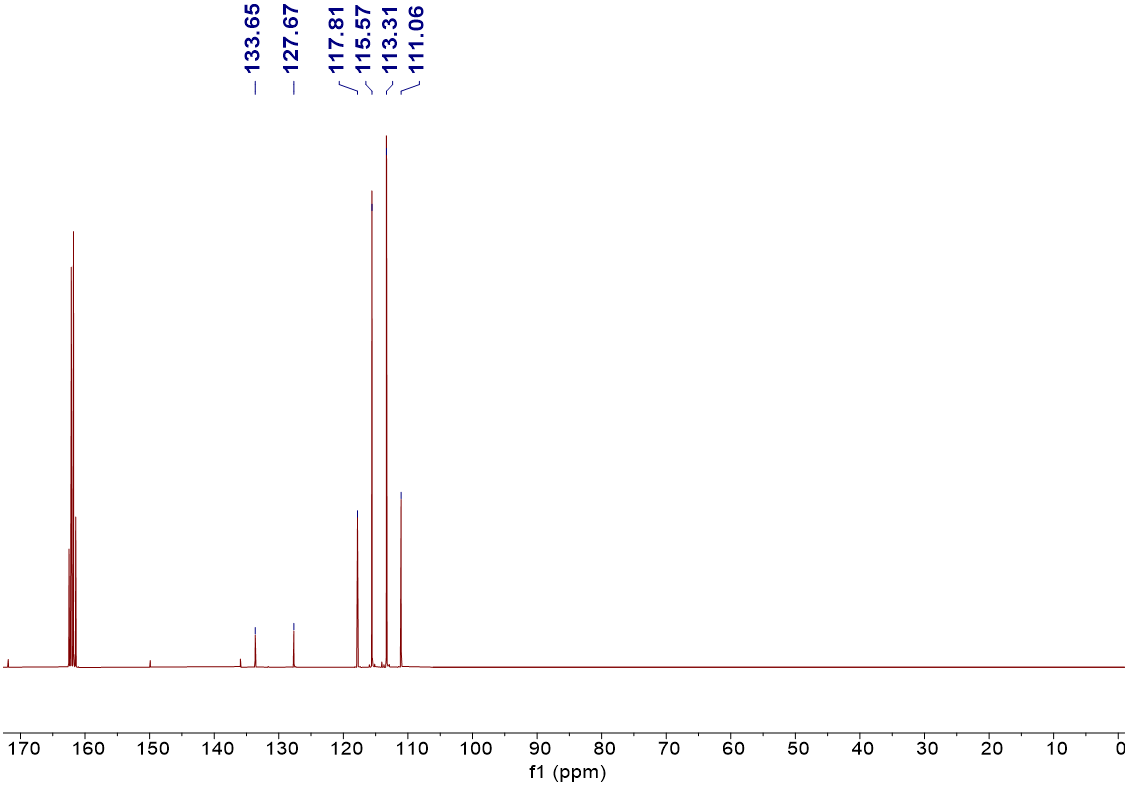

Supplement: Supplementary file 3 — Source Data [file 41467_2023_42720_MOESM3_ESM.zip › source data/Fig S6.png]

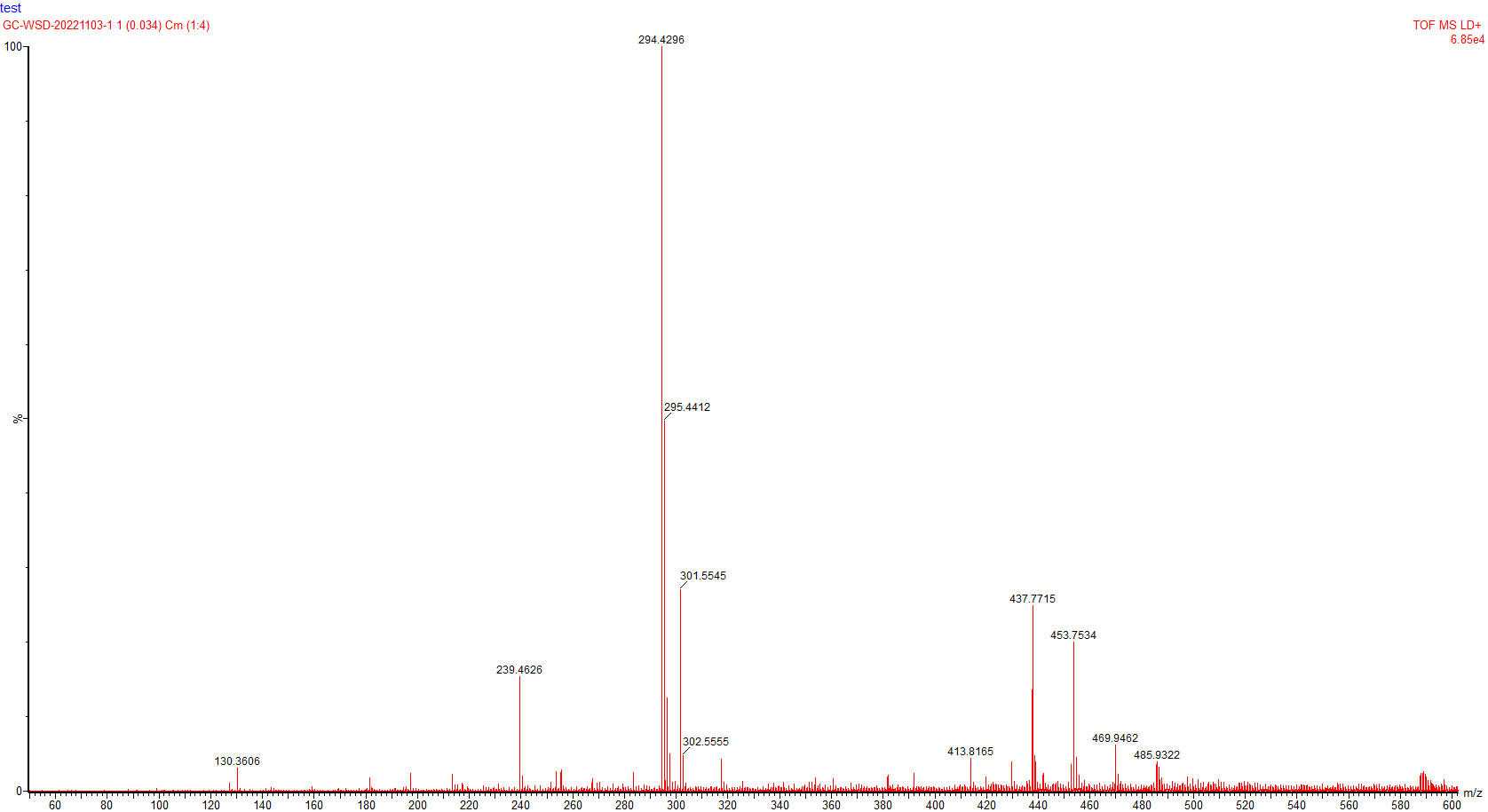

Supplement: Supplementary file 3 — Source Data [file 41467_2023_42720_MOESM3_ESM.zip › source data/Fig S7.png]

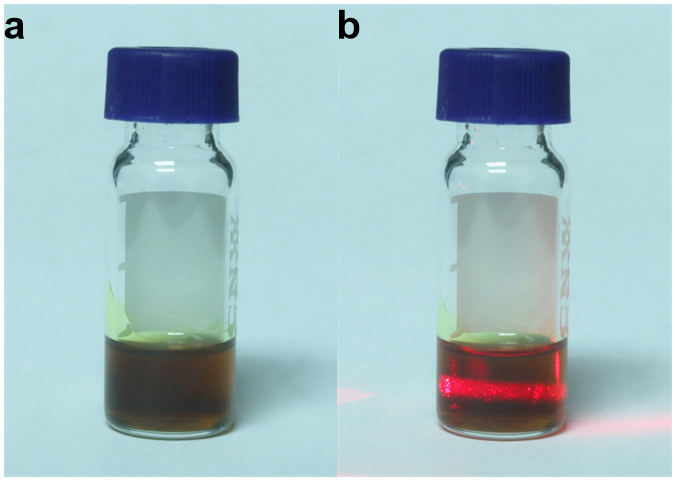

Supplement: Supplementary file 3 — Source Data [file 41467_2023_42720_MOESM3_ESM.zip › source data/Fig S8.png]
